# Supplementary material for: Divergent prebiotic synthesis of pyrimidine and 8-oxo-purine ribonucleotides
Source: Nat Commun. 2017 May 19;8:15270. doi: 10.1038/ncomms15270 (PMC5454461; doi:10.1038/ncomms15270)
Supplement: Supplementary Information — Supplementary figures, supplementary tables, supplementary methods and supplementary references. [file ncomms15270-s1.pdf]

Supplementary Methods

General: Reagents and solvents were obtained and used without further purification, unless specified, from the following commercial sources: *Sigma Aldrich*, *Alfa Aesar*, *Fluorochem*, *Acros Organics, Merck, Fisher Scientific, VWR International, Carbosynth, Manchester Organics, BDH, Lancaster, Apollo Scientific*, *Molekula*, *TCI* and *Santa Cruz Biotechnology*. Dowex® 50W×8 ion-exchange resin (200-400 mesh) ion exchange resin was purchased from *Acros Organics* and was washed with methanol and sodium hydroxide solution before being regenerated with hydrochloric acid solution. Deionized water was obtained from an *Elga Option 3* purification system. ^{1}H, ^{13}C and ^{31}P NMR spectra were recorded on *Bruker* NMR spectrometers *AVANCE III* 600, *AVANCE III* 400 and *AVANCE* 300 equipped with a *Bruker* 5 mm cryoprobe (600 MHz) and a gradient probe (400 and 300 MHz). All chemical shifts (*δ*) are reported in parts per million (ppm) relative to residual solvent peaks, and ^{1}H and ^{13}C chemical shifts relative to TMS and were calibrated using the residual solvent peak. When a mixed H_2O/D_2O solvent system was used a solvent suppression pulse sequence (noesygppr1d, *Bruker*) was used to obtain ^{1}H NMR spectra. Coupling constants are reported in Hertz (Hz). Spin multiplicities are indicated by symbols: s (singlet); d (doublet); t (triplet); q (quartet); qn (quintet); spt (septet); oct (octet), m (multiplet); obs. (obscured/coincidental signals), or a combination of these. Diastereotopic geminal (AB) spin systems coupled to one or two additional nuclei are reported as ABX and ABXY, respectively. NMR data are reported as follows: chemical shift (number of nuclei, multiplicity, coupling constants (*J*), nuclear assignment). Spectra were recorded at 298 K. Melting points were determined using an *Electrothermal* standard digital apparatus for all solids and are quoted to the nearest ºC and are uncorrected. Infrared spectra (IR) were recorded on a *Shimadzu IR Tracer 100* FT-IR spectrometer. Absorption maxima are reported in wavenumber (cm^{-1}). Ultraviolet spectra (UV) were recorded on a *Shimzadu UV-2700* spectrometer at 298K using a 1 mL quartz cuvette. Absorption maxima are reported in nm. Mass spectra and accurate mass measurements were recorded on a *VG70-SE*, *Waters LCT Premier XE* or *Thermo Finnigan MAT 900XP* instrument at the Department of Chemistry, University College London. Flash Column Chromatography (FCC) was carried out on a *Biotage Isolera One* purification platform using either *Biotage SNAP* or *Kinesis TELOS* cartridges. HPLC was carried out on an *Agilent 1260 Infinity LC* system using an *Agilent Polaris 5 C18-A 150×10.0 mm* column. Nucleotide cyclic phosphates were purified by elution with 100 mM aqueous ammonium formate at pH 4 (4 min at 5 mL/min) followed by a gradient elution with methanol (0% - 80%, over 8 min). Solution pH values were measured using a *Mettler Toledo Seven Compact* or a *Corning* 430 pH meter equipped with either a *Mettler Toledo InLab* semi-micro pH probe or a *Fisherbrand* FB68801 semi-micro pH probe. pD values were corrected according to *Covington et al.*[1] D_2-formamide was prepared by repeatedly (5×) lyophilising formamide (50 mL) with D_2O (100 mL) until the amide proton signals were no longer visible by ^{1}H NMR. Unless otherwise stated reactions conducted in formamide were repeatedly (3×) lyophilised with D_2O (10× volume of formamide) before analysis by ^{1}H NMR.

Synthesis of pentose oxazolidinone thiones **1a** – synthetic protocol

General procedure for synthesis of pentose oxazolidinone thiones, adapted from Girniene *et al.*[2] Pentose (1.00 g, 6.67 mmol) and potassium thiocyanate (1.30 g, 13.4 mmol) were dissolved in water (60 mL). HCl (37%, 1.15 mL) was added and the mixture was then incubated at 60 °C. NMR spectra were periodically acquired until the reaction was complete. Silica gel (10 mL, dry) was added and the mixture was then evaporated to dryness to give a free flowing powder. The powder was purified by FCC, eluting with EtOAc/MeOH 0-10%. The resulting product was recrystallized from $CHCl_3$/EtOH to yield the desired oxazolidinone thione. Analytical samples were recrystallised from hot water. (Supplementary Figs. 54-57).

Synthesis of pentose aminooxazolines (**10a**)

Arabinofuranosyl aminooxazoline (*arabino*-**10a**)

Cyanamide (**6**; 2.28 g, 54.2 mmol) was added to a syrup of arabinose (5.00 g, 33.3 mmol) in aqueous ammonia solution (3.5%, 50 mL). The resultant solution was heated at 60 °C for 1 h, then cooled to room temperature and MeOH (100 mL) was added to promote crystallisation. After 16 h at 4 °C the crystals were collected by filtration, washed with ice-cold MeOH (80 mL) and dried under vacuum to yield arabinofuranosyl aminooxazoline (*arabino*-**10a**; 3.20 g, 55%) as a fine white powder. M.p. 195–197 °C (Lit. 175 °C[3]). IR (solid, cm^{-1}) 3407 (NH_2), 3140 (OH), 2919 (CH), 1660 (C=N). ^1H NMR (600 MHz, D_2O) 5.91 (1H, d, *J* = 5.5 Hz, H1'), 4.93 (1H, dd, *J* = 5.5, 1.1 Hz, H2'), 4.33 (1H, dd, *J* = 3.4, 1.1 Hz, H3'), 4.02 (1H, ddd, *J* = 6.9, 5.5, 3.4 Hz, H4'), 3.60 (1H, ABX, *J* = 12.2, 5.5 Hz, H5'), 3.54 (1H, ABX, *J* = 12.2, 6.9 Hz, H5''). ^{13}C NMR (151 MHz, D_2O) 165.6 (C2), 99.29 (C1'), 89.3 (C4'), 85.0 (C2'), 75.9 (C3'), 61.8 (C5'). HRMS (*m/z*) calculated for $C_6H_{10}N_2O_4$ $[M+H^+]^+$, 175.0719; found, 175.0719.

Ribofuranosyl aminooxazoline (*ribo*-**10a**)

Cyanamide (**6**; 1.12 g, 26.6 mmol) was added to a syrup of ribose (2.00 g, 13.3 mmol) in aqueous ammonia solution (3.5%, 20 mL). The resultant solution was heated at 60 °C for 1 h, then cooled to room temperature and MeOH (40 mL) was added to promote crystallisation. After 16 h at 4 °C the crystals were collected by filtration, washed with ice-cold MeOH (30 mL) and dried under vacuum to furnish ribofuranosyl aminooxazoline (*ribo*-**10a**; 1.63 g, 70%) as a fine white powder. M.p. 182–184 °C (Lit. 195 °C[3]). IR (solid, cm^{-1}) 3426 (NH_2), 3319 (OH), 3147 (CH), 1664 (C=N). ^1H NMR (600 MHz, D_2O) 5.80 (1H, d, *J* = 5.3 Hz, H1'), 4.98 (1H, t, *J* = 5.3 Hz, H2'), 4.13 (1H, dd, *J* = 9.6, 5.3 Hz, H3'), 3.93 (1H, ABX, *J* = 12.7, 2.3 Hz, H5'), 3.73 (1H, ABX, *J* = 12.7, 4.6 Hz, H5''), 3.62 (1H, ddd, *J* = 9.6, 4.6, 2.3 Hz, H4'). ^{13}C NMR (151 MHz, D_2O) 166.7 (C2), 97.7 (C1'), 82.6 (C4'), 77.9 (C2'), 71.0 (C3'), 60.2 (C5'). HRMS (*m/z*) calculated for $C_6H_{10}N_2O_4$ $[M+H^+]^+$, 175.0719; found, 175.0721.

Synthesis of pentose oxazolidinones (**22**)

Arabinofuranosyl oxazolidinone (*arabino*-**22**)

To a solution of arabinose (3.00 g, 20.0 mmol) in H_2O (20.0 mL) was added NH_4Cl (1.60 g, 30.0 mmol) and potassium cyanate (2.40 g, 30.0 mmol). The resultant solution was heated at 60 ^oC for 8 h, then allowed to cool to room temperature. The solution was lyophilised to give a yellow oil, which was purified by silica gel FCC eluting with EtOAc/MeOH (9:1). After evaporation the residue was crystallised from EtOH/H_2O to give arabinofuranosyl oxazolidinone (*arabino*-**22**; 1.60 g, 46%) as a white solid. M.p. 164–168 ^oC. IR (solid, cm^{-1}) 3398 (NH), 3233 (OH), 2956, 2935, 2897 (CH), 1747 (C=O). ^1H NMR (600 MHz, D_2O) 5.92 (1H, d, J = 5.7 Hz, H1'), 5.08 (1H, d, J = 5.7 Hz, H2'), 4.44 (1H, d, J = 2.4 Hz, H3'), 4.16 (1H, ddd, J = 7.0, 4.9, 2.4 Hz, H4'), 3.70 (1H, ABX, J = 12.4, 4.9 Hz, H5'), 3.60 (1H, ABX, J = 12.4, 6.0 Hz, H5''). ^{13}C NMR (151 MHz, D_2O) 160.1 (C2), 87.6 (C1'), 87.2 (C4'), 86.9 (C3'), 75.5 (C2'), 61.9 (C5'). HRMS (*m/z*) calculated for $C_6H_9NO_5$ $[M+H^+]^+$, 176.0559; found, 176.0551.

Ribofuranosyl oxazolidinone (*ribo*-**22**)

To a solution of ribose (3.00 g, 20.0 mmol) in H_2O (20 mL) was added NH_4Cl (1.60 g, 30.0 mmol) and potassium cyanate (2.40 g, 30.0 mmol). The resultant mixture was heated at 60 ^oC for 8 h, allowed to cool to room temperature and lyophilised to give a yellow oil. The oil was then purified by silica gel FCC eluting with (EtOAc/MeOH 9:1). After evaporation the residue was recrystallised from EtOH/H_2O to give ribofuranosyl oxazolidinone (*ribo*-**22**; 2.20 g, 63%) as a yellow solid. M.p. 125–127 ^oC (Lit. 161–164 ^oC[4]). IR (solid, cm^{-1}) 3341 (NH), 3217 (OH), 3005, 2934, 2879 (CH), 1715 (C=O). ^1H NMR (600 MHz, D_2O) 5.80 (1H, d, J = 5.4 Hz, H1'), 5.13 (1H, t, J = 5.4 Hz, H2'), 4.18 (1H, dd, J = 9.5, 5.4 Hz, H3'), 3.96 (1H, ABX, J = 12.7, 2.3 Hz, H5'), 3.88 (1H, ddd, J = 9.5, 4.9, 2.3 Hz, H4'), 3.75 (1H, ABX, J = 12.7, 4.9 Hz H5''). ^{13}C NMR (151 MHz, D_2O) 161.1 (C2), 86.0 (C1'), 80.4 (C4'), 78.6 (C2'), 70.4 (C3'), 60.0 (C5'). HRMS (*m/z*) calculated for $C_6H_9NO_5$ $[M+H^+]^+$, 176.0559; found, 176.0555.

Synthesis of cyanoacetylene (**8**)

Propiolamide (**23**)

Methyl propiolate (25.0 g, 24.6 mL, 297 mmol) was added to liquid ammonia (100 mL) at -78 ^oC and stirred. After 1 h excess ammonia was evaporated by warming the solution to room temperature, followed by co-evaporation with dry chloroform, to yield white solids. The solids were dissolved in hot anhydrous CH_2Cl_2 (30 mL) and left at -18 ^oC to yield propiolamide (**23**; 5.74 g, 74%) as long colourless needle-like crystals. M.p. 57–59 ^oC (Lit 58-61 ^oC[5]). ^1H NMR (600 MHz, D_2O) 3.53 (1H, s, H-(C3)). Spectra match literature data[5]

Cyanoacetylene (**8**)

Propiolamide (**23**; 5.00 g, 72.5 mmol) and oven dried sand (37.5 g) were thoroughly ground together with a pestle and mortar. P_2O_5 (15.5 g, 109 mmol) was added and rapidly mixed before the mixture was transferred to a dry flask under argon atmosphere. Cyanoacetylene **8** was then distilled from the sticky mixture at 130 ^oC and 100 torr over 1 h. During this time a white solid (**8**; 2.98 g, 80%) was collected at -78 ^oC. The solid was immediately dissolved in water (58 mL) to give a 1M aqueous solution of cyanoacetylene **8**. ^1H NMR (600 MHz, D_2O) 3.42 (1H, s, H-(C3)). ^{13}C NMR (151 MHz, D_2O) 105.5 (C1), 77.1 (C3), 56.7 (C2).

Synthesis of arabinofuranosyl-(2-thiomethyl)-oxazolidinone (arabino-**1c**) – synthetic protocol

Arabinofuranosyl-(2-thiomethyl)-oxazolidinone (*arabino*-**1c**) was synthesised using a protocol adapted from Davidson *et al.*[6] Arabinofuranosyl oxazolidinone thione (*arabino*-**1a**; 7.00 g, 36.4 mmol), iodomethane (5.84 g, 41.2 mmol) and NaOH (1.65 g, 41.2 mmol) were vigorously stirred in ethanol (280 mL) and H_2O (140 mL) at room temperature for 30 min. The solution was then concentrated and purified by FCC eluting with $CHCl_3$/MeOH (9:1) to yield arabinofuranosyl-(2-thiomethyl)-oxazolidinone (*arabino*-**1c**; 6.2 g, 84%) as a white solid. M.p. 84–78 ^{o}C. IR (solid, cm^{-1}) 3289 (OH), 2895 (CH), 1585 (C=N). ^{1}H NMR (600 MHz, D_2O) 6.10 (1H, d, J = 5.9 Hz, H1'), 5.09 (1H, dd, J = 5.9, 1.1 Hz, H2'), 4.38 (1H, dd, J = 3.2, 1.1 Hz, H3'), 4.11 (1H, ddd, J = 7.0, 5.2, 3.2 Hz, H4'), 3.59 (1H, ABX, J = 12.2, 5.2 Hz, H5'), 3.51 (1H, ABX, J = 12.2, 7.0 Hz, H5''), 2.51 (3H, s, CH_3). ^{13}C NMR (151 MHz, D_2O) 174.5 (C2), 100.1 (C1'), 90.9 (C2'), 86.1 (C4'), 76.0 (C3'), 61.6 (C5'), 14.2 (C4). HRMS (m/z) calculated for C7H12NO4S $[M+H^{+}]^{+}$, 206.0487; found, 206.0474. Data matches literature data.[6]

Measurement of pKa values

Solutions of the desired substrate (50mM) with 4,4-dimethyl-4-silapentane-1-sulfonic acid (DSS, 10mM) in sodium citrate buffer (250mM, 10% D_2O) were adjusted to the desired pH using HCl/NaOH (4M) and submitted to analysis by ^{1}H NMR. The delay between pH adjustments and ^{1}H NMR measurement was typically 10-15 min. The trimethylsilyl peak of DSS was set to 0 ppm and the chemical shift of the relevant proton was recorded. Chemical shift was plotted against pH across a range of pH's to give the pKa curve. (Supplementary Figs. 8-12).

General procedure for reaction of nucleophiles (**2**) with activated pentose oxazolidinone thiones (**1b-c**)

The nucleophile (**2**; 0.25 mmol) was dissolved in D_2O (400 µL) at the desired pD. Thione (**1b** or **1c**; 0.125 mmol) and 4,4-dimethyl-4-silapentane-1-sulfonic acid (DSS, 50 µL, 244 mM; internal standard) were added. The solution was readjusted to the desired pD and the pD was maintained over the course of the reaction by addition of 1M NaOD/DCl. The reaction was monitored by periodic acquisition of ^{1}H NMR spectra. Each spectrum was acquired upon dilution of an aliquot (20 µL) of the reaction mixture with D_2O (430 µL). Cyclisation was induced in an aliquot (50 µL) of the reaction mixture adjusted to pD 9 by addition of ammonium hydroxide (450 µL, 100mM, D_2O). (Supplementary Figs. 20-28).

Nucleophile additions to cyanovinyl-thiones **1b** produced a white precipitate that was isolated and characterised as *S*-β-dicyanovinylsulfide (**15**).

S-β-Dicyanovinyl sulfide (**15**)

M.p. 140–147 ^{o}C. IR (Solid, cm^{-1}) 3073, 3061, 3046 (=C-H), 2214 (CN), 1578 (C=C), 1547 (C-C). ^{1}H NMR (600 MHz, DMSO) δ 8.05 (2H, d, J = 10.3 Hz, H1), 6.12 (2H, d, J = 10.3 Hz, H2). ^{13}C NMR (151 MHz, DMSO) δ 147.2 (C1), 115.4 (C3), 97.2 (C2). *m/z* (CI+) 137

(100%, M+). Crystallographic and refinement parameters are shown in Supplementary Table 5. (Supplementary Fig. 65).

Synthesis of 2,2'-anhydro-5-aminoimidazole-4-carbonitrile-β-furanosylarabinoside (**16b**) – synthetic protocol

3',5'-Bisacetoxy-2,2'-anhydro-5-aminoimidazole-4-carboxamide-*β*-furanosylarabinoside (**25**)

2,2'-Anhydro-5-aminoimidazole-4-carboxamide-*β*-furanosylarabinoside (**16c**; 600mg, 2.34 mmol) was suspended in water (50 mL) at RT, the solution was adjusted to pH 8 with $NaOH_{(aq)}$ and *N*-acetylimidazole (3.87 g, 35.2 mmol) was added. The reaction was stirred at RT for 30 min and kept at pH 8 by manual drop wise addition of 8M $NaOH_{(aq).}$ The mixture was concentrated onto silica gel (10 mL) and purified by silica gel FCC, eluting with $CHCl_3$/MeOH (1:0 – 9:1) to yield 3',5'-bisacetoxy-2,2'-anhydro-5-aminoimidazole-4-carboxamide-*β*-furanosylarabinoside (**25**; 314mg, 0.92 mmol, 39%) as an off white foam. M.p. 163–166 ^oC. IR (Solid, cm^{-1}) 3330 (NH), 1740 (OAc), 1649, 1597 (C=N). ^1H NMR (600 MHz, D_2O) 6.52 (1H, d, J = 5.7 Hz, H1'), 5.91 (1H, d, J = 5.7 Hz, H2'), 5.52 (1H, d, J = 1.9 Hz, H3'), 4.75 (1H, dt, J = 1.9, 4.1 Hz, H4'), 4.20 (2H, d, J = 4.1 Hz, H5', H5''), 2.20 (3H, s, OAc), 1.98 (3H, s, OAc). ^{13}C NMR (151 MHz, D_2O) 174.3 (5'OAc), 173.5 (3'OAc), 169.0 (C6), 152.2 (C2), 140.2 (C5), 109.7 (C4), 96.0 (C2'), 87.3 (C1'), 84.7 (C4'), 78.7 (C3'), 64.4 (C5'), 20.8 (3'OAc), 20.3 (5'OAc). HRMS (*m/z*) calculated for $C_{13}H_{16}N_4O_7$ $[M+H^+]^+$, 341.1092; found, 341.1090. (Supplementary Fig. 72).

3',5'-Bisacetoxy-2,2'-anhydro-5-aminoimidazole-4-carbonitrile-*β*-furanosylarabinoside (**26**)

3',5'-Bisacetoxy-2,2'-anhydro-5-aminoimidazole-4-carboxamide-*β*-furanosylarabinoside (**25**; 250 mg, 0.74 mmol) was dissolved in pyridine (20 mL) and cooled to 0 ^oC. *p*-Toluenesulfonyl chloride (561 mg, 2.94 mmol) was added in a single portion and the reaction was stirred for 2 h. The reaction was quenched with water (5 mL) at 0 ^oC, and silica gel (5 mL, dry) was added. The slurry was concentrated to a dry free flowing powder and purified by silica gel FCC, eluting with $CHCl_3$/MeOH (1:0 – 9:1) to yield 3',5'-bisacetoxy-2,2'-anhydro-5-aminoimidazole-4-carbonitrile-*β*-furanosylarabinoside (**26**; 150 mg, 0.47 mmol, 64%) as an off white foam. M.p. 180–181 ^oC. IR (Solid, cm^{-1}) 3161 (NH), 2197 (C≡N), 1742 (OAc), 1649, 1574 (C=N). ^1H NMR (600 MHz, MeOD) 6.37 (1H, d, J = 5.5 Hz, H1'), 5.77 (1H, d, J = 5.5 Hz, H2'), 5.38 (1H, d, J = 1.8 Hz, H3'), 4.55 (1H, ddd, J = 4.6, 4.3, 1.8 Hz, H4'), 4.16 (1H, ABX, J = 12.2, 4.6 Hz, H5'), 4.03 (1H, ABX, J = 12.2, 4.3 Hz, H5''), 2.12 (3H, s, OAc), 1.98 (3H, s, OAc). ^{13}C NMR (151 MHz, MeOD) 172.1 (3'OAc), 171.5 (5'OAc), 152.0 (C2), 145.3 (C5), 117.5 (C6), 97.1 (C2'), 88.5 (C4), 88.1 (C1'), 86.0 (C4'), 79.0 (C3'), 64.6 (C5'), 20.5 (3'OAc), 20.3 (5'OAc). HRMS (*m/z*) calculated for $C_{13}H_{14}N_4O_6$ $[M+H^+]^+$, 323.0986; found, 323.0986. (Supplementary Fig. 73).

2,2'-Anhydro-5-aminoimidazole-4-carbonitrile-*β*-furanosylarabinoside (**16b**)

3',5'-Bisacetoxy-2,2'-anhydro-5-aminoimidazole-4-carbonitrile-*β*-furanosylarabinoside (**26**; 170 mg, 0.53 mmol) was dissolved in saturated methanolic ammonia (20 mL) and allowed to stand for 30 min at RT. The solution was concentrated to dryness, dissolved in dry methanol (20 mL) and again concentrated. This residue was twice co-evaporated with methanol (20 mL). The residue was dissolved in methanol (1 mL) and then acetone (20 mL) was added

with rapid stirring. The precipitated product was isolated by centrifugation, washed with acetone and dried under vacuum to yield 2,2'-anhydro-5-aminoimidazole-4-carbonitrile-β-furanosylarabinoside (**16b**; 101 mg, 0.42 mmol, 80%) as a pale yellow solid.

Synthesis of 8,2'-*O*-anhydro-9-*β*-arabinofuranosyl-cycloadenosine (**17A**) - synthetic protocol

8-Bromoadenine-9-*β*-arabinofuranoside (**27**)

To a solution of adenine 9-*β*-arabinofuranoside (10.00 g, 34.4 mmol) dissolved in aqueous sodium acetate (1.5 L, 1M, pH 4.0) at RT was added saturated bromine water (100 mL). The resultant solution was vigorously stirred at RT for 3 h. The solution was decolourised with 5M sodium bisulfite and then adjusted to pH 7.0 with 5M NaOH. The decolourised solution was then concentrated under a stream of air and after 16 h a white crystalline precipitate had formed. The resulting crystals were collected by filtration and washed with water (3 × 50 mL) and acetone (100 mL) to give 8-bromoadenine-9-*β*-arabinofuranoside (**27**; 6.72 g, 19.4 mmol, 56%) as a white powder. An analytically pure sample was obtained by recrystallisation from EtOH/H_2O. M.p. 203 ºC decomp (Lit.[7] 202–204 ºC, decomp). IR (Solid, cm^{-1}) 3375 (NH_2), 3177 (OH), 2866 (CH), 1736 (C=C), 1603 (C=N). ^1H NMR (600 MHz, DMSO) 8.07 (1H, s, H2), 7.46 (2H, br s, NH_2), 6.23 (1H, d, *J* = 7.1 Hz, H1'), 5.64 (1H, d, *J* = 5.8 Hz, 2'OH), 5.49* (1H, d, *J* = 5.7 Hz, 3'OH), 5.35 (1H, dd, *J* = 6.5, 4.4 Hz, 5'OH), 4.45 (1H, td, *J* = 7.1, 5.7 Hz, H3'), 4.34 (1H, td, *J* = 7.1, 5.8 Hz, H2'), 3.83 – 3.68 (3H, m, H4', H5', H5''). ^{13}C NMR (151 MHz, DMSO) 155.0 (C6), 152.0 (C2), 150.4 (C4), 126.3 (C8), 119.3 (C5), 86.2 (C1'), 82.8 (C4'), 76.5 (C2'), 74.1 (C3'), 60.9 (C5'). HRMS (*m/z*): $[M-H^+]^-$ $C_{10}H_{12}BrN_5O_4$ calcd 346.0151, found 346.0133. Spectra match literature data[7]. (Supplementary Fig. 74).

8,2'-*O*-Anhydro-9-*β*-arabinofuranosyl-cycloadenosine (**17A**)[8]

8-Bromoadenine-9-*β*-arabinofuranoside (**27**; 4.0 g, 11.6 mmol) and ammonium formate (3.0 g, 47.6 mmol) were suspended in H_2O (800 mL) and the solution was adjusted to pH 9 with concentrated ammonium hydroxide. The mixture was stirred overnight, neutralised with concentrated formic acid and the solution concentrated to approximately 100 mL under a stream of air. The precipitate was filtered off and washed with ice cold H_2O (50 mL), acetone (200 mL) and dried in vacuo to give 8,2'-*O*-anhydro-9-*β*-arabinofuranosyl-cycloadenosine (**17A**; 2.1 g, 7.9 mmol, 68%) as a fine white powder. An analytical sample was obtained by recrystallisation from hot water.

2,2'-Anhydro-5-aminoimidazole-4-carbonitrile-*β*-furanosylarabinoside (**16b**; 4.8 mg, 0.02 mmol), 2,2'-anhydro-5-aminoimidazole-4-carboxamide-*β*-furanosylarabinoside **16c** (5.0 mg, 0.02 mmol), formamidine hydrochloride (16.1 mg, 0.2 mmol) and 4,4-dimethyl-4-silapentane-1-sulfonic acid (DSS, internal standard, 5 mg) were dissolved in D_2-formamide (500 µL). The mixture was heated at 100 ºC and NMR spectra were periodically acquired. 2,2'-Anhydro-5-aminoimidazole-4-carbonitrile-*β*-furanosylarabinoside (**16b**) had been mostly consumed after 5 h. The reaction was concentrated to dryness, D_2O (5 mL) was added and the mixture was lyophilised. The residue was then dissolved in d_6-DMSO and NMR spectra were acquired. Spiking with authentic samples of 8,2'-*O*-anhydro-9-*β*-arabinofuranosyl-

cycloadenosine (**17A**), 8,2'-*O*-anhydro-9-*β*-arabinofuranosyl-cycloinosine (**17I**) and calibration to the internal standard (DSS) confirmed yields of 60% **17A** and 4% **17I** with 13% **16b** and 36% **16c** remaining.

Synthesis of 8,2'-O-anhydro-9-β-arabinofuranosyl-cycloinosine (**17I**) - synthetic protocol

8,2'-*O*-Anhydro-9-*β*-arabinofuranosyl-cycloadenosine (**17A**; 265 mg, 1.00 mmol) was dissolved in acetic acid (20 mL, 2M) and sodium nitrite (345 mg, 5 mmol) in H_2O (5 mL) was added. The reaction was stirred at RT for 24 h, then the solvent was evaporated in vacuo and the residue repeatedly co-evaporated with toluene, until no odour of acetic acid remained. The residue was crystallised from H_2O to give 2',8-*O*-anhydro-9-*β*-arabinofuranosyl-cycloinosine (**17I**; 180 mg, 0.68 mmol, 68%) as pale yellow plates.

Isomerisation of anhydronucleosides

8,5'-*O*-Anhydro-9-*β*-arabinofuranosyl-cycloadenosine (**19**)

2',8-*O*-Anhydro-9-*β*-arabinofuranosyl-cycloadenosine (**17A**; 265 mg, 1.0 mmol) was heated in 0.01M NaOH (30 mL) at 60 ^{o}C for 3 h. The solution was then neutralised with 0.1M HCl and concentrated in vacuo to approximately 5 mL, leading to the precipitation of a white crystalline solid. The solids were isolated by filtration, washed with MeOH (5 mL) and recrystallised from water to give 8,5'-*O*-anhydro-9-*β*-arabinofuranosyl-cycloadenosine (**19**; 29 mg, 0.11 mmol, 11%) as white prism-like crystals. M.p. 236 ^{o}C (decomp.). IR (Solid, cm^{-1}) 3563 (NH), 3420 (OH), 2961, 2916 (CH), 1620 (C=C), 1576 (C=N). ^{1}H NMR (600 MHz, D_6-DMSO) 8.06 (1H, s, H2), 6.30 (1H, d, *J* = 6.4 Hz, H1'), 4.53 (1H, ABX, *J* = 13.0, 2.6 Hz, H5'), 4.38 (1H, d, *J* = 6.4 Hz, H2'), 4.35 (1H, d, *J* = 2.6 Hz, H4'), 4.18 (1H, s, H3'), 4.12 (1H, AB, *J* = 13.0 Hz, H5''). ^{13}C NMR (151 MHz, DMSO) 155.0 (C8), 153.9 (C6), 151.4 (C2), 148.4 (C4), 114.2 (C5), 86.6 (C4'), 83.8 (C2'), 79.8 (C1'), 77.4 (C3'), 74.7 (C5'). HRMS (*m/z*): $[M-H^{+}]^{-}$ $C_{10}H_{11}N_5O_4$ calcd 266.0889, found 266.0898. Crystallographic and refinement parameters are shown in Supplementary Table 6. (Supplementary Fig. 70).

2',3'-Epoxy-9-*β*-ribofuranoside-8-oxoadenosine (**20**)

9-*β*-Arabinofuranoside-8-bromoadenine (**27**; 1.0 g, 2.89 mmol) was dissolved in NaOH (1M, 10 mL) and stirred for 2 h at RT. Neutralised silica (6.0g in 1.50 mL ammonium hydroxide) was added to the reaction and the slurry was concentrated in vacuo to yield a fine powder. The product was then purified by FCC, eluting with ethyl acetate/methanol (95:5) to yield 2',3'-epoxy-9-*β*-ribofuranoside-8-oxoadenosine (**20**; 260 mg, 0.98 mmol, 34%) as a white crystalline solid. An analytically pure sample was obtained by recrystallisation of the product from hot water. M.p. 202 ^{o}C (decomp.). IR (Solid, cm^{-1}) 3323 (NH_2), 3194 (OH), 1715 (C=O), 1651 (C=C), 1595 (C=N). ^{1}H NMR (600 MHz, D_2O) 8.13 (1H, s, H2), 6.17 (1H, s, H1'), 4.56 (1H, d, *J* = 2.7 Hz, H2'), 4.40 (1H, dd, *J* = 7.7, 5.0 Hz, H4'), 4.27 (1H, d, *J* = 2.7 Hz, H3'), 3.82 (1H, ABX, *J* = 12.1, 5.0 Hz, H5'), 3.72 (1H, ABX, *J* = 12.1, 7.7 Hz, H5''). ^{13}C NMR (151 MHz, D_2O) 153.5 (C8), 151.8 (C2), 148.2 (C6), 147.1 (C4), 105.3 (C5), 81.5 (C4'), 81.3 (C1'), 61.4 (C5'), 59.9 (C3'), 58.5 (C2'). HRMS (*m/z*): $[M-H^{+}]^{-}$ $C_{10}H_{11}N_5O_4$ calcd 266.0895,

found 266.0866. Crystallographic and refinement parameters are shown in Supplementary Table 7. (Supplementary Fig. 71).

Supplementary Tables

				Ratio of oxzalidinone thione products				
2-Thiooxazole **4b** (M)	Glyceraldehyde **7** (M)	Yield (%)	**4b** remaining (%)	*arabino* **1a**	*ribo* **1a**	*xylo* **1a**	*p-lyxo* **1a**	*f-Lyxo* **1a**
0.25	0.5	51	36	35	35	12	11	7
0.50	0.5	35	44	37	36	10	12	5
0.50	1.0	59	25	35	36	12	11	6
0.25	1.0	74	8	36	35	10	12	7

Supplementary Table 1. *– Table showing yields of pentose oxazolidinone thiones (**1a**) and their relative diastereomeric ratio, across a variety of concentrations of **4b** and glyceraldehyde (**7**). All reactions were heated for 24 h at 60 ^{o}C.*

Anhydro-nucleoside	Method	*2',3'-cyclic phosphate*	*2',3'-cyclic-5'-bisphosphate*	*Total*
*8,2'-O-anhydro-9-β-arabinofuranosyl-cycloadenosine (**17A**)*	**I**	*22%*	*33%*	*55%*
	**II**	*24%*	*8%*	*32%*
	**III**	*38%*	*15%*	*53%*
	**IV**	*41%*	*16%*	*57%*
	**V**	*23%*	*11%*	*34%*
*8,2'-O-anhydro-9-β-arabinofuranosyl-cycloinosine (**17I**)*	**I**	*38%*	*32%*	*70%*
	**II**	*32%*	*11%*	*43%*
*ancitabine (**11**)*	**II**	*47%*	*18%*	*65%*
	**V**	*44%*	*19%*	*63%*

Supplementary table 2. *– Table showing yields of nucleoside phosphorylation following general methods **I** - **V**. Yields were calculated based on comparison with an internal standard (DSS).*

**Compound**	*arabino*-**1a**	*lyxo-furano*-**1a**	*lyxo-pyrano*-**1a**	*ribo*-**1a**
chemical formula	$C_6H_9NO_4S$	$C_{12}H_{18}N_2O_8S_2$	$C_6H_9NO_4S$	$C_6H_9NO_4S$
M_r/gmol^{-1}	191.20	382.40	191.20	191.20
crystal system	monoclinic	triclinic	orthorhombic	orthorhombic
space group	$P2_1$	$P1$	$P2_12_12_1$	$P2_12_12_1$
a/Å	5.08770(10)	5.73870(10)	7.7510(5)	8.2530(5)
b/Å	10.9482(2)	8.16100(10)	9.5566(6)	8.5043(5)
c/Å	7.04620(10)	8.6702(2)	10.5180(7)	11.1375(7)
α/°	90	100.2260(10)	90	90
β/°	91.9770(10)	98.3830(10)	90	90
γ/°	90	96.2460(10)	90	90
V/Å^3	392.248(12)	391.536(12)	779.10(9)	781.70(8)
Z	2	1	4	4
D_c/gcm^{-3}	1.619	1.622	1.630	1.625
F(000)	200	200	400	400
λ/Å	0.71073	0.71073	0.71073	0.71073
μ/mm^{-1}	0.386	0.386	0.388	0.387
T/K	100(2)	100(2)	100(2)	100(2)
crystal size/mm	0.20 × 0.18 × 0.14	0.28 × 0.24 × 0.18	0.28 × 0.24 × 0.20	0.20 × 0.16 × 0.12
index range	-6 → 6<br>-14 → 14<br>-9 → 9	-7 → 7<br>-11 → 11<br>-11 → 11	-10 → 10<br>-12 → 12<br>-14 → 14	-11 → 11<br>-11 → 11<br>-14 → 15
collected reflections	10729	14400	14918	8818
unique reflections	2023	4030	2018	2004
R_{int}	0.0214	0.0200	0.0328	0.0314
reflections with $I > 2\sigma(I)$	2009	3953	1981	1934
no. parameters	121	241	121	121
$R(F)$, $F > 2\sigma$(F)	0.0186	0.0208	0.0220	0.0263
$wR(F^2)$, $F > 2\sigma$(F)	0.0188	0.0213	0.0225	0.0276
$R(F)$, all data	0.0497	0.0531	0.0557	0.0655
$wR(F^2)$, all data	0.0498	0.0535	0.0562	0.0663
Δ_r (min., max.)/e Å^{-3}	-0.213, 0.231	-0.167, 0.292	-0.175, 0.305	-0.266, 0.277
CCDC deposition number	1522010	1522011	1522012	1522013

Supplementary Table 3. *Crystallographic and refinement parameters for arabino-**1a**, lyxo-furano-**1a**, lyxo-pyrano-**1a** and ribo-**1a**.*

Compound	*xylo*-**1a**	*arabino*-**1b**	**4b**	*arabino*-**12a**
chemical formula	$C_6H_9NO_4S$	$C_9H_{10}N_2O_4S$	C_3H_3NOS	$C_{13}H_{12}N_2O_5$
M_r/gmol^{-1}	191.20	242.25	101.12	276.25
crystal system	orthorhombic	triclinic	monoclinic	monoclinic
space group	$P2_12_12_1$	$P1$	$P2_1/c$	$C2$
a/Å	4.7529(2)	5.6835(2)	3.985(3)	21.1572(7)
b/Å	7.0665(2)	5.6922(2)	10.002(3)	8.1067(3)
c/Å	22.8605(7)	17.1686(5)	10.602(3)	6.8795(2)
α/°	90	94.661(2)	90	90
β/°	90	95.058(2)	95.398(4)	107.2490(10)
γ/°	90	109.588(2)	90	90
V/Å^3	767.80(5)	517.60(3)	420.7(3)	1126.87(6)
Z	4	2	4	4
D_c/gcm^{-3}	1.654	1.554	1.596	1.628
F(000)	400	252	208	576
λ/Å	0.71073	0.71073	0.71073	1.54178
μ/mm^{-1}	0.394	0.313	0.590	1.080
T/K	100(2)	100(2)	100(2)	100(2)
crystal size/mm	0.28 × 0.14 × 0.12	0.34 × 0.20 × 0.16	0.22 × 0.18 × 0.16	0.24 × 0.16 × 0.12
index range	-6 → 6<br>-9 → 9<br>-30 → 30	-7 → 7<br>-7 → 7<br>-23 → 23	-5 → 5<br>-13 → 13<br>-14 → 14	-24 → 24<br>-9 → 9<br>-8 → 8
collected reflections	11456	14693	7757	13377
unique reflections	2008	5322	1099	1835
R_{int}	0.0453	0.0305	0.0275	0.0330
reflections with $I > 2\sigma(I)$	1911	5030	1030	1831
no. parameters	121	308	59	190
$R(F)$, $F > 2\sigma$(F)	0.0272	0.0306	0.0233	0.0217
$wR(F^2)$, $F > 2\sigma$(F)	0.0291	0.0332	0.0248	0.0217
$R(F)$, all data	0.0671	0.0691	0.0618	0.0556
$wR(F^2)$, all data	0.0679	0.0704	0.0629	0.0556
Δ_r (min., max.)/e Å^{-3}	-0.181, 0.279	-0.190, 0.281	-0.304, 0.308	-0.200, 0.226
CCDC deposition number	1522014	1522015	1522016	1522017

Supplementary Table 4. *Crystallographic and refinement parameters for xylo-**1a,** arabino-**1b**, **4b** and arabino-**12a**.*

Compound	*ribo*-**12a**	**15**	**16b**	**D-16c**
chemical formula	$C_{13}H_{14}N_2O_6$	$C_6H_4N_2S$	$C_9H_{10}N_4O_4$	$C_9H_{12}N_4O_5$
M_r/gmol^{-1}	294.26	136.17	238.21	256.23
crystal system	orthorhombic	monoclinic	orthorhombic	orthorhombic
space group	$P2_12_12_1$	Pc	$P2_12_12_1$	$P2_12_12_1$
a/Å	6.6843(4)	3.8091(2)	6.36040(10)	5.16040(10)
b/Å	8.0162(4)	9.4498(5)	9.83730(10)	13.4735(2)
c/Å	24.7739(14)	9.2941(4)	16.07840(10)	14.8581(2)
α/°	90	90	90	90
β/°	90	100.940(4)	90	90
γ/°	90	90	90	90
V/Å^3	1327.45(13)	328.46(3)	1006.01(2)	1033.06(3)
Z	4	2	4	4
D_c/gcm^{-3}	1.472	1.377	1.573	1.647
F(000)	616	140	496	536
λ/Å	1.54178	1.54178	1.54184	1.54184
μ/mm^{-1}	1.009	3.567	1.081	1.172
T/K	100(2)	100(2)	150.0(1)	152(5)
crystal size/mm	0.28 × 0.16 × 0.10	0.20 × 0.18 × 0.08	0.23 × 0.14 × 0.11	0.26 × 0.08 × 0.03
index range	-6 → 7<br>-9 → 9<br>-29 → 29	-4 → 4<br>-11 → 11<br>-11 → 10	-7 → 7<br>-12 → 11<br>-20 → 20	-6 → 6<br>-16 → 16<br>-17 → 17
collected reflections	30166	5548	34327	33377
unique reflections	2274	1006	2022	1821
R_{int}	0.0771	0.0667	0.0340	0.0804
reflections with $I > 2\sigma(I)$	2168	921	2010	1771
no. parameters	206	82	164	177
$R(F)$, $F > 2\sigma$(F)	0.0546	0.0761	0.0279	0.0289
$wR(F^2)$, $F > 2\sigma$(F)	0.0558	0.0813	0.0280	0.0289
$R(F)$, all data	0.1495	0.1814	0.0730	0.0747
$wR(F^2)$, all data	0.1505	0.1872	0.0731	0.0754
Δ_r (min., max.)/e Å^{-3}	-0.250, 0.413	-0.293, 0.924	-0.215, 0.250	-0.195, 0.180
CCDC deposition number	1522018	1522019	1522020	1522026

Supplementary Table 5. *Crystallographic and refinement parameters for ribo-**12a**, **15**, **16b** and D-**16c**.*

Compound	L-16c	17A	17I	19
chemical formula	$C_9H_{12}N_4O_5$	$C_{10}H_{17}N_5O_7$	$C_{10}H_{10}N_4O_5$	$C_{10}H_{11}N_5O_4$
M_r/gmol^{-1}	256.23	319.28	266.22	265.24
crystal system	orthorhombic	orthorhombic	monoclinic	monoclinic
space group	$P2_12_12_1$	$P2_12_12_1$	$P2_1$	$P2_1$
a/Å	5.16330(10)	5.9577(2)	8.59170(10)	8.3830(3)
b/Å	13.4533(2)	8.6625(3)	6.88950(10)	6.73040(10)
c/Å	14.8581(2)	27.0257(8)	9.4985(2)	10.1823(3)
α/°	90	90	90	90
β/°	90	90	109.654(2)	112.672(4)
γ/°	90	90	90	90
V/Å^3	1032.09(3)	1394.76(8)	529.484(16)	530.10(3)
Z	4	4	2	2
D_c/gcm^{-3}	1.649	1.521	1.670	1.662
F(000)	536	672	276	276
λ/Å	1.5418	1.54184	1.54184	1.54184
μ/mm^{-1}	1.174	1.119	1.178	1.127
T/K	100(1)	200(1)	150(1)	150(1)
crystal size/mm	0.38 × 0.06 × 0.04	0.37 × 0.04 × 0.02	0.40 × 0.23 × 0.13	0.47 × 0.08 × 0.07
index range	-6 → 6<br>-16 → 16<br>-17 → 17	-5 → 7<br>-10 → 10<br>-32 → 28	-10 → 10<br>-8 → 8<br>-10 → 11	-9 → 9<br>-8 → 5<br>-11 → 12
collected reflections	14385	3241	6422	2041
unique reflections	1820	2132	1851	1393
R_{int}	0.0603	0.0186	0.0131	0.0181
reflections with $I > 2\sigma(I)$	1772	2022	1838	1374
no. parameters	177	225	178	182
$R(F)$, $F > 2\sigma$(F)	0.0320	0.0281	0.0245	0.0239
$wR(F^2)$, $F > 2\sigma$(F)	0.0330	0.0307	0.0248	0.0243
$R(F)$, all data	0.0835	0.0686	0.0645	0.0613
$wR(F^2)$, all data	0.0844	0.0703	0.0646	0.0619
Δ_r (min., max.)/e Å^{-3}	-0.204, 0.284	-0.182, 0.156	-0.183, 0.178	-0.176, 0.192
CCDC deposition number	1522024	1522025	1522023	1522021

Supplementary Table 6. *Crystallographic and refinement parameters for* L-***16c****,* ***17A****,* ***17I*** *and* ***19****.*

Compound	20
chemical formula	$C_{10}H_{11}N_5O_4$
M_r/gmol^{-1}	265.24
crystal system	orthorhombic
space group	$P2_12_12_1$
a/Å	6.9590(2)
b/Å	7.4942(2)
c/Å	20.5342(5)
α/°	90
β/°	90
γ/°	90
V/Å^3	1070.90(5)
Z	4
D_c/gcm^{-3}	1.645
F(000)	552
λ/Å	1.54184
μ/mm^{-1}	1.116
T/K	150(1)
crystal size/mm	0.13 × 0.12 × 0.09
index range	-8 → 5<br>-7 → 8<br>-19 → 24
collected reflections	2611
unique reflections	1707
R_{int}	0.0190
reflections with $I > 2\sigma(I)$	1631
no. parameters	182
$R(F)$, $F > 2\sigma$(F)	0.0292
$wR(F^2)$, $F > 2\sigma$(F)	0.0311
$R(F)$, all data	0.0712
$wR(F^2)$, all data	0.0724
Δ_r (min., max.)/e Å^{-3}	-0.215, 0.176
CCDC deposition number	1522022

Supplementary Table 7. *Crystallographic and refinement parameters for* ***20****.*

Supplementary Figures

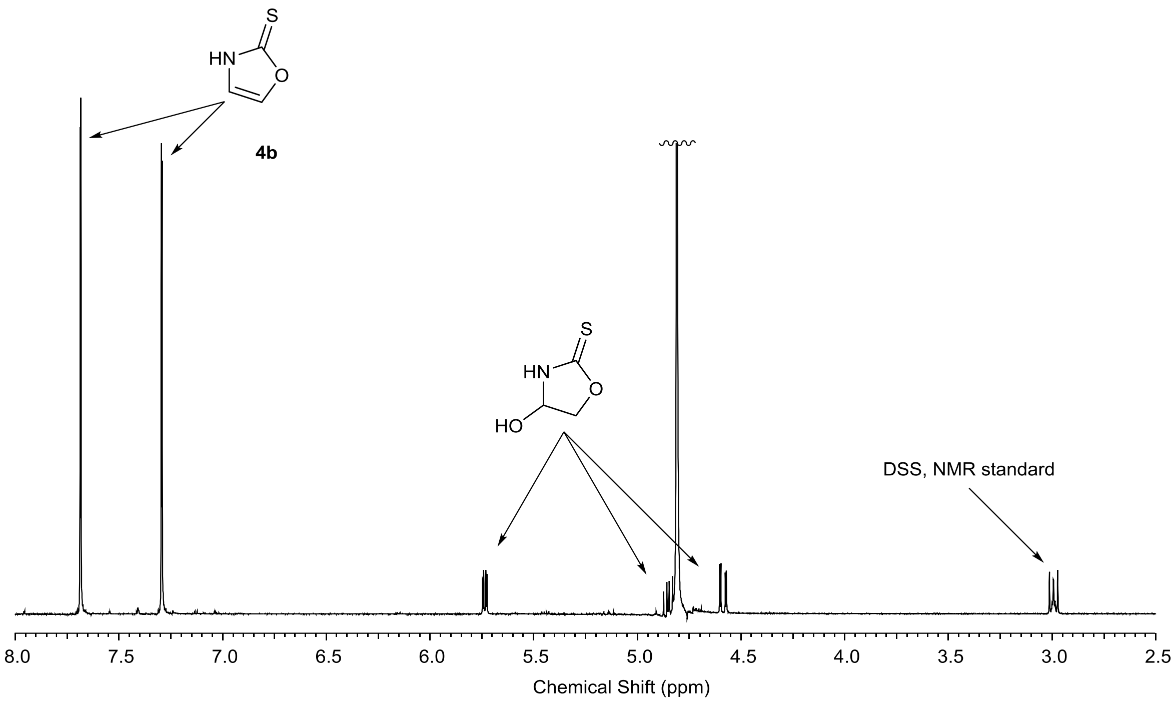

Supplementary Fig. 1. *– ^{1}H NMR (600 MHz, D_2O, 2.5 – 8.0 ppm) to show the reaction of glycolaldehyde* ***5*** *(100mM), thiocyanic acid (500mM) and 4,4-dimethyl-4-silapentane-1-sulfonic acid (DSS, NMR standard) in D_2O (1 mL) after incubation at 80 °C for 24 h. Calibration to internal standard indicates an 85% yield of 2-thiooxazole (**4b**) and a 15% yield of its hydrate, 4-hydroxyoxazolidine-2-thione.*

.

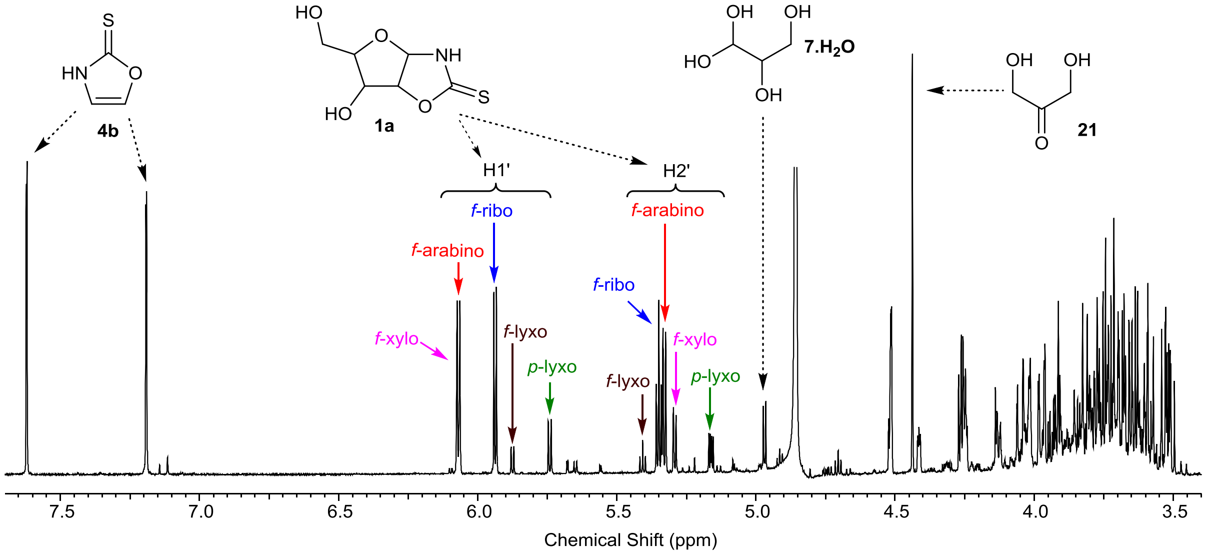

Supplementary Fig. 2. *– ^{1}H NMR spectrum (600 MHz, D_2O, 3.4 – 7.7 ppm) showing reaction of 2-thiooxazole (**4b**) with rac-glyceraldehyde (rac-**7**). 2-Thiooxazole (**4b**; 500mM) and glyceraldehyde (**7**; 1M) at pD 7 were incubated at 60 °C for 24 h.*

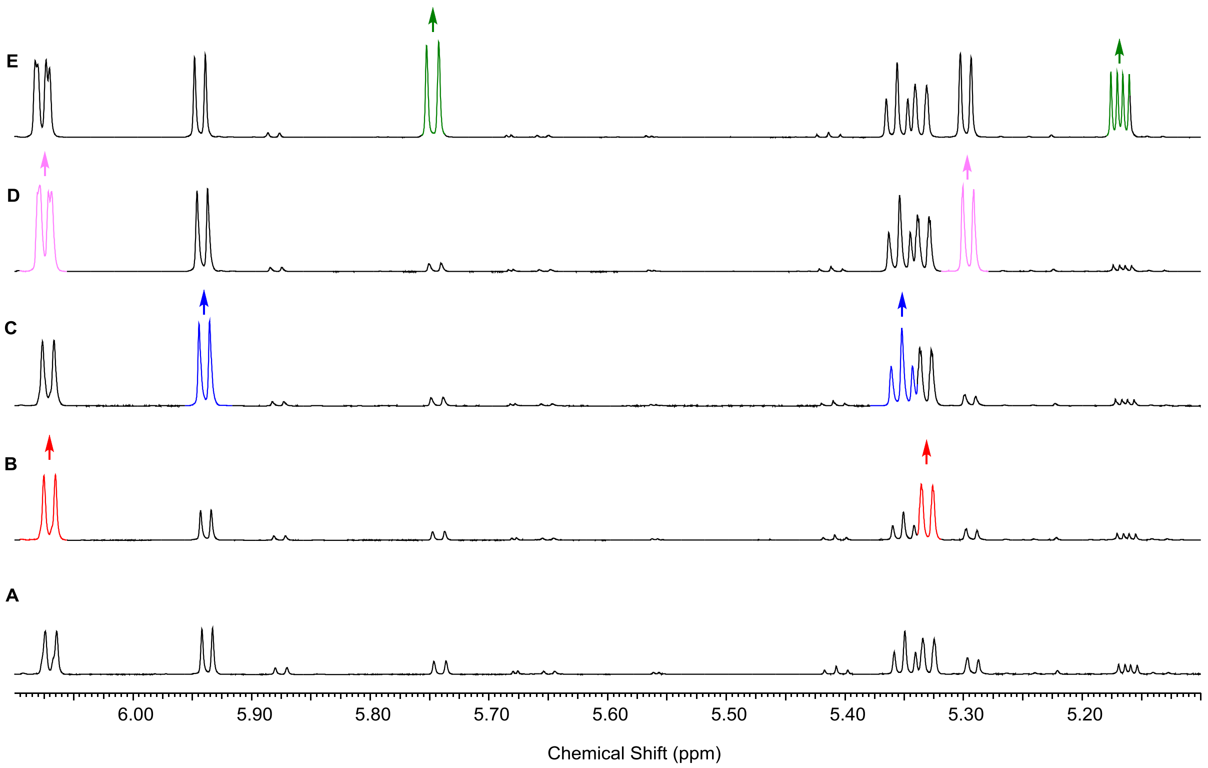

Supplementary Fig. 3. *–^{1}H NMR spectra (600 MHz, D_2O, 5.1 – 6.1 ppm) showing reaction of 2-thiooxazole (**4b**) with rac-glyceraldehyde (**7**). A – **4b** (0.5*M*) and **7** (1*M*) at pD 7, incubated at 60 °C for 24 h. B – spiked with arabino-thione arabino-**1a**. C – spiked with ribo-thione ribo-**1a**. D – spiked with xylo-thione xylo-**1a**. E – spiked with p-lyxo-thione p-lyxo-**1a**.*

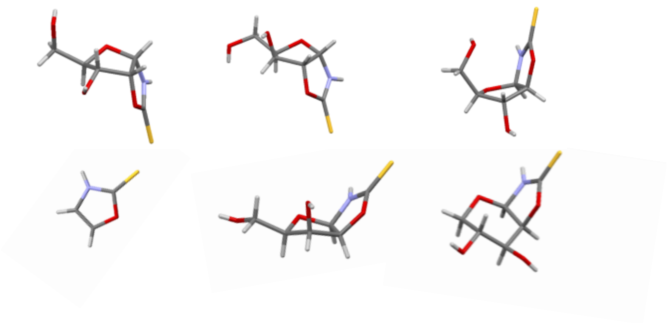

Supplementary Fig. 4. *Single crystal X-ray structures of D-pentose oxazolidinone thiones **1a** (D-ribo-**1a**, D-xylo-**1a**, D-arabino-**1a**, D-f-lyxo-**1a**, and D-p-lyxo-**1a**) and 2-thiooxazole (**4b**) (clockwise from top left).*

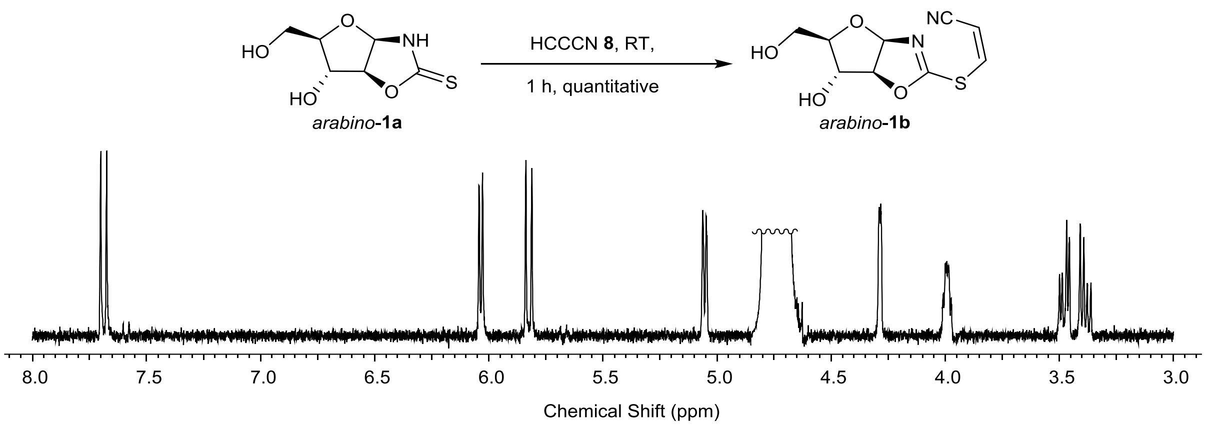

Supplementary Fig. 5. *- ^{1}H NMR spectrum (600 MHz, D_2O, 3.0 – 8.0 ppm) of (S-Z-cyanovinyl)-arabinofuranosyl oxazolidinone thione (arabino-**1b**). A solution arabinofuranosyl oxazolidinone thione (arabino-**1a**; 655mM) and cyanoacetylene (**8**; 1M) was incubated for 1 h, lyophillised and dissolved in D_2O before NMR spectra were acquired.*

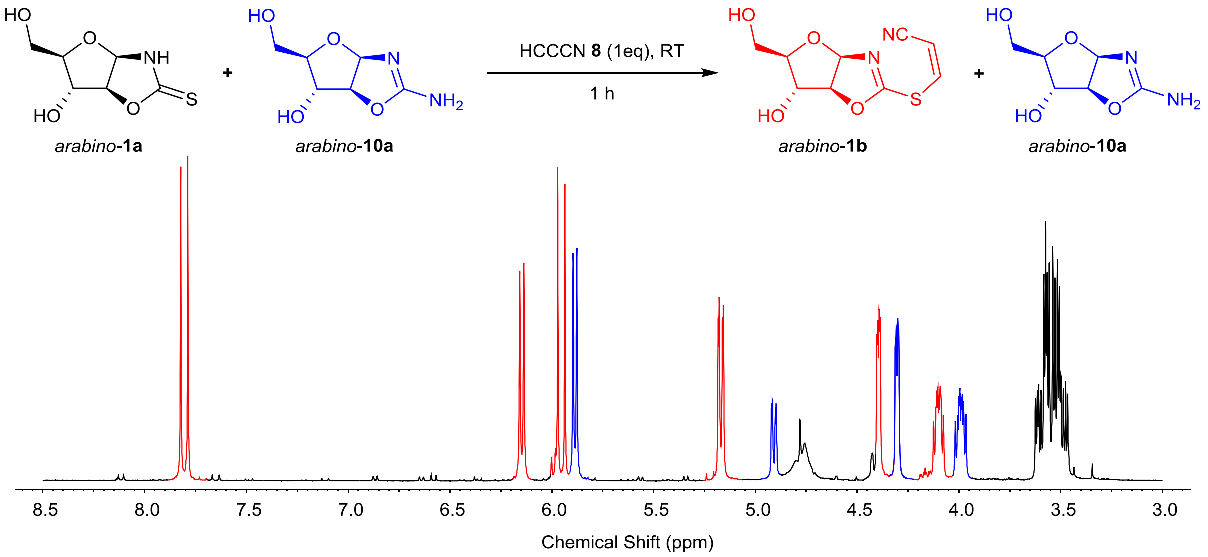

Supplementary Fig. 6. *- ^{1}H NMR spectrum (600 MHz, H_2O/D_2O, 3.0 – 8.0 ppm) of 1:1 mixture of arabinofuranosyl oxazolidinone thione (arabino-**1a**) and* arabinofuranosyl aminooxazoline *(arabino-**10a**) incubated with cyanoacetylene (**8**) showing selective cyanovinylation of arabino-**1a** to give (S-Z-cyanovinyl)-arabinofuranosyl oxazolidinone thione (arabino-**1b**) in the presence of arabino-**10a**. An aqueous solution of arabino-**1a** (240mM), arabino-**10a** (240mM) and cyanoacetylene (**8**; 250mM) was incubated at pH 7 for 1 h before NMR spectra were acquired.*

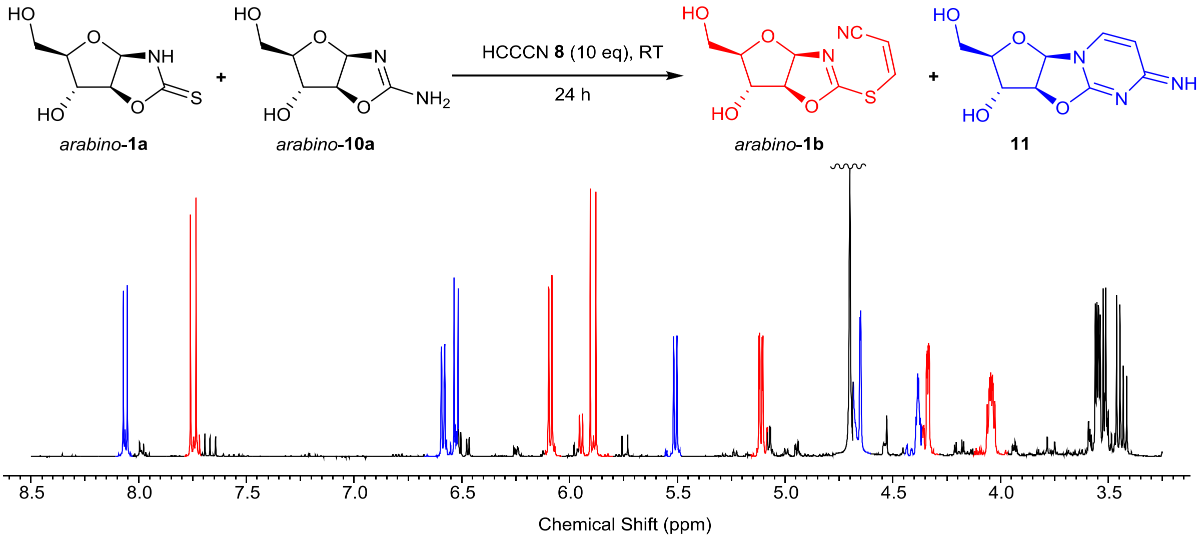

Supplementary Fig. 7. *- ^{1}H NMR spectrum (600 MHz, H_2O/D_2O, 3.25 – 8.5 ppm) to show the products of incubating a 1:1 mixture of arabinofuranosyl oxazolidinone thione arabino-**1a** and arabinofuranosyl aminooxazoline (arabino-**10a**) with cyanoacetylene (**8**; 10 eq.). An aqueous solution of arabino-**1a** (40 mM), arabino-**10a** (40 mM) and cyanoacetylene (**8**; 420 mM) was incubated at pH 7 for 24 h, leading to co-cyanovinylation of arabino-**1a** and arabino-**10a** to yield (S-Z-cyanovinyl)-arabinofuranosyl oxazolidinone thione (arabino-**1b**) and ancitabine (**11**), respectively.*

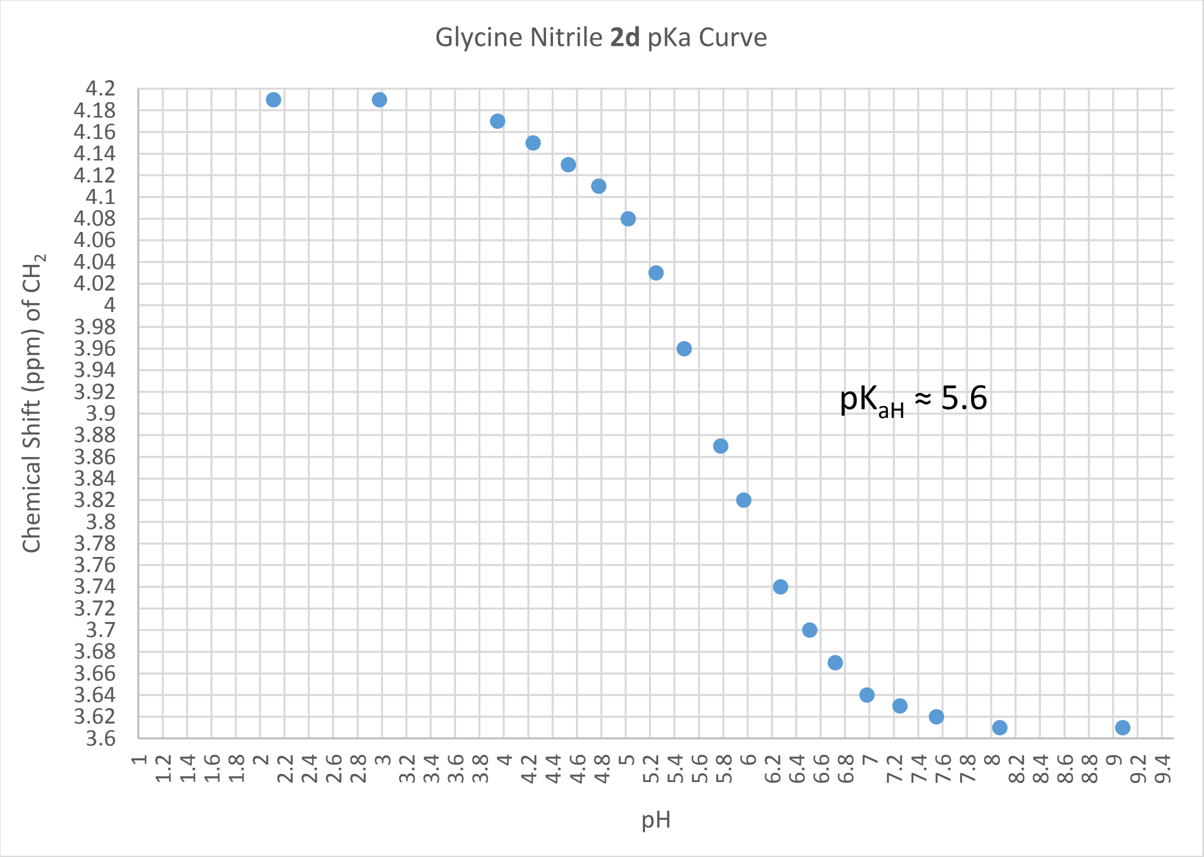

Supplementary Fig. 8. *– Spectrographic pK_a titration curve of glycine nitrile **2d**. pK_{aH} ($NCCH_2NH_3^+ \rightarrow NCCH_2NH_2$) was determined to be approximately 5.6 which agrees with literature value[9].*

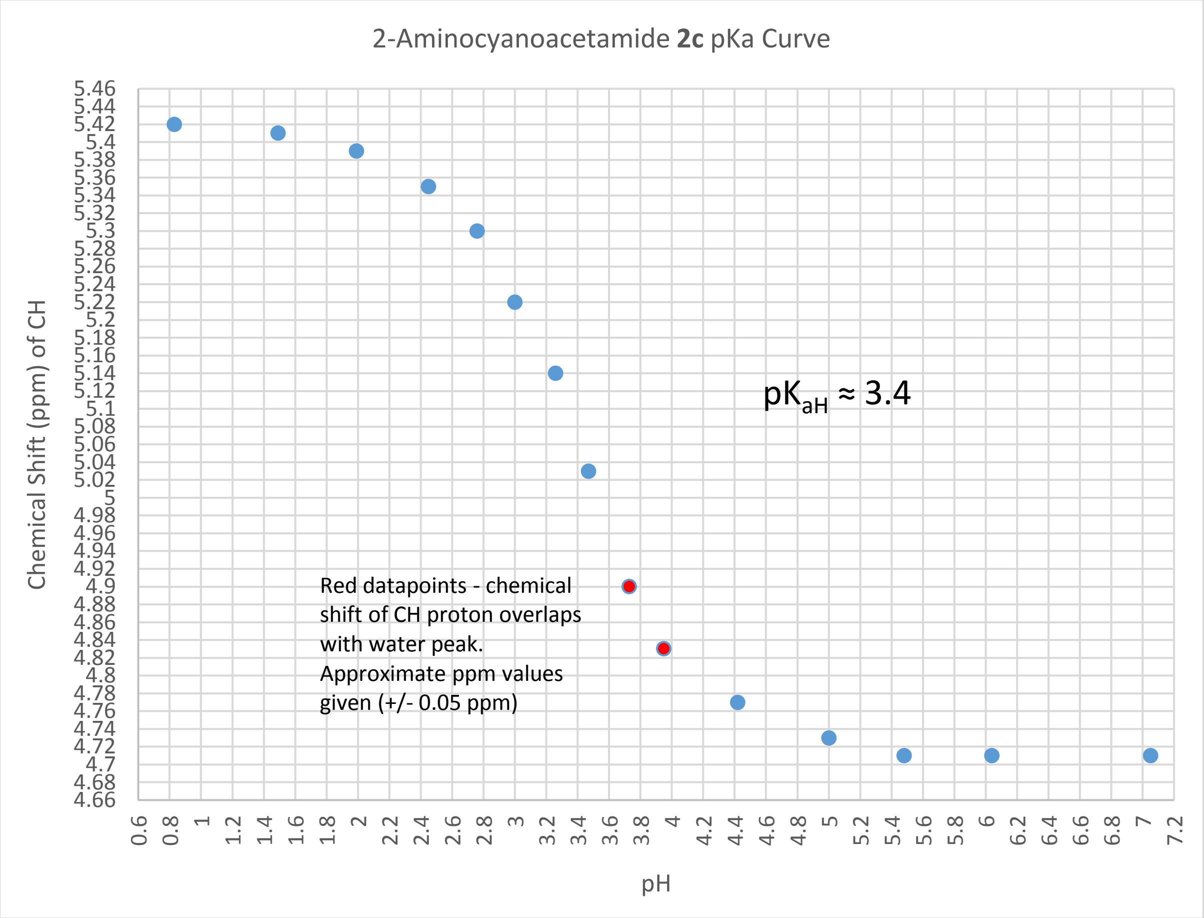

Supplementary Fig. 9. *– Spectrographic pK$_a$ titration curve of 2-aminocyanoacetamide **2c**. pK$_{aH}$ (NC(CONH$_2$)CHNH$_3^+$ → NC(CONH$_2$)CHNH$_2$) was determined to be approximately 3.4.*

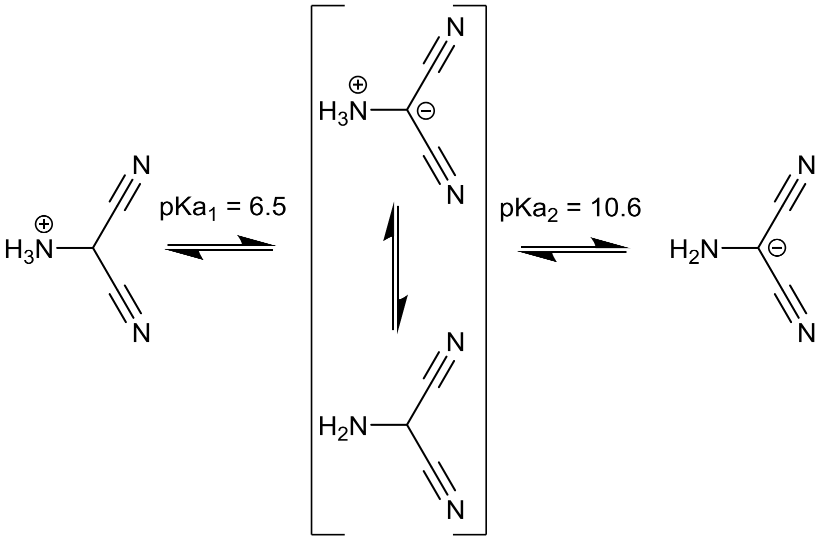

Supplementary Fig. 10. *– pK$_a$ values of HCN trimer **2b** are reported by Raulin et al.*[10]

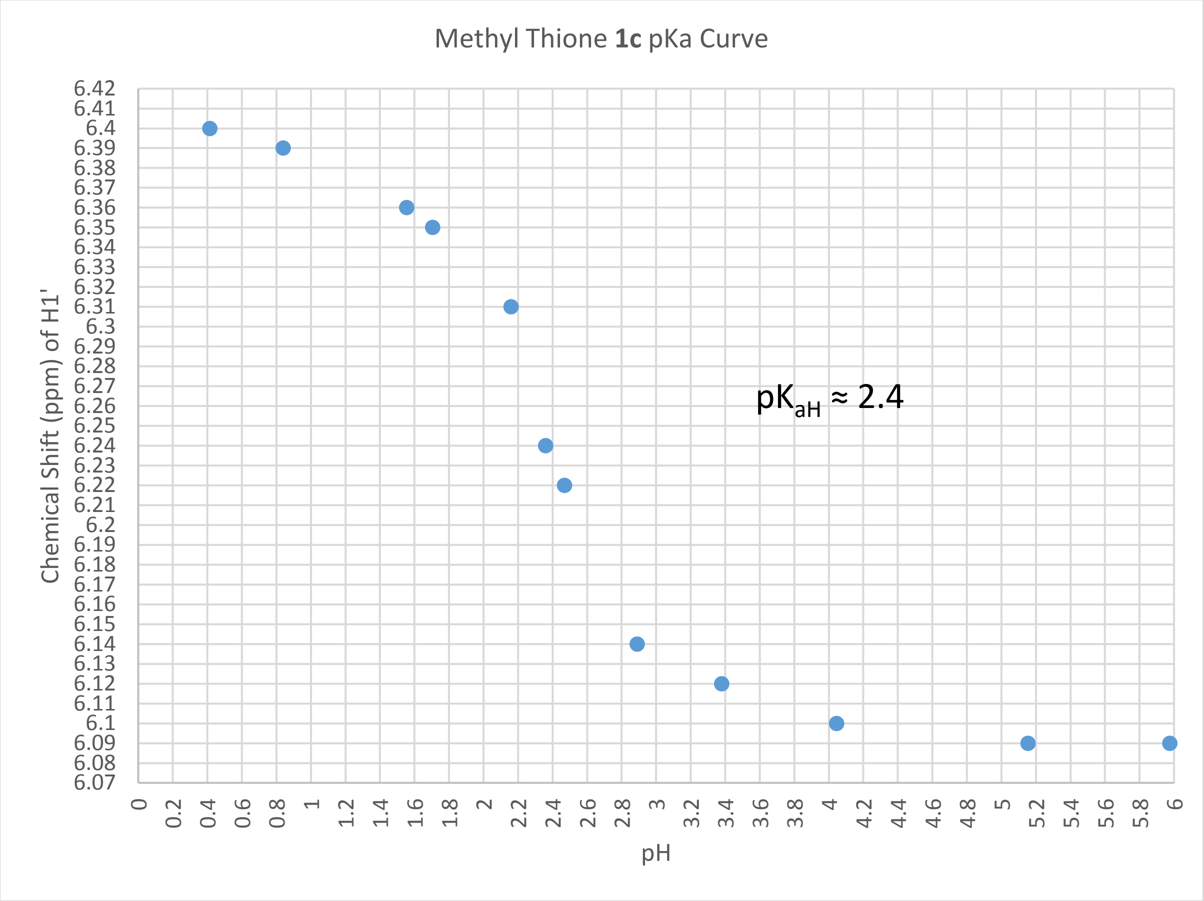

Supplementary Fig. 11. *– Spectrographic pK$_a$ titration curve of arabinofuranosyl-(2-thiomethyl)-oxazolidinone (**1c**). pK$_{aH}$ (N1 protonation) was determined to be approximately 2.4.*

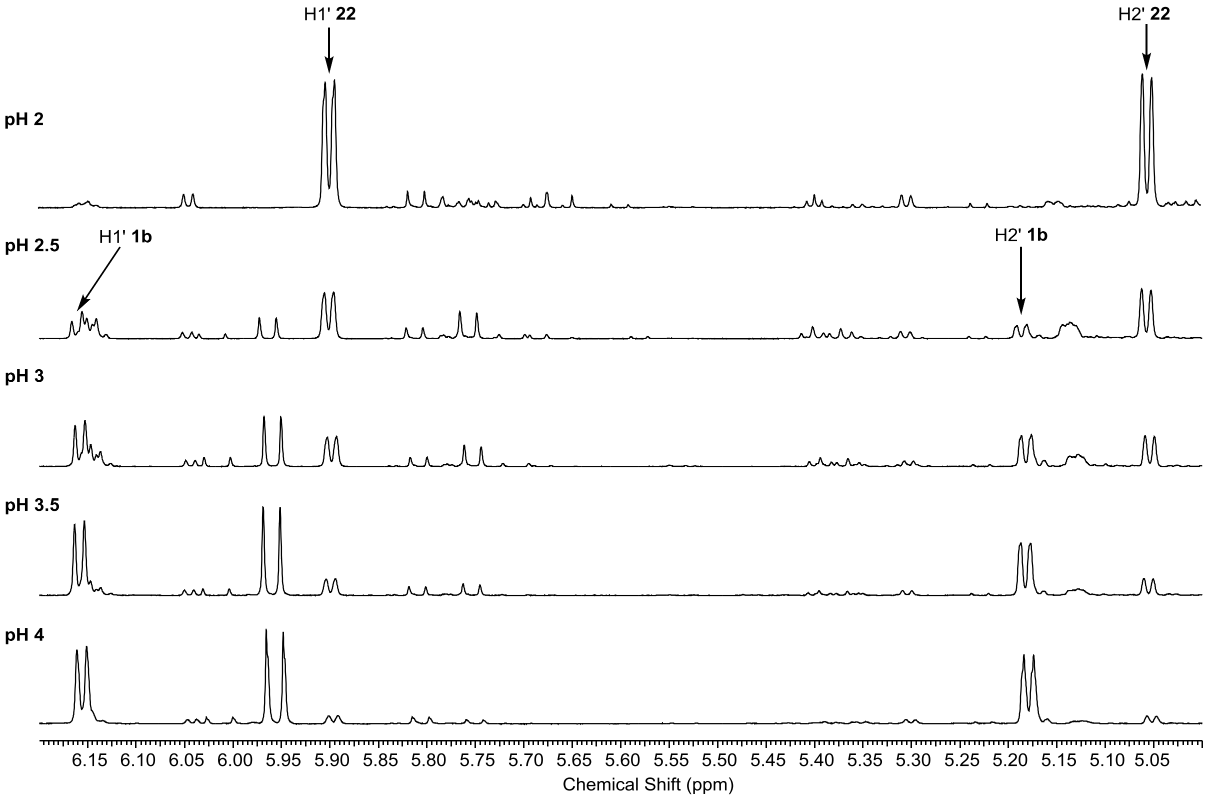

Supplementary Fig. 12. *– ^{1}H NMR spectra (600 MHz, H_2O/D_2O, 5.0 – 6.20 ppm) of (S-Z-cyanovinyl)-arabinofuranosyl oxazolidinone thione (arabino-**1b**) at pH's 2.5 – 4. Significant hydrolysis of arabino-**1b** occurs below pH 3.5 without any change in chemical shift of H1'. At pH 2 complete hydrolysis to arabinofuranosyl oxazolidinone (arabino-**22**) is observed within 15 min. No H1' downfield-shift was observed between pH4 and pH2.5, which suggests the pK_{aH} of arabino-**1b** is significantly lower than 2.*

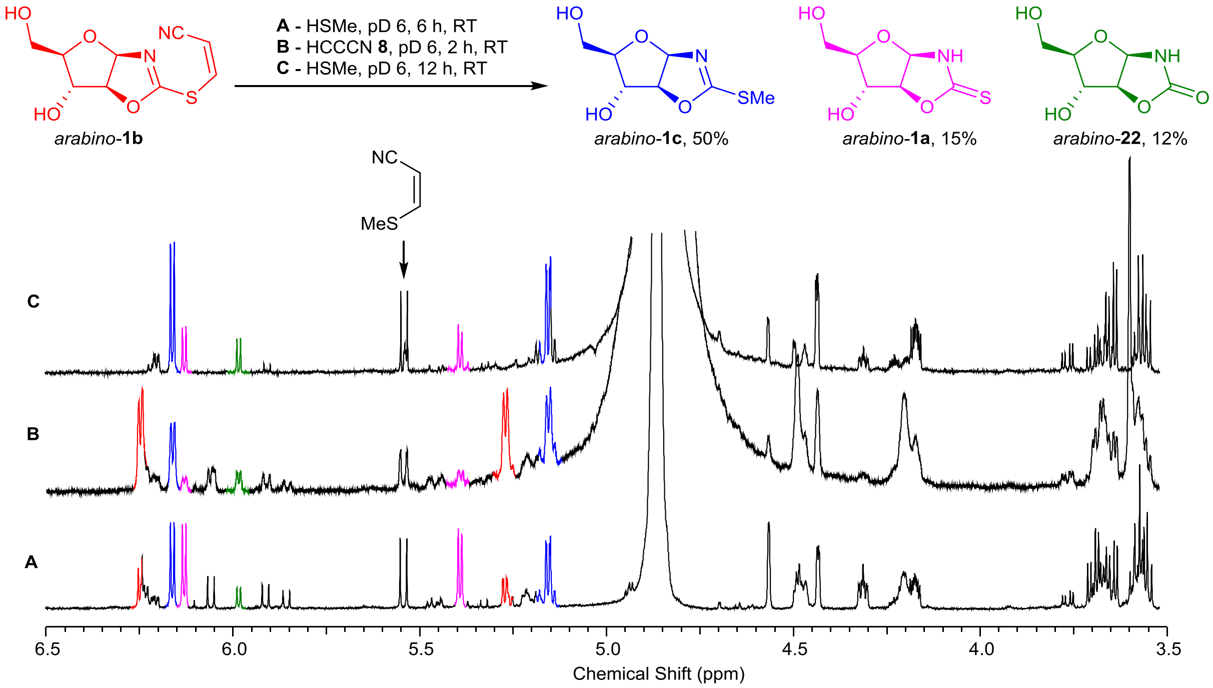

Supplementary Fig. 13. *- ^{1}H NMR spectra (600 MHz, D_2O, 3.5 – 6.5 ppm) showing conversion of (S-Z-cyanovinyl)-arabinofuranosyl oxazolidinone thione (arabino-**1b**) to* arabinofuranosyl-(2-thiomethyl)-oxazolidinone *(arabino-**1c**). An aqueous solution of arabino-**1b** at pD 6 was treated with methanethiol for 6 h. A – NMR to show conversion at 6 h, mixture contained 30% arabino-**1c**, 30% arabinofuranosyl oxazolidinone thione (arabino-**1a**), 10% arabino-**1b** and 10% arabinofuranosyl oxazolidinone (arabino-**22**). The solution was briefly sparged with nitrogen, an aqueous solution of cyanoacetylene (**8**) was added and the mixture was left to stand for 2 h. B – NMR to show near complete conversion of arabino-**1a** to arabino-**1b**; no significant change in amount of arabino-**1c** and arabino-**22** was observed. The solution was again saturated with methanethiol and incubated for 16 h. C - NMR to show a mixture containing 50% arabino-**1c**, 15% arabino-**1a** and 12% arabino-**22** after 16 h incubation with methanethiol.*

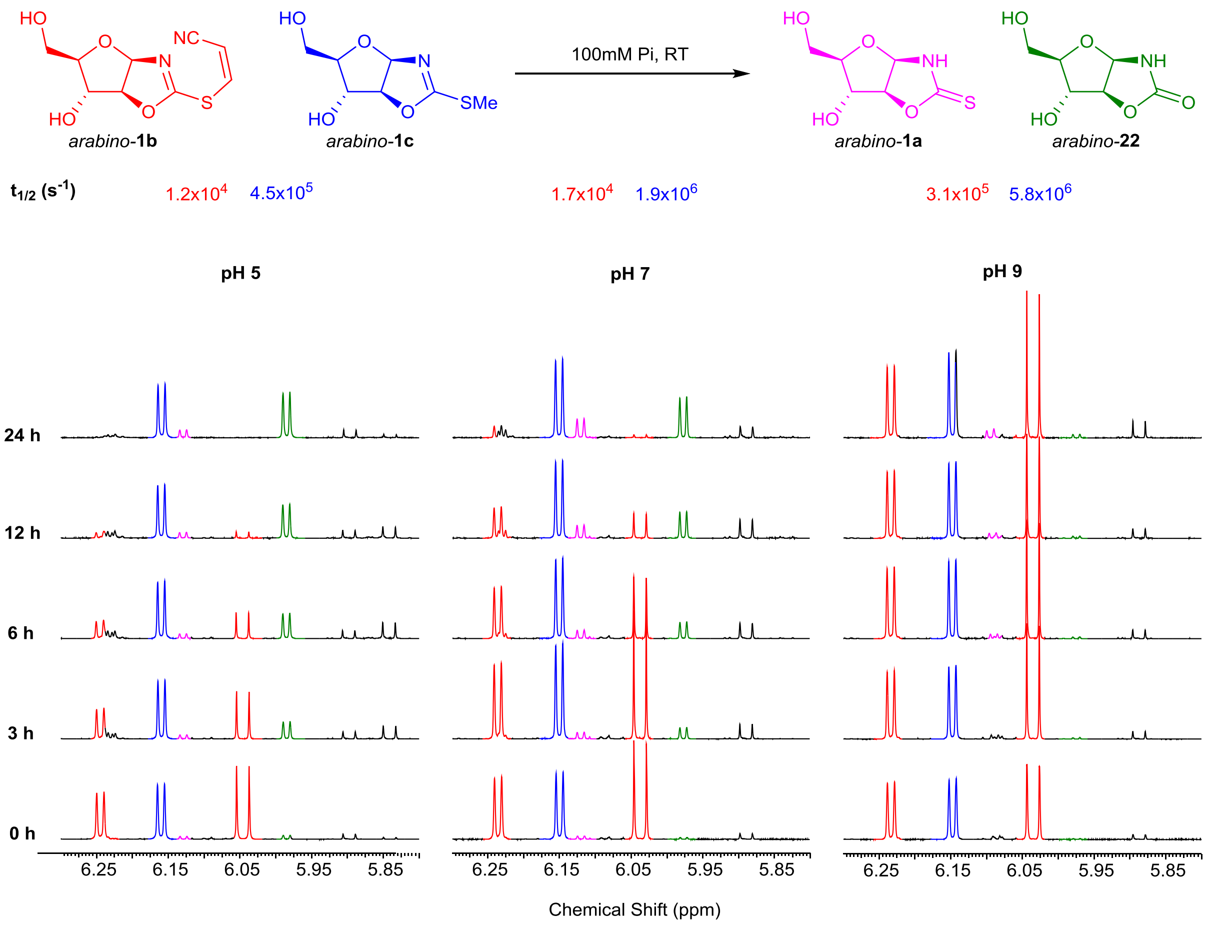

Supplementary Fig. 14. *– ^{1}H NMR (600 MHz, D_2O, 5.8 – 6.3 ppm) showing hydrolysis of S-Z-cyanovinyl-arabinofuranosyl oxazolidinone thione (arabino-**1b**) and* arabinofuranosyl-(2-thiomethyl)-oxazolidinone *(arabino-**1c**) across a range of pHs. Solutions of arabino-**1b** (50mM), arabino-**1c** (50mM) and Pi (100mM) at pHs 5, 7 and 9 were incubated at room temperature and spectra were periodically taken over 24 h.*

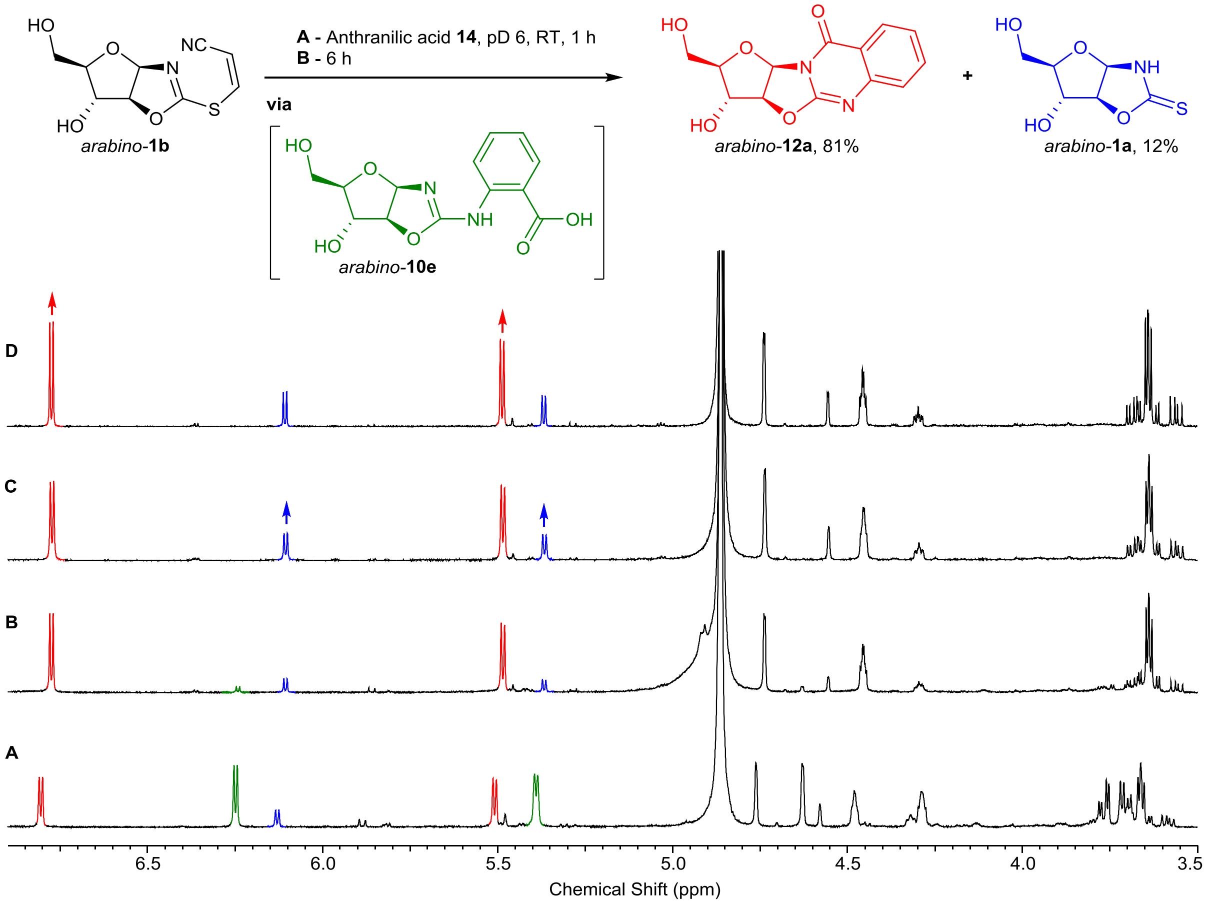

Supplementary Fig. 15. - *^{1}H NMR spectra (600 MHz, D_2O, 3.5 – 6.9 ppm) showing reaction of (S-Z-cyanovinyl)-arabinofuranosyl oxazolidinone thione (arabino-**1b**) with anthranilic acid (**14**). A - arabino-**1b** (250mM) and **14** (500mM) in D_2O (500 µL) at pD 6, incubated at RT for 1 h. B – after 6 h, calibration to an internal standard (DSS) gave yields of 81%* N3-arabinofuranosyl-2,2'-anhydroquinazolinedione *(arabino-**12a**) and 12% arabinofuranosyl oxazolidinone thione (arabino-**1a**). C - spiked with arabino-**1a**. D – spiked with arabino-**12a**.*

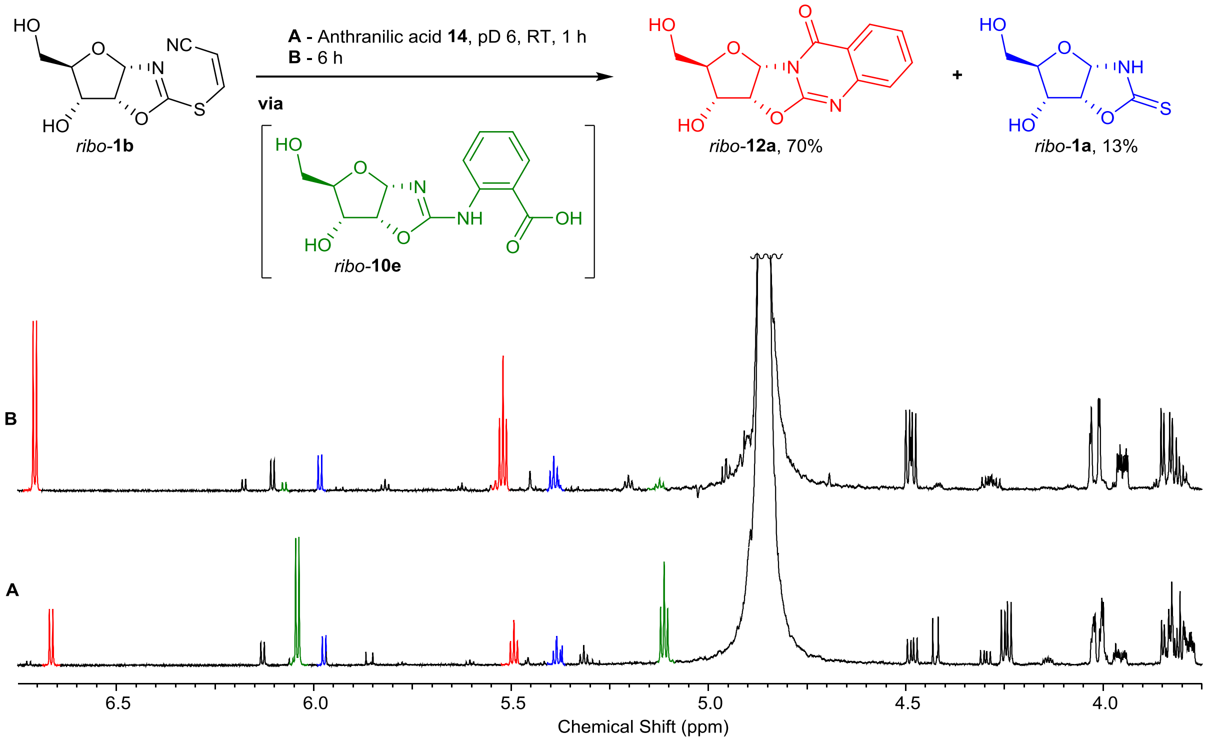

Supplementary Fig. 16. *- ^{1}H NMR spectra (600 MHz, D_2O, 3.75 – 6.75 ppm) showing reaction of (S-Z-cyanovinyl)-ribofuranosyl oxazolidinone thione (ribo-**1d**) with anthranilic acid (**14**). A – ribo-**1b** (250mM) and **14** (500mM) in D_2O (500 µL) at pD 6, incubated at RT for 1 h. B – after 6 h, calibration to an internal standard (DSS) gave yields of 70%* N3-ribofuranosyl-2,2'-anhydroquinazolinedione *(ribo-**12a**) and 13% ribofuranosyl oxazolidinone thione (ribo-**1a**).*

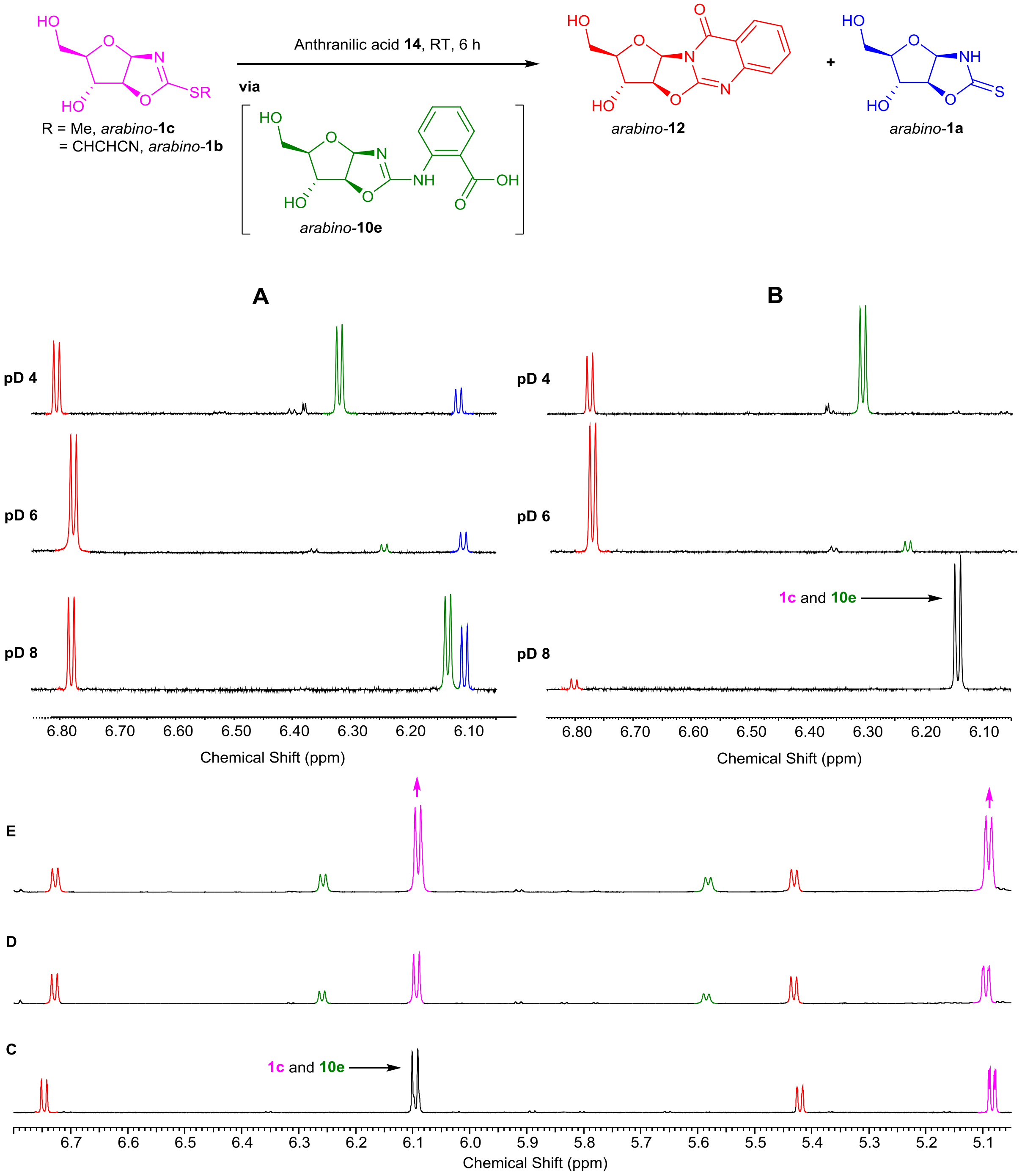

Supplementary Fig. 17. *- ^{1}H NMR spectra (600 MHz, D_2O, 6.05 – 6.85 ppm) showing reaction of (S-Z-cyanovinyl)-arabinofuranosyl oxazolidinone thione (arabino-**1b**) and* arabinofuranosyl-(2-thiomethyl)-oxazolidinone *(arabino-**1c**) with anthranilic acid (**14**). arabino-**1b** (250mM, column A) or arabino-**1c** (250mM, column B) was incubated with **14** (500mM) at RT for 6 h at pD 4, 6 or 8. arabino-**1b** reacts efficiently with anthranilic acid across pD 4-8, however arabino-**1c** only readily reacts at pD 4-6, it is also observed that quinazolinedione cyclisation (green → red) is significantly more rapid at pH's close to the pKa of aminooxazolines. C – Expanded ppm range (600 MHz, D_2O, 5.05 – 6.80) spectrum showing reaction of* arabinofuranosyl-(2-thiomethyl)-oxazolidinone *(arabino-**1c**) with anthranilic acid (**14**) at pD 8 after 48 h. D – Spectrum C adjusted to pD 4.5. E – Spectrum D spiked with arabino-**1c.***

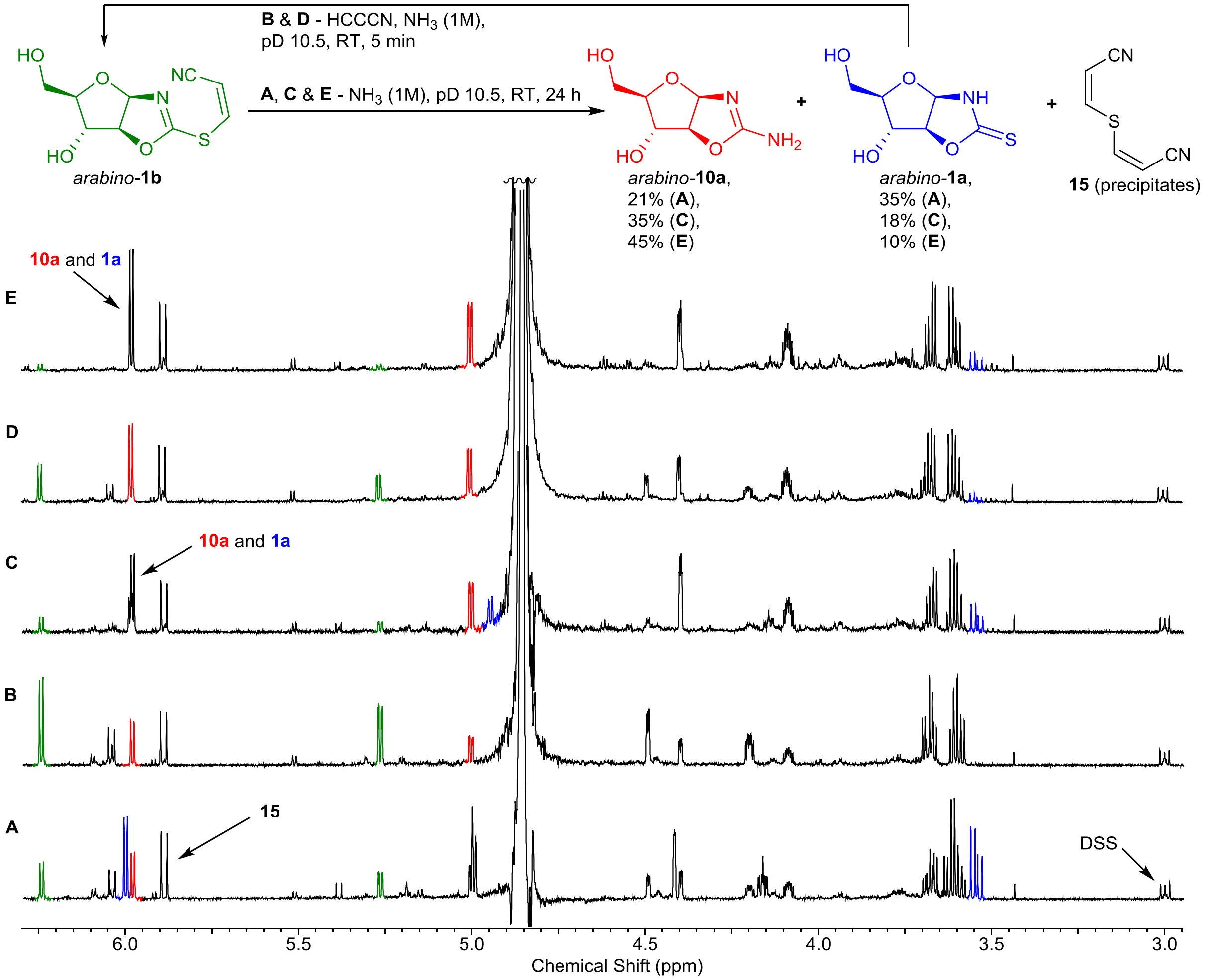

***Supplementary Fig. 18.** – ^{1}H NMR spectra (600 MHz, 2.95 – 6.3 ppm) showing reaction of (S-Z-cyanovinyl)-arabinofuranosyl oxazolidinone thione (arabino-**1b**) with ammonia (**2a**). A - arabino-**1b** (250 mM) and **2a** (1M) were incubated at pD 10.5 at RT for 24 h. B - arabinofuranosyl oxazolidinone thione (arabino-**1a**) was quantitatively converted back to arabino-**1b** by addition of cyanoacetylene (**8**). C – after a further 24 h at pD 10.5 at RT. D – after a 2nd recyanovinylation by addition of **8**. E - After another 24 h at pD 10.5 at RT.*

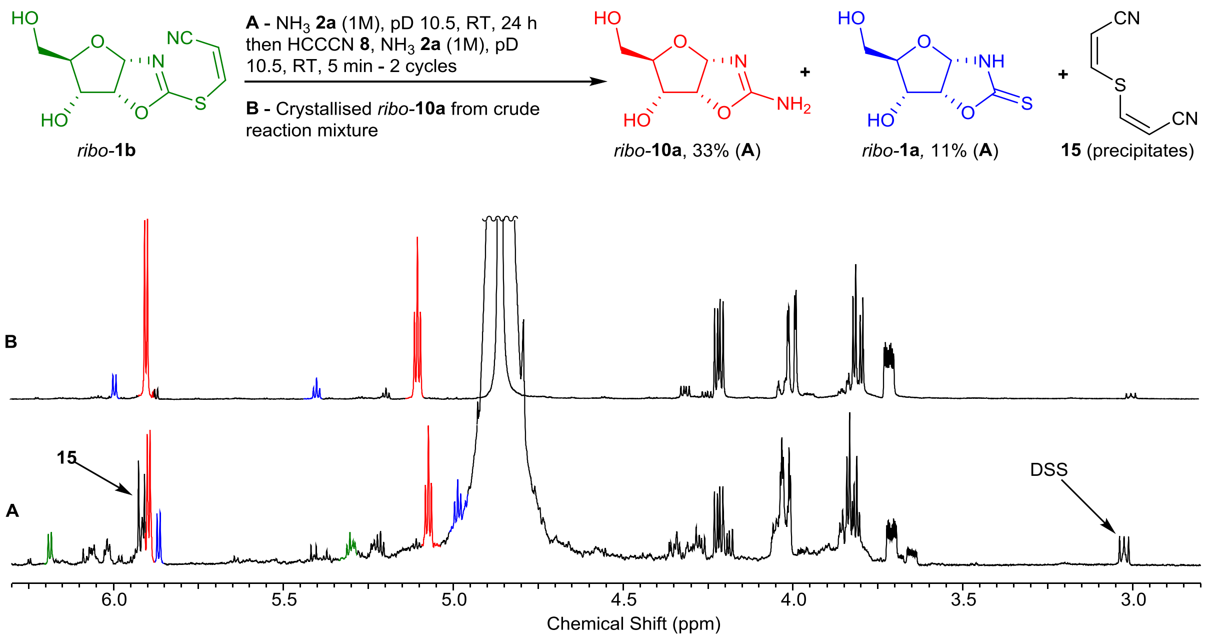

Supplementary Fig. 19. *– ^{1}H NMR spectra (600 MHz, 2.95 – 6.3 ppm) showing reaction of (S-Z-cyanovinyl)-ribofuranosyl oxazolidinone thione (ribo-**1b**) with ammonia (**2a**). A - ribo-**1b** (250 mM) and **2a** (1M) were incubated at pD 10.5 at RT for 24 h and the mixture was then treated with cyanoacetylene (**8**) at pD 10.5 at RT for 5 min. This cycle was repeated and after the 2nd treatment with **8** the reaction mixture was incubated for a further 24 h at pD10.5 at RT. B – crystals of ribo-**10a** obtained from the crude reaction mixture.*

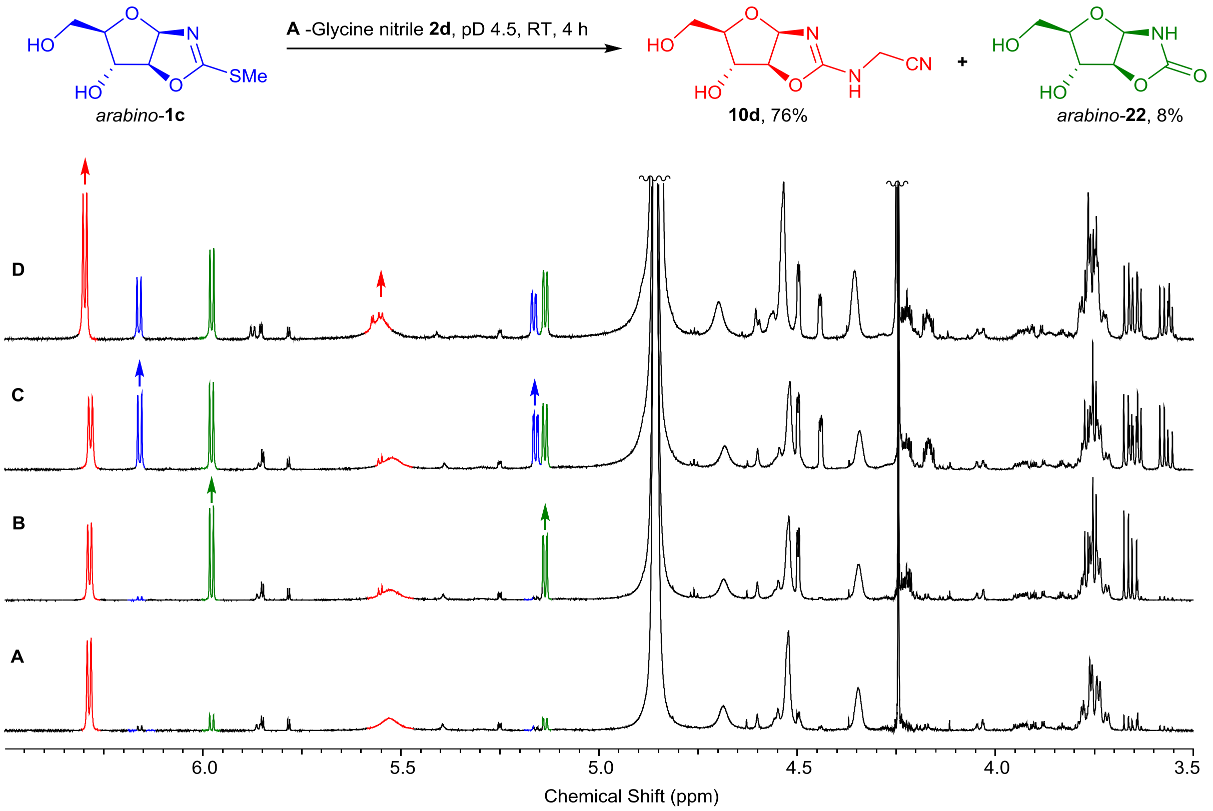

Supplementary Fig. 20. *- ^{1}H NMR spectra (600 MHz, D_2O, 3.5 – 7.0 ppm) showing reaction of arabinofuranosyl-(2-thiomethyl)-oxazolidinone (arabino-**1c**) with glycine nitrile (**2d**). A - arabino-**1c** (250mM) and **2d** (500mM) in D_2O (500 µL) at pD 4.5, incubated at RT for 4 h. Calibration to an internal standard (DSS) gave yields of 76% arabinofuranosyl-N-acetonitrile-aminooxazoline (**10d**) and 8% arabinofuranosyl oxazolidinone (arabino-**22**). B – spiked with arabino-**22**. C - spiked with arabino-**1c**. D – spiked with **10d**.*

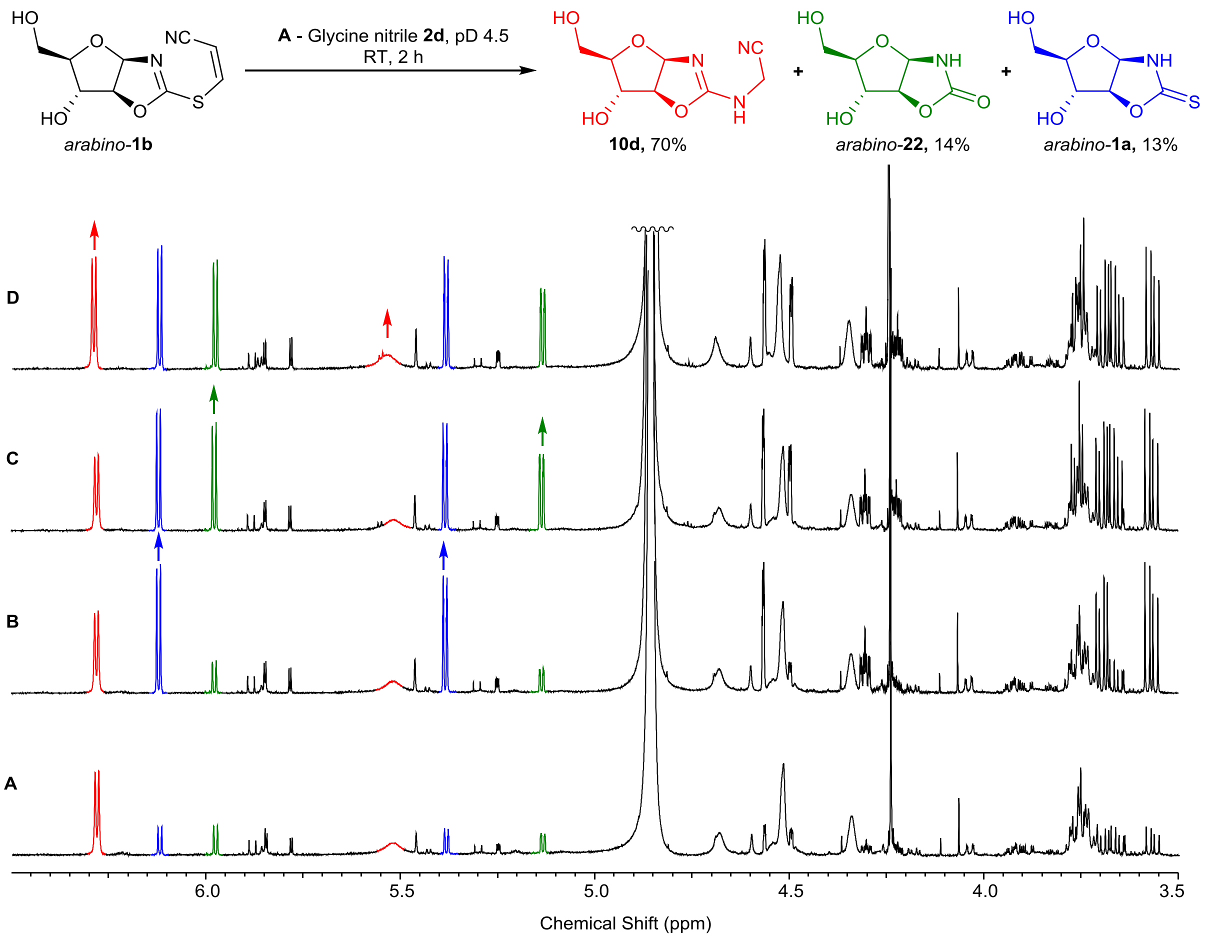

Supplementary Fig. 21. *- ^{1}H NMR spectra (600 MHz, D_2O, 3.5 – 7.0 ppm) showing reaction of (S-Z-cyanovinyl)-arabinofuranosyl oxazolidinone thione arabino-**1b** with glycine nitrile **2d**. A - arabino-**1b** (250mM) and **2d** (500mM) in D_2O (500 µL) at pD 4.5, incubated at RT for 2 h. Calibration to an internal standard (DSS) gave yields of 70% arabinofuranosyl-N-acetonitrile-aminooxazoline (**10d**), 13% arabinofuranosyl oxazolidinone thione (arabino-**1a**) and 14% arabinofuranosyl oxazolidinone (arabino-**22**). B – spiked with arabino-**1a**. C – spiked with arabino-**22**. D – spiked with **10d**.*

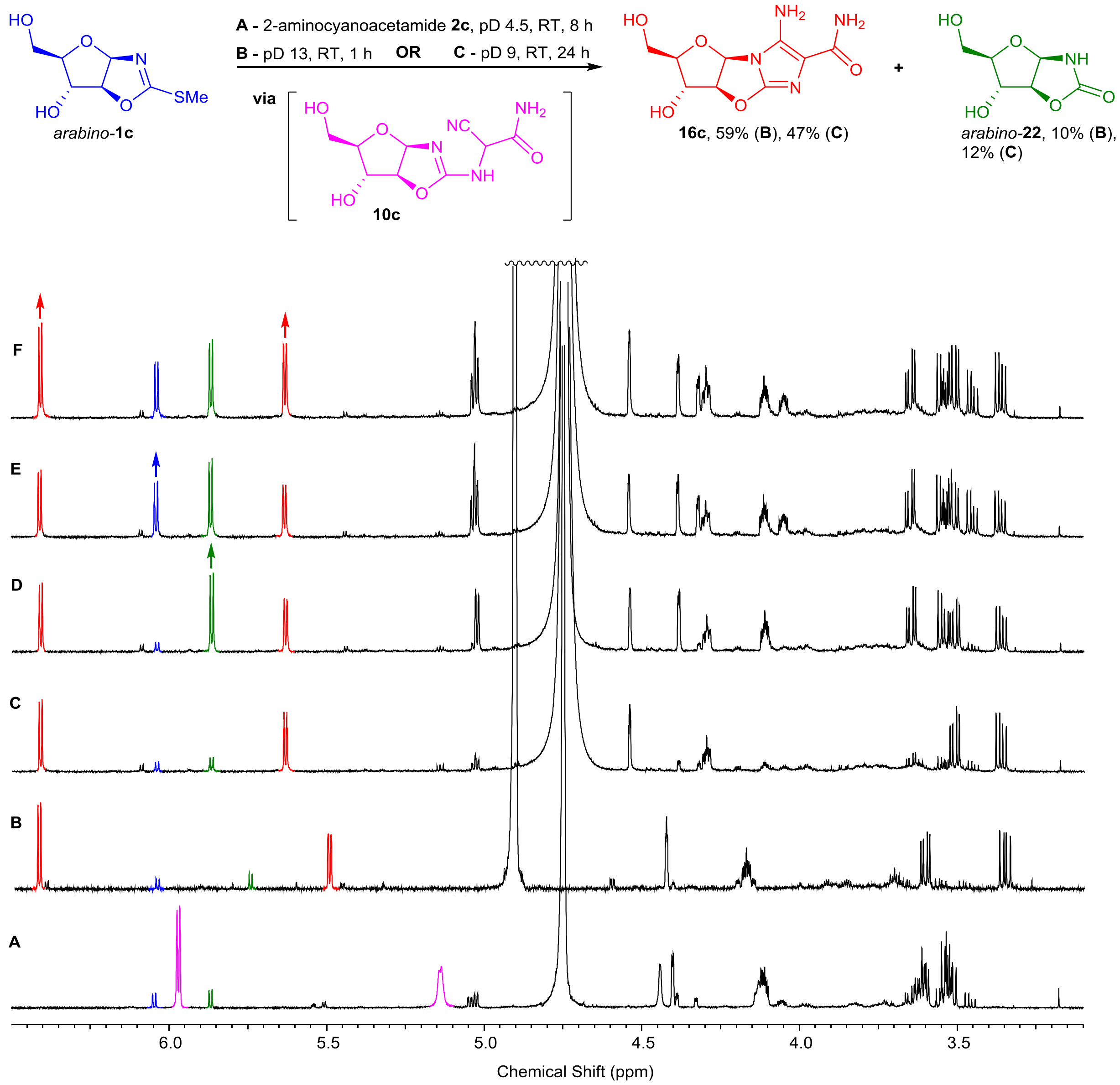

Supplementary Fig. 22. *-* ^{1}*H NMR spectra (600 MHz,* D_2O*, 3.1 – 7.0 ppm) showing reaction of arabinofuranosyl-(2-thiomethyl)-oxazolidinone (arabino-**1c**) with 2-aminocyanoacetamide (**2c**). A – arabino-**1c** (250mM) and **2c** (500mM) in* D_2O *(500 µL) at pD 4.5 incubated at RT for 8 h. Calibration to an internal standard gave a yield of 81% (aminooxazoline **10c**, 5.96 ppm). B - aliquot (50 µL) of A incubated at pD 13 at RT for 1 h giving a yield of 59%* 2,2'-anhydro-5-aminoimidazole-4-carboxamide-β-furanosylarabinoside *(**16c**). C - aliquot (50 µL) of A was added to ammonium hydroxide in* D_2O *(450 µL, 100 mM), the solution was adjusted to pD 9 with NaOD (4M) and incubated for 24 h at RT. Calibration to an internal standard gave yields of 47% **16c**, 12% arabinofuranosyl oxazolidinone (arabino-**22**) with 7% arabino-**1c** remaining. D – spiked with arabino-**22**. E – spiked with arabino-**1c**. F – spiked with **16c**.*

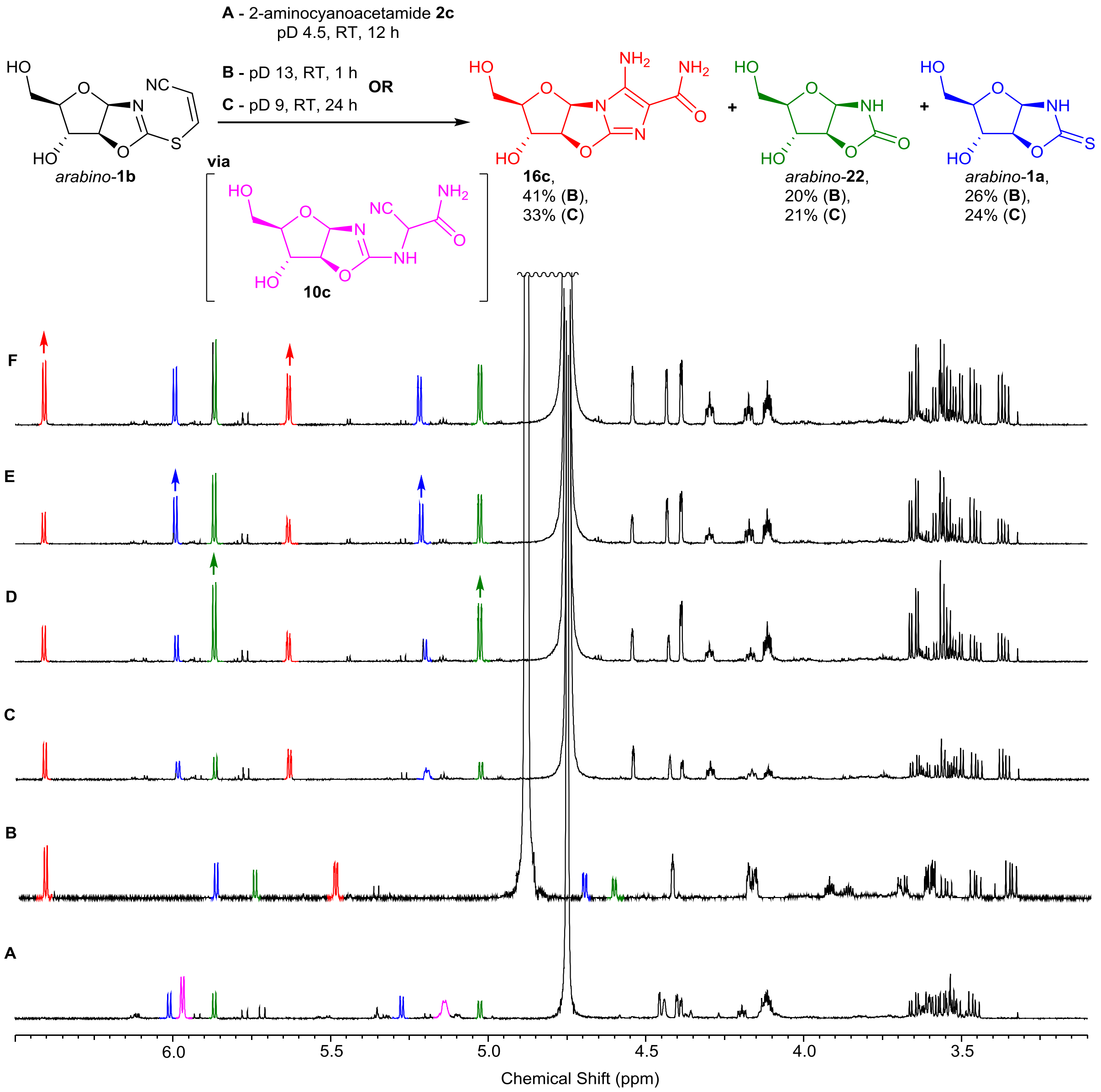

Supplementary Fig. 23. *- ^{1}H NMR spectra (600 MHz, D_2O, 3.1 – 7.0 ppm) showing reaction of (S-Z-cyanovinyl)-arabinofuranosyl oxazolidinone thione (arabino-**1b**) with 2-aminocyanoacetamide (**2c**). A - arabino-**1b** (250mM) and **2c** (500mM) in D_2O (500 µL) at pD 4.5 incubated at RT for 12 h. Calibration to an internal standard gave a yield of 50% (aminooxazoline **10c**, 5.96 ppm). B – aliquot of A incubated at pD 13 at RT for 1 h giving a yield of 41%* 2,2'-anhydro-5-aminoimidazole-4-carboxamide-β-furanosylarabinoside *(**16c**). C - an aliquot (50 µL) of A was added to ammonium hydroxide in D_2O (450 µL, 100mM), the solution was adjusted to pD 9 with NaOD (4M) and incubated for 24 h at RT. Calibration to an internal standard gave yields of 33% **16c**, 24% arabinofuranosyl oxazolidinone thione (arabino-**1a**), 21% arabinofuranosyl oxazolidinone (arabino-**22**). D – spiked with arabino-**22.** E – spiked with arabino-**1a**. F – spiked with **16c**.*

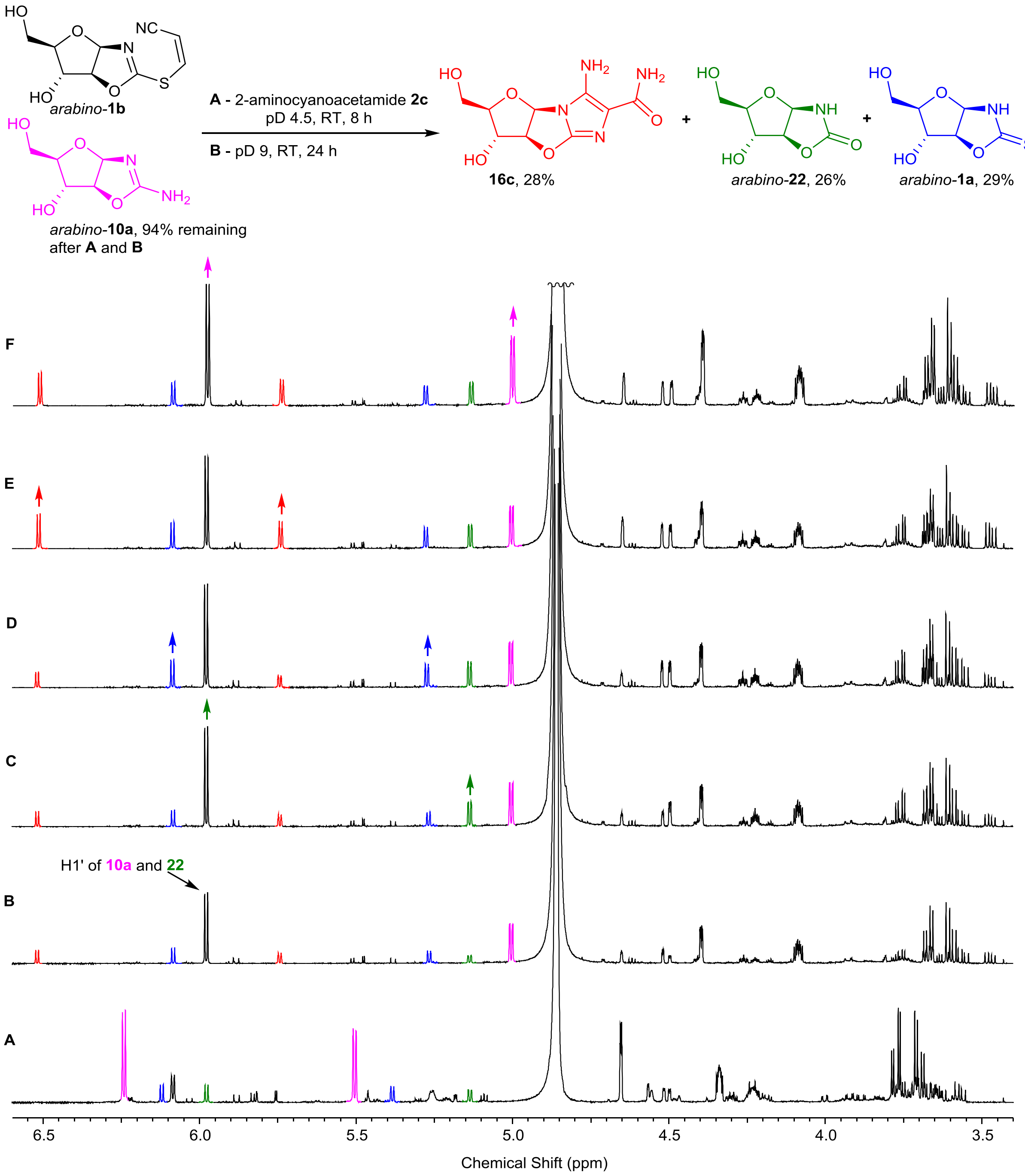

Supplementary Fig. 24. *- ^{1}H NMR spectra (600 MHz, D_2O, 3.4 – 6.6 ppm) showing reaction of (S-Z-cyanovinyl)-arabinofuranosyl oxazolidinone thione (arabino-**1b**) with 2-aminocyanoacetamide (**2c**) in the presence of arabinofuranosyl aminooxazoline (arabino-**10a**). A - arabino-**1b** (250mM), arabino-**10a** (250mM) and **2c** (500mM) in D_2O (500 µL) at pD 4.5 incubated at RT for 8 h. B - an aliquot (50 µL) was added to ammonium hydroxide in D_2O (450 µL, 100mM), the solution was adjusted to pD 9 with NaOD (4M) and incubated for 24 h at RT. Calibration to an internal standard gave yields of 28%* 2,2'-anhydro-5-aminoimidazole-4-carboxamide-β-furanosylarabinoside *(**16c**), 29% arabinofuranosyl oxazolidinone thione (arabino-**1a**), 26% arabinofuranosyl oxazolidinone (arabino-**22**), additionally 94% (arabino-**10a**) remained. C – spiked with arabino-**22.** D – spiked with arabino-**1a**. E – spiked with **16c**. F – spiked with arabino-**10a.***

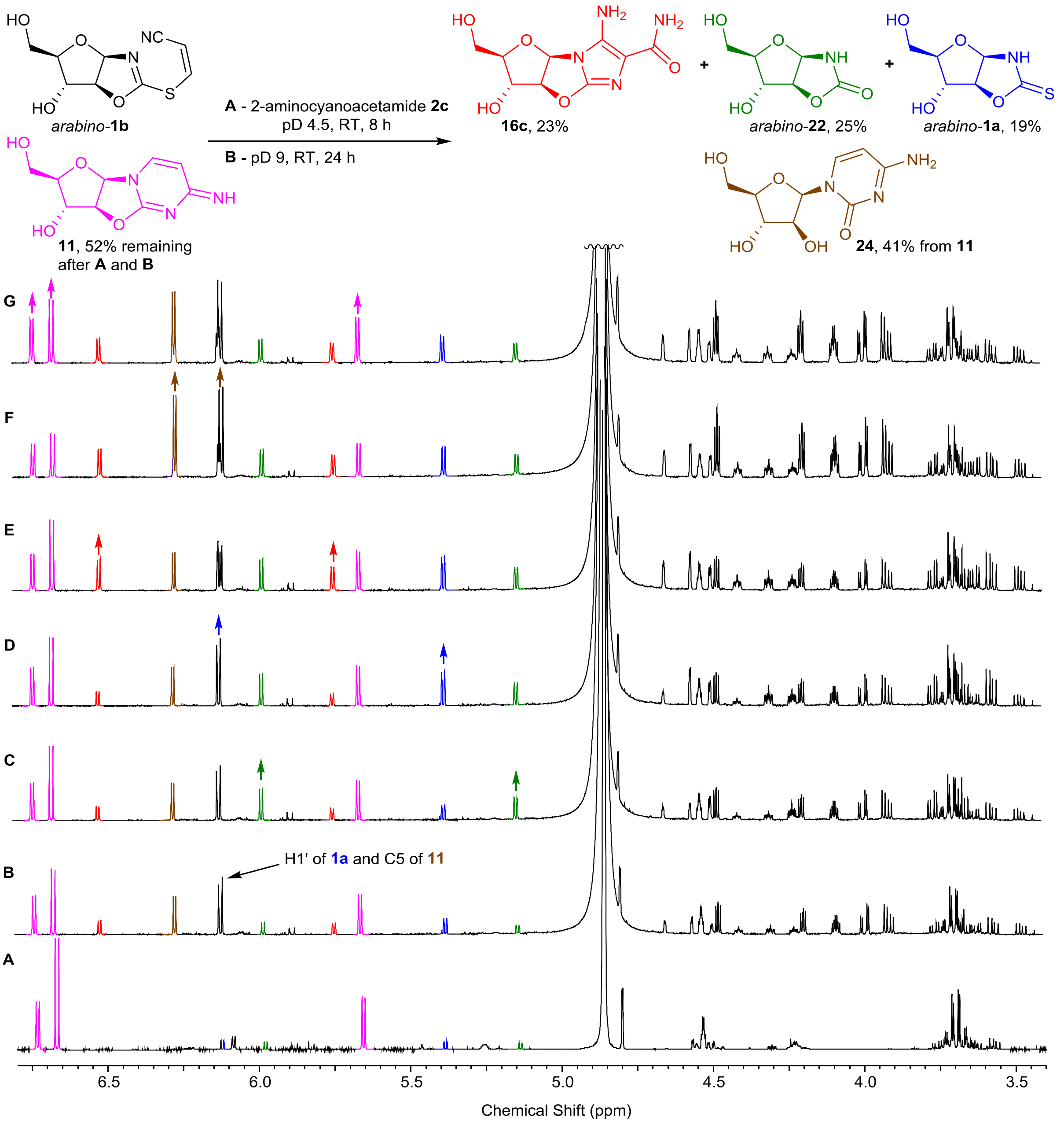

Supplementary Fig. 25. *- ^{1}H NMR spectra (600 MHz, D_2O, 3.4 – 6.8 ppm) showing reaction of (S-Z-cyanovinyl)-arabinofuranosyl oxazolidinone thione (arabino-**1b**) with 2-aminocyanoacetamide **2c** in the presence of ancitabine (**11**). A - arabino-**1b** (250mM), **11** (250mM) and **2c** (500mM) in D_2O (500 µL) at pD 4.5 incubated at RT for 8 h. B - an aliquot (50 µL) was added to ammonium hydroxide in D_2O (450 µL, 100mM), the solution was adjusted to pD 9 with NaOD (4M) and incubated for 24 h at RT. Calibration to an internal standard gave yields of 23%* 2,2'-anhydro-5-aminoimidazole-4-carboxamide-β-furanosylarabinoside *(**16c**), 19% arabinofuranosyl oxazolidinone thione (arabino-**1a**), 25% arabinofuranosyl oxazolidinone (arabino-**22**), additionally hydrolysis of **11** gave 41% yield of cytarabine (**24**) with 52% **11** remaining. C – spiked with arabino-**22**. D – spiked with arabino-**1a**. E – spiked with **16c**. F – spiked with **24**. G – spiked with **11**.*

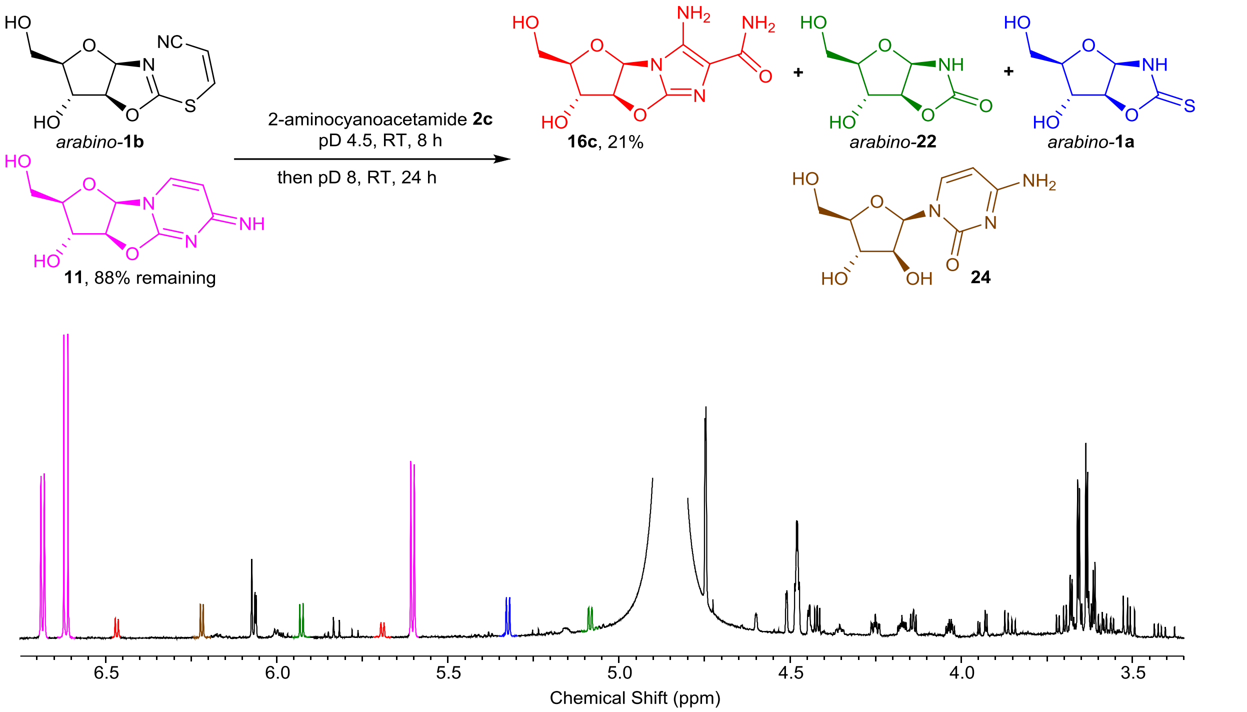

Supplementary Fig. 26. *- ^{1}H NMR spectrum (600 MHz, D_2O, 3.35 – 6.75 ppm) showing reaction of (S-Z-cyanovinyl)-arabinofuranosyl oxazolidinone thione (arabino-**1b**) with 2-aminocyanoacetamide (**2c**) in the presence of ancitabine (**11**). When aliquot from A (Supplementary Fig. 25) is incubated at pD 8 for 24 h a yield of 21%* 2,2'-anhydro-5-aminoimidazole-4-carboxamide-β-furanosylarabinoside *(**16c**) is achieved with 88% **11** remaining.*

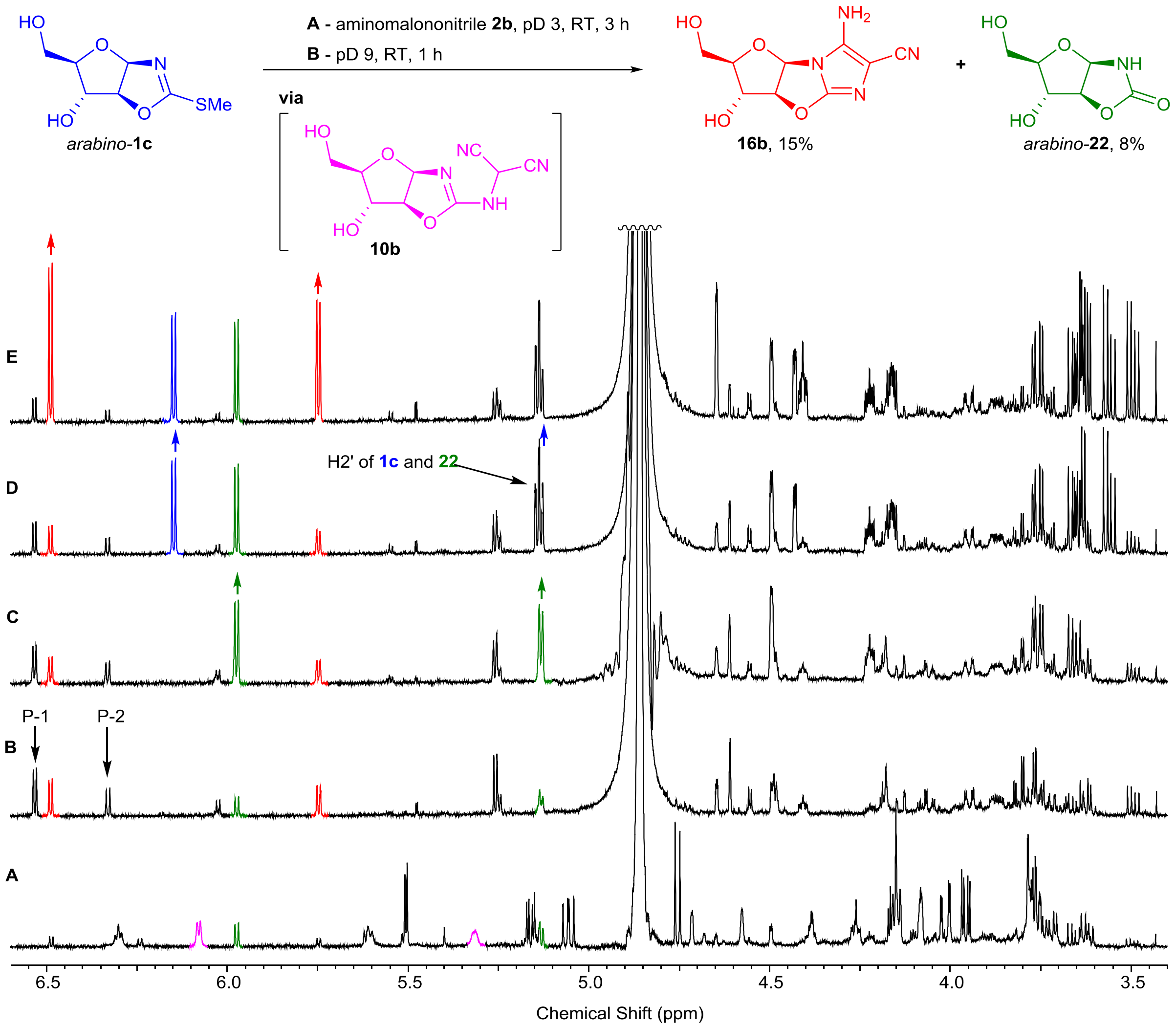

Supplementary Fig. 27. *– ^{1}H NMR spectra (600 MHz, D_2O, 3.4 – 6.6 ppm) showing reaction of arabinofuranosyl-(2-thiomethyl)-oxazolidinone (arabino-**1c**) with aminomalononitrile (**2b**). A – arabino-**1c** (250mM) and aminomalononitrile p-toluenesulfonate (1M) at pD 3 and RT incubated for 3 h. Aminooxazoline **10b** was produced in a 15% yield. B – aliquot (50 µL) from A was added to ammonium hydroxide in D_2O (450 µL, 100mM), the solution was adjusted to pD 9 with NaOD (4M) and incubated for 1 h at RT. By-products P-1 and P-2 are derived from arabino-**1c** and observed upon incubation of arabino-**1c** alone under comparable conditions. P-1 and P-2 are suspected to be the products of (intermolecular) S-N methyl migration. Calibration to an internal standard gave yields of 15% 2,2'-anhydro-5-aminoimidazole-4-carbonitrile-β-furanosylarabinoside (**16b**) and 8% arabinofuranosyl oxazolidinone (arabino-**22**). C – spiked with arabino-**22**. D - spiked with arabino-**1c**. E – spiked with **16b**.*

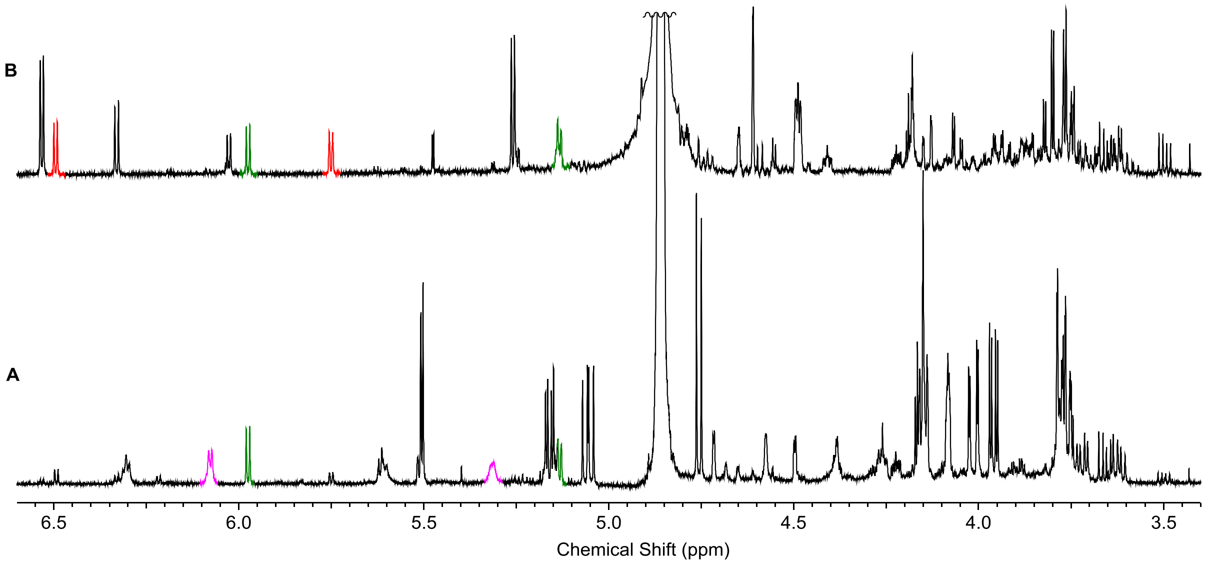

Supplementary Fig. 28. *– ^{1}H NMR spectra (600 MHz, D_2O, 3.4 – 6.6 ppm) showing reaction of arabinofuranosyl-(2-thiomethyl)-oxazolidinone (arabino-**1c**) with aminomalononitrile (**2b**). A – arabino-**1c** (250mM) and aminomalononitrile p-toluenesulfonate (500mM) at pD 3 and RT incubated for 3 h. B – aliquot (50 µL) from A was added to ammonium hydroxide in D_2O (450 µL, 100mM), the solution was adjusted to pD 9 with NaOD (4M) and incubated for 1 h at RT. Calibration to an internal standard gave yields of 10%* 2,2'-anhydro-5-aminoimidazole-4-carbonitrile-β-furanosylarabinoside *(**16b**) and 10% arabinofuranosyl oxazolidinone (arabino-**22**). See supplementary Fig. 27. for reaction scheme.*

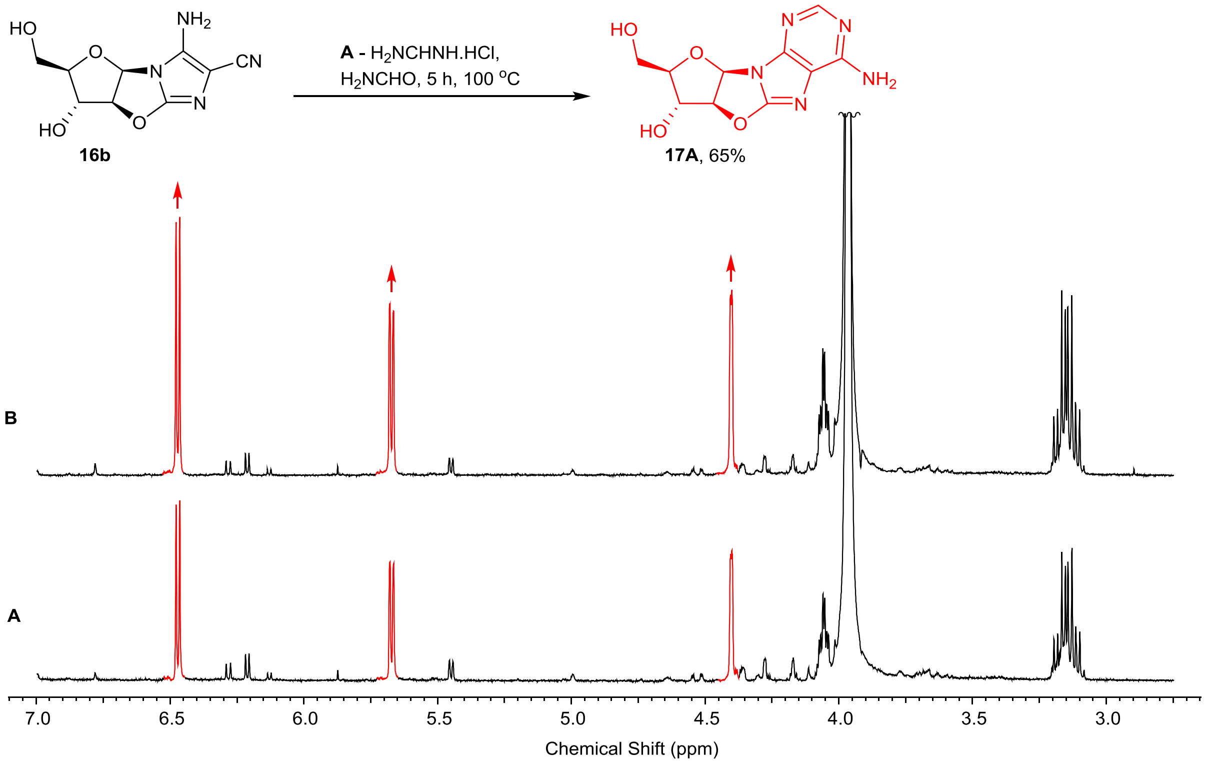

Supplementary Fig. 29. *- ¹H NMR spectra (600 MHz, D6-DMSO, 2.65 – 7.1 ppm) to show the reaction of 2,2'-anhydro-5-aminoimidazole-4-carbonitrile-β-furanosylarabinoside (**16b**) and formamidine. A – **16b** (40 mM) and formamidine.HCl (400mM) in formamide after 5 h at 100 °C. Calibration to an internal standard gave a yield of 65% 8,2'-O-anhydro-9-β-arabinofuranosyl-cycloadenosine (**17A**). B – spiked with **17A**.*

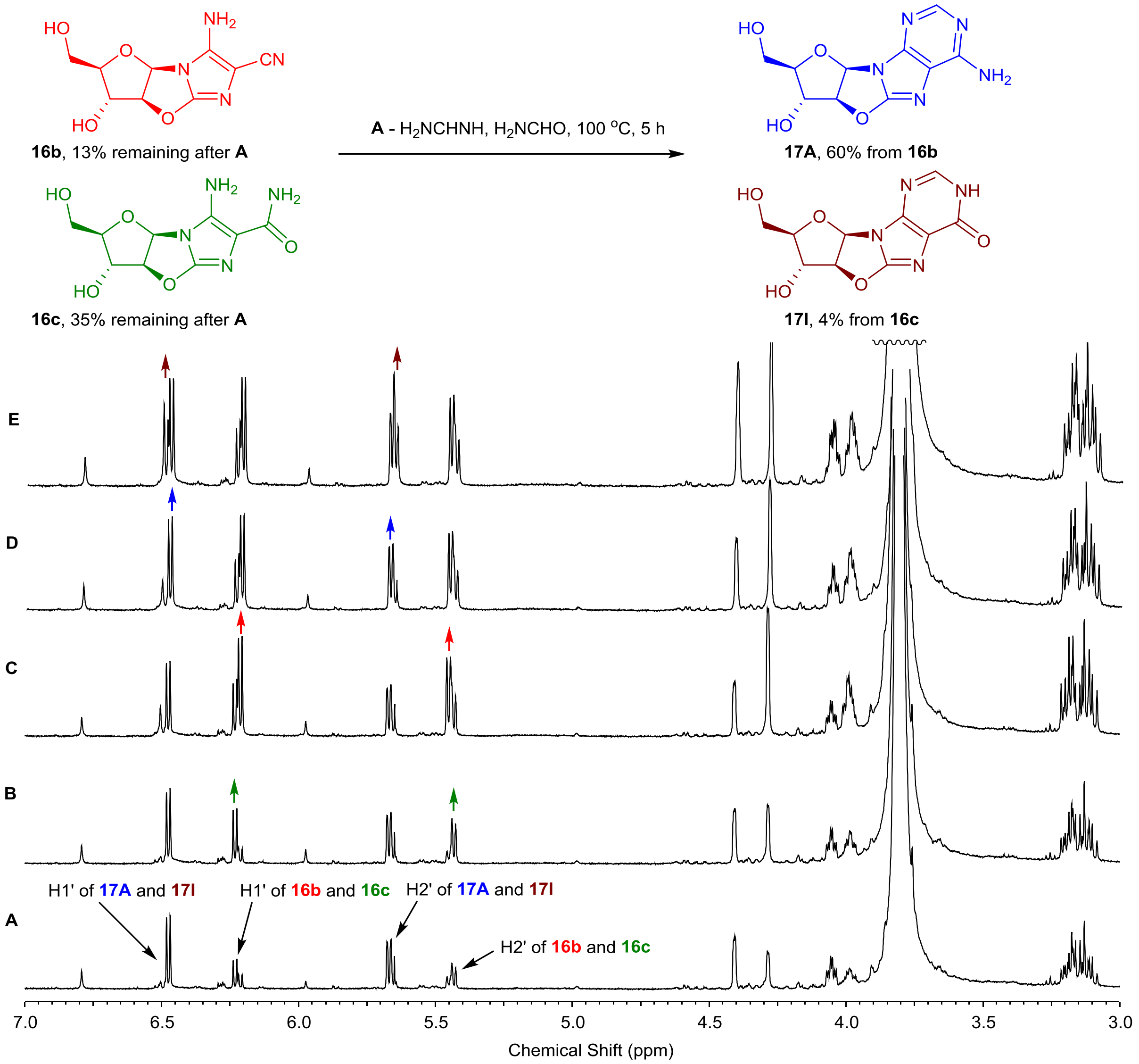

Supplementary Fig. 30. *- ^{1}H NMR spectra (600 MHz, D_6-DMSO, 3.0 – 7.0 ppm) to show the reaction of 2,2'-anhydro-5-aminoimidazole-4-carbonitrile-β-furanosylarabinoside (**16b**) 2,2'-anhydro-5-aminoimidazole-4-carboxamide-β-furanosylarabinoside (**16c**) with formamidine. A – **16b** (40 mM), **16c** (40 mM) and formamidine.HCl (400 mM) in formamide after 5 hr at 100 °C. Calibration to an internal standard gave yields of 60% 8,2'-O-anhydro-9-β-arabinofuranosyl-cycloadenosine (**17A**) and 4% 8,2'-O-anhydro-9-β-arabinofuranosyl-cycloinosine (**17I**) with 13% **16b** and 35% **16c** remaining. B – spiked with **16c**. C – spiked with **16b**. D – spiked with **17A**. E – spiked with **17I**.*

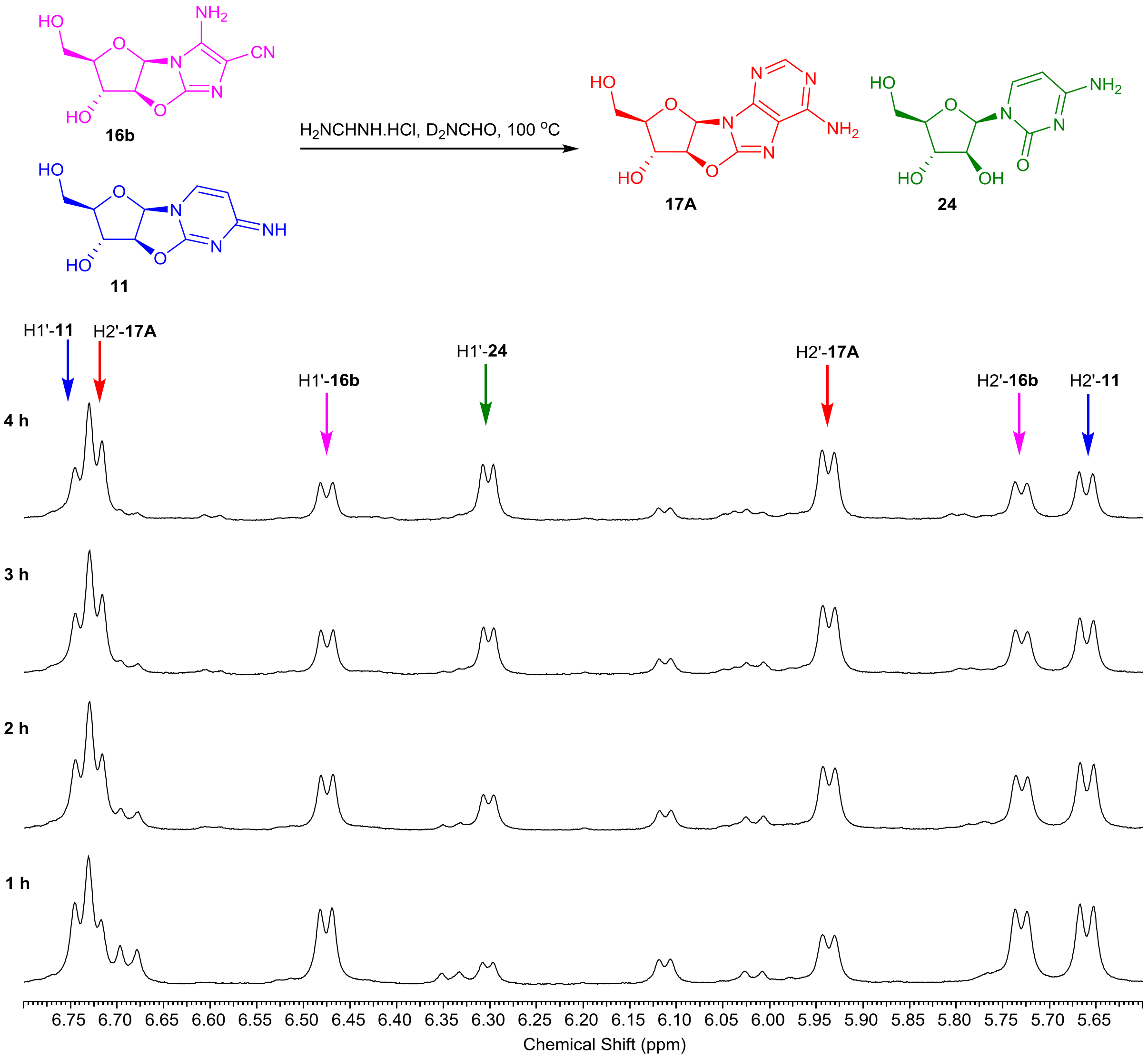

Supplementary Fig. 31. *- ^{1}H NMR spectra (400 MHz, D_2-formamide, 5.5 – 6.8 ppm) to show the reaction of 2,2'-anhydro-5-aminoimidazole-4-carbonitrile-β-furanosylarabinoside (**16b**) and ancitabine (**11**) with formamidine. **16b** (40 mM), **11** (40 mM) and formamidine.HCl (400 mM) were heated in formamide at 100 °C and the mixture was analysed at 1 h intervals. After 3 h, calibration to an internal standard (DSS) gave yields of 53% 8,2'-O-anhydro-9-β-arabinofuranosyl-cycloadenosine (**17A**) and 38% cytarabine (**24**) with 45% **11** and 33% **16b** remaining. After 4 h the reaction was concentrated to dryness and dissolved in DMSO for analysis and spiking – see Supplementary Fig. 32.*

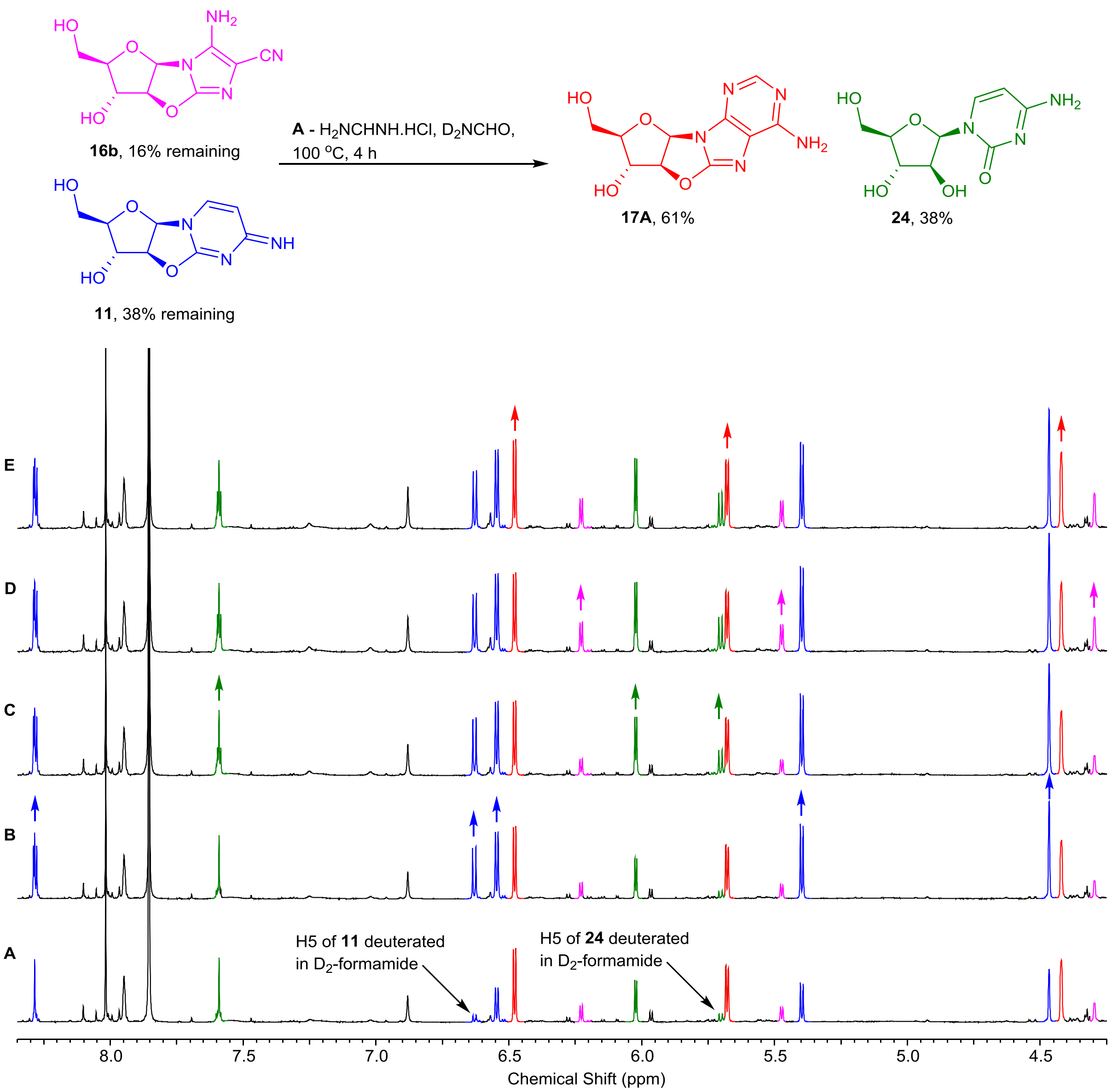

Supplementary Fig. 32. *- ^{1}H NMR spectra (600 MHz, D_6-DMSO, 4.25 – 8.35 ppm) to show the reaction of 2,2'-anhydro-5-aminoimidazole-4-carbonitrile-β-furanosylarabinoside (**16b**) and ancitabine (**11**) with formamidine. A - **16b** (40 mM), **11** (40 mM) and formamidine.HCl (400 mM) in formamide at 100 °C after 4 h. Calibration to an internal standard (DSS) gave yields of 61% 8,2'-O-anhydro-9-β-arabinofuranosyl-cycloadenosine (**17A**) and 38% cytarabine (**24**) with 38% **11** and 16% **16b** remaining. B – spiked with **11**. C – spiked with **24**. D – spiked with **16b**. E – spiked with **17A**.*

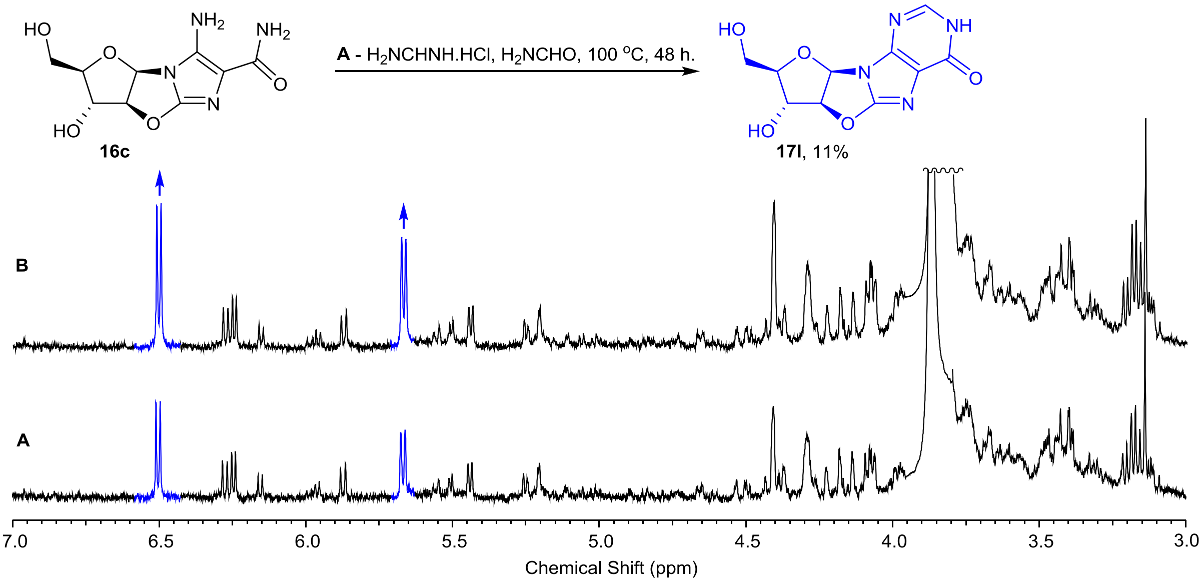

Supplementary Fig. 33. *- ^{1}H NMR spectra (600 MHz, D_6-DMSO, 3.0 – 7.0 ppm) to show the reaction of 2,2'-anhydro-5-aminoimidazole-4-carboxamide-β-furanosylarabinoside (**16c**) with formamidine. A – **16c** (40 mM) and formamidine.HCl (400 mM) in formamide after 48 h at 100 °C. B – spiked with 8,2'-O-anhydro-9-β-arabinofuranosyl-cycloinosine (**17l**).*

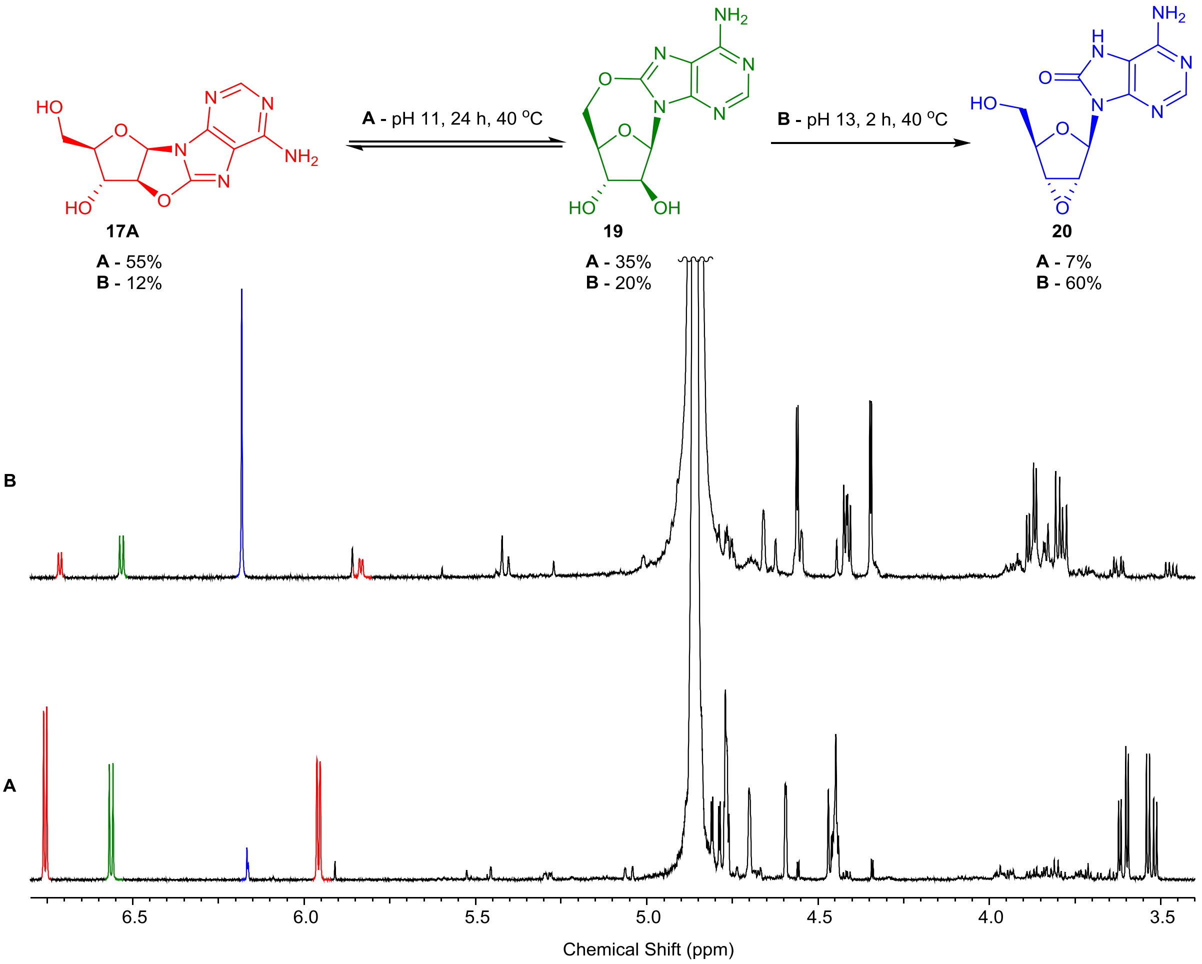

Supplementary Fig. 34. *– ^{1}H NMR (600, MHz, 3.4 – 6.8 ppm) showing isomerisation of anhydropurines. 8,2'-O-anhydro-9-β-arabinofuranosyl-cycloadenosine (**17A**; 20mM) was incubated at pH 11 at 40 °C. A – reaction after 24 h showing a mixture of **17A** (55%), 8,5'-O-anhydro-9-β-arabinofuranosyl-cycloadenosine (**19**; 35%) and 2',3'-epoxy-9-β-ribofuranoside-8-oxoadenosine (**20**; 7%). The pH was increased to 13 and the reaction was again incubated at 40 °C. B – reaction after 2 h showing a mixture of **17A** (12%), **19** (20%) and **20** (60%).*

*These isomerisations have previously been reported by Reese[11] and Ikehara.[12] Furthermore, Reese also reported that epoxide formation is reversible, and **20** converts to **17A** at pH 11 at room temperature over 48 h.*

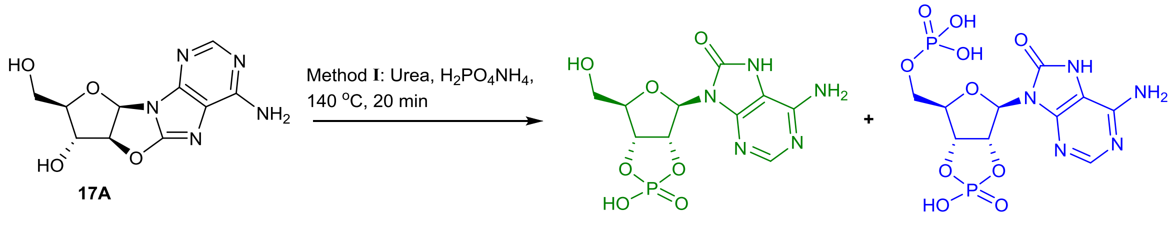

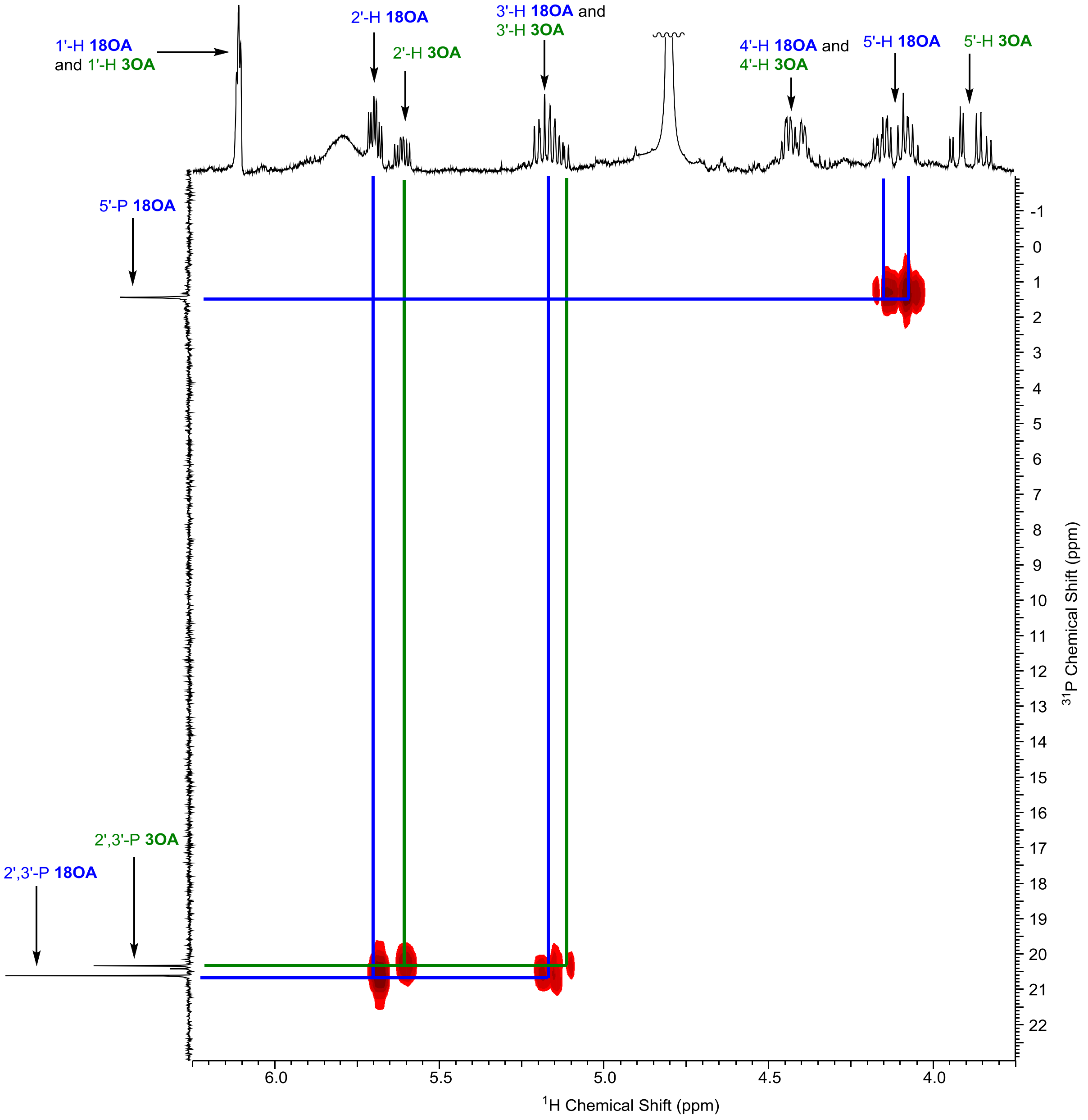

Supplementary Fig. 35. *- ^{1}H - ^{31}P HMBC NMR spectrum (400/162 MHz, D_2O, 3.75 – 6.25; -2.0 – 23.0 ppm) to show phosphorylation of 8,2'-O-anhydro-9-β-arabinofuranosyl-cycloadenosine (**17A**) by method **I**.*

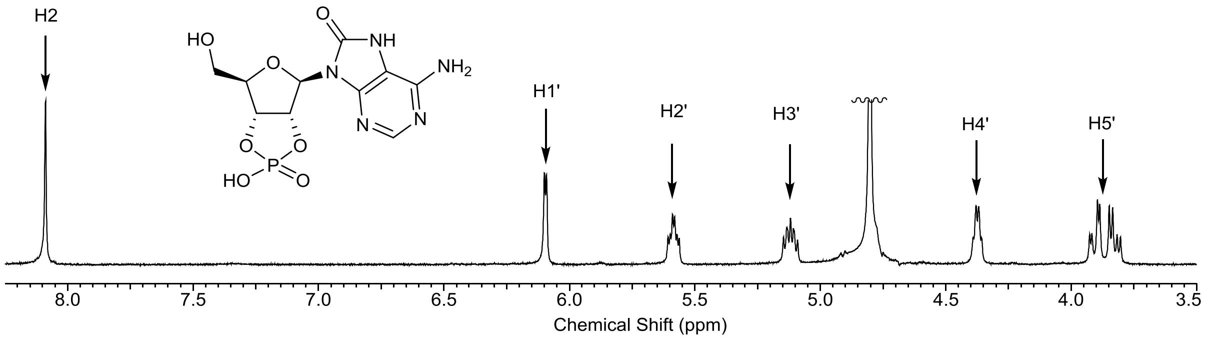

Supplementary Fig. 36. *– ^{1}H NMR spectrum (400 MHz, D_2O, 3.5 – 8.25 ppm) of HPLC purified (8.39 min) 8-oxo-adenosine-2',3'-cyclic phosphate (**3OA**).*

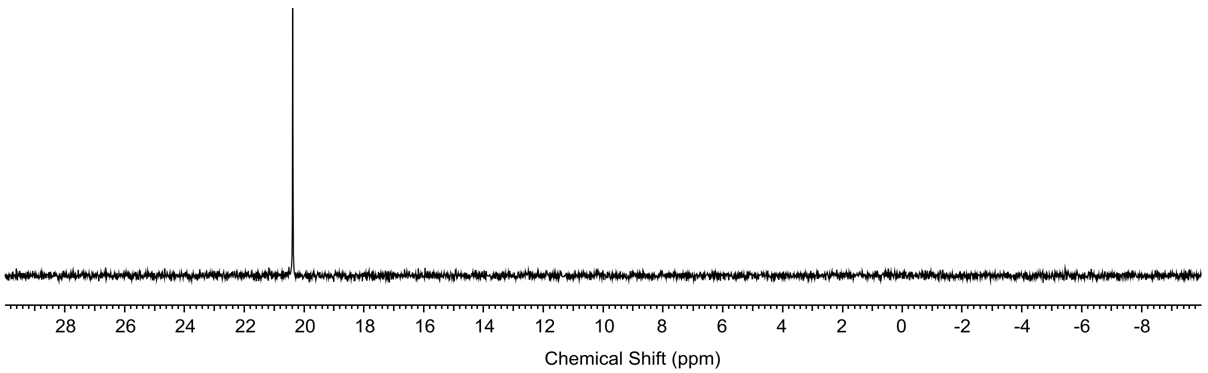

Supplementary Fig. 37. *– ^{31}P NMR spectrum (162 MHz, D_2O, -10 – 30 ppm) of HPLC purified (8.39 min) 8-oxo-adenosine-2',3'-cyclic phosphate (**3OA**).*

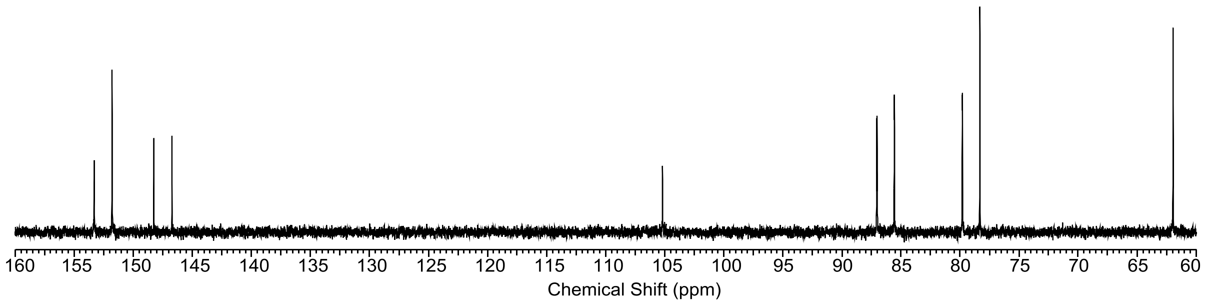

Supplementary Fig. 38. *– ^{13}C NMR spectrum (151 MHz, D_2O, 60 – 160 ppm) of HPLC purified (8.39 min) 8-oxo-adenosine-2',3'-cyclic phosphate (**3OA**).*

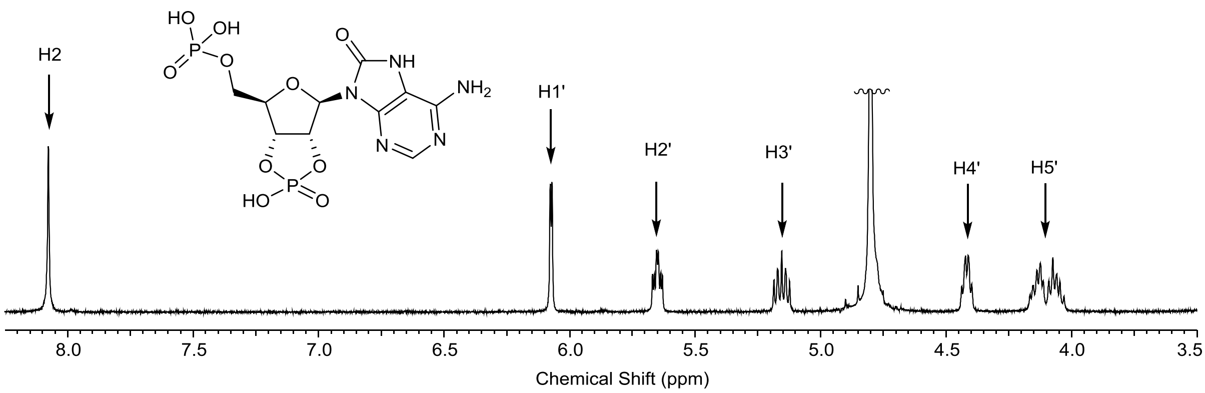

Supplementary Fig. 39. *– ^{1}H NMR spectrum (400 MHz, D_2O, 3.5 – 8.25 ppm) of HPLC purified (2.44 min) 8-oxo-adenosine-2',3'-cyclic-5' bisphosphate (**18OA**).*

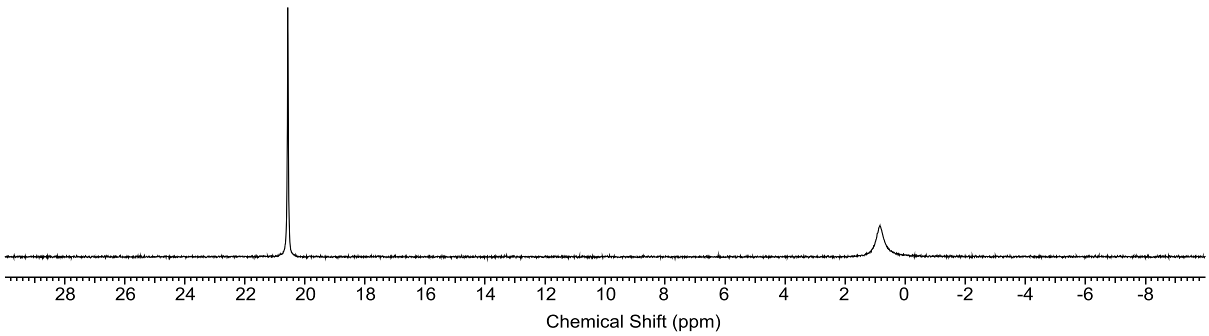

Supplementary Fig. 40. *– ^{31}P NMR spectrum (162 MHz, D_2O, -10 – 30 ppm) of HPLC purified (2.44 min) 8-oxo-adenosine-2',3'-cyclic-5' bisphosphate (**18OA**).*

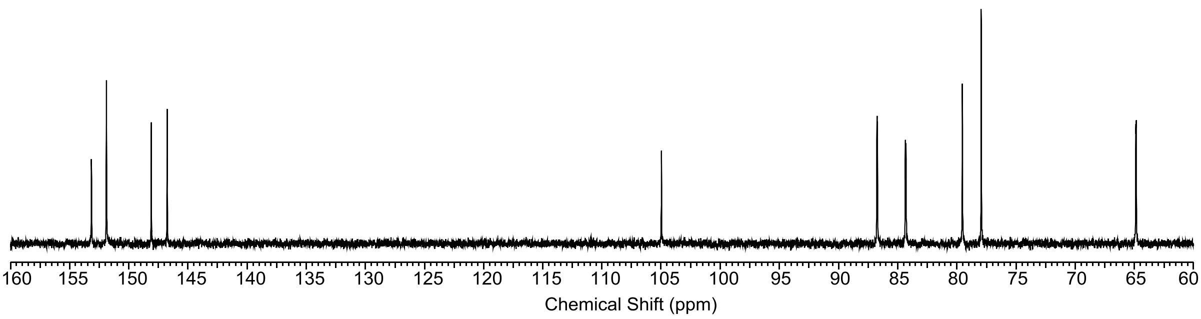

Supplementary Fig. 41. *– ^{13}C NMR spectrum (151 MHz, D_2O, 60 –160 ppm) of HPLC purified (2.44 min) 8-oxo-adenosine-2',3'-cyclic-5' bisphosphate (**18OA**).*

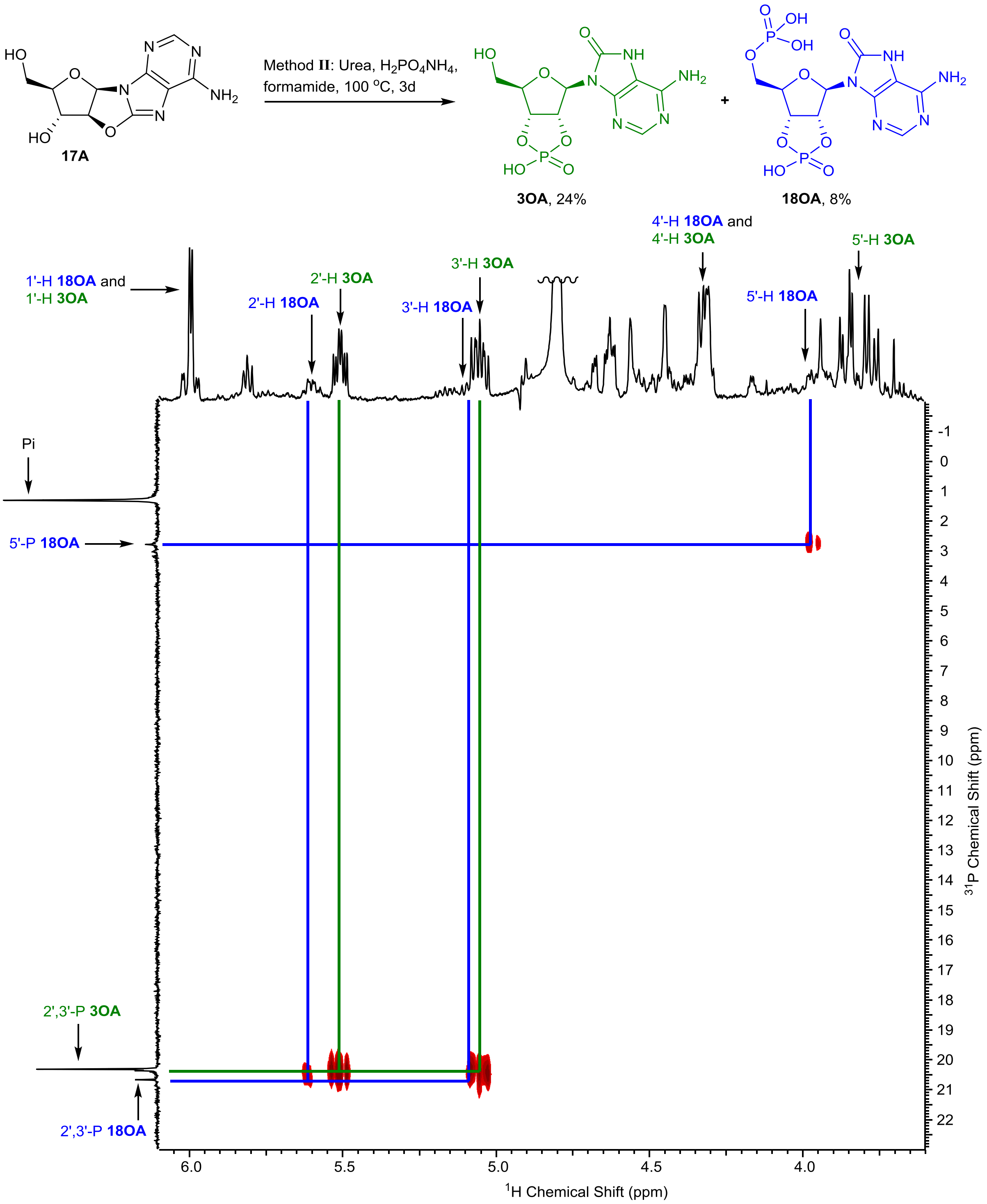

Supplementary Fig. 42. *- ^{1}H - ^{31}P HMBC NMR spectrum (400/162 MHz, D_2O, 3.6 – 6.1; -2.0 – 23.0 ppm) to show phosphorylation of 8,2'-O-anhydro-9-β-arabinofuranosyl-cycloadenosine (**17A**) by method **II**.*

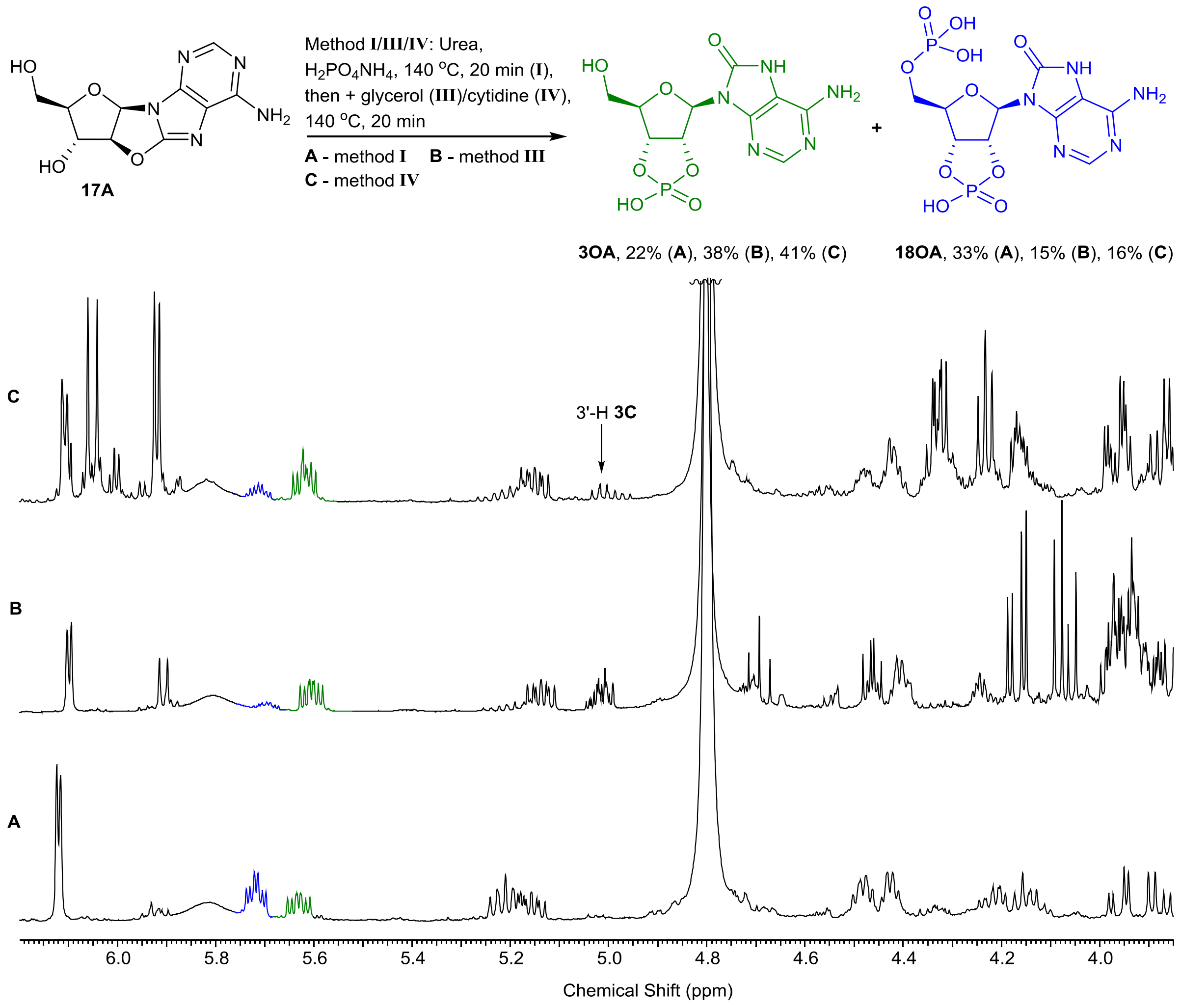

Supplementary Fig. 43. *– ^{1}H NMR spectrum (400 MHz, D_2O, 3.85 – 6.2 ppm) showing phosphorylation of 8,2'-O-anhydro-9-β-arabinofuranosyl-cycloadenosine (**17A**).* ***A*** *– phosphorylation of **17A** by method **I** (Supplementary Fig 35.).* ***B*** *– phosphorylation of **17A** by method **III**.* ***C*** *– phosphorylation of **17A** by method **IV**.*

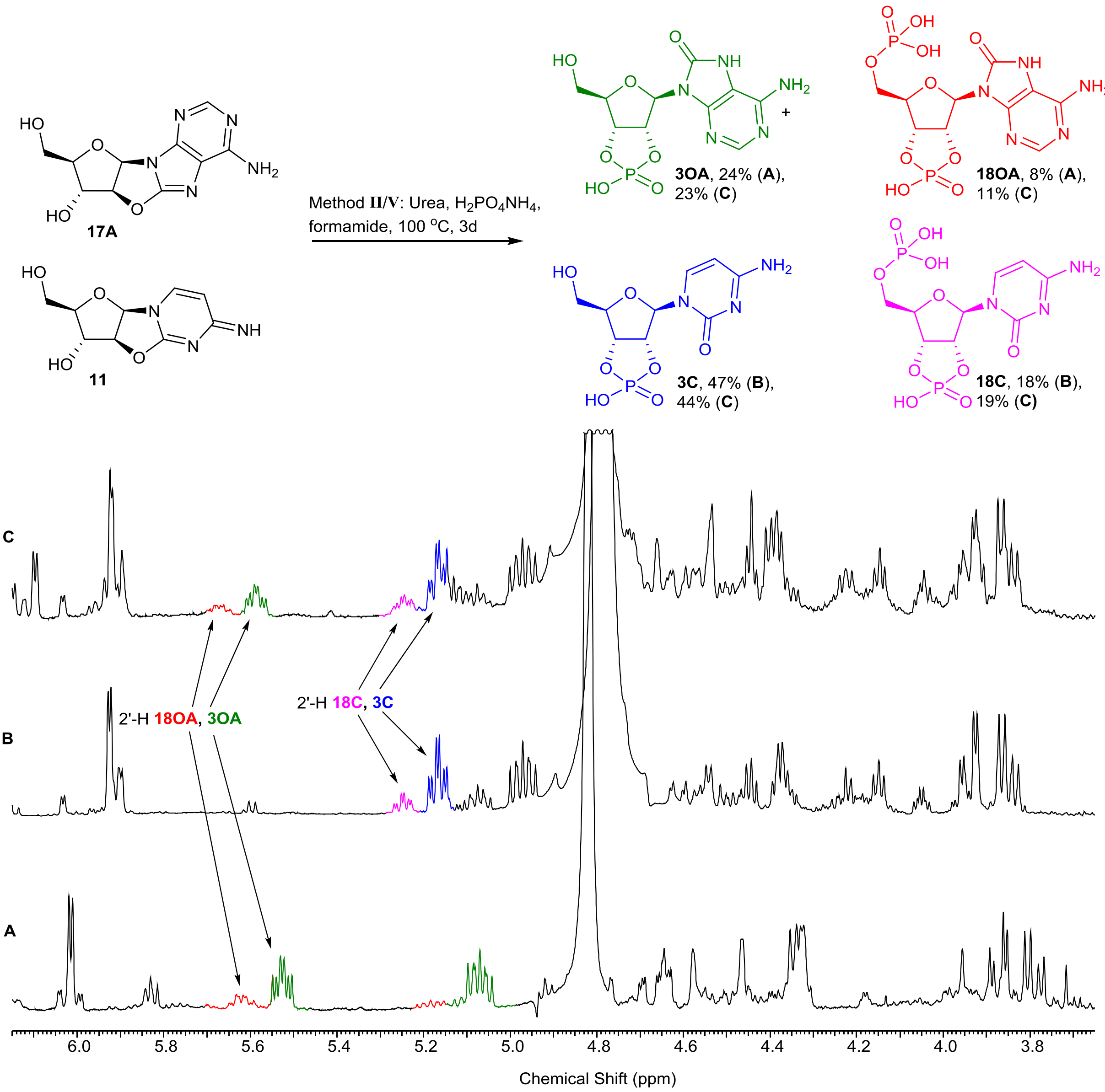

Supplementary Fig. 44. *– ^{1}H NMR spectrum (400 MHz, D_2O, 3.65 – 6.15 ppm) showing co-phosphorylation of ancitabine **11** and 8,2'-O-anhydro-9-β-arabinofuranosyl-cycloadenosine (**17A**). **A** – phosphorylation of **17A** by method **II** (Supplementary Fig 42.). **B** – phosphorylation of **11** by method **II**. **C** – co-phosphorylation of **17A** and **11** (1:1) by method **V**.*

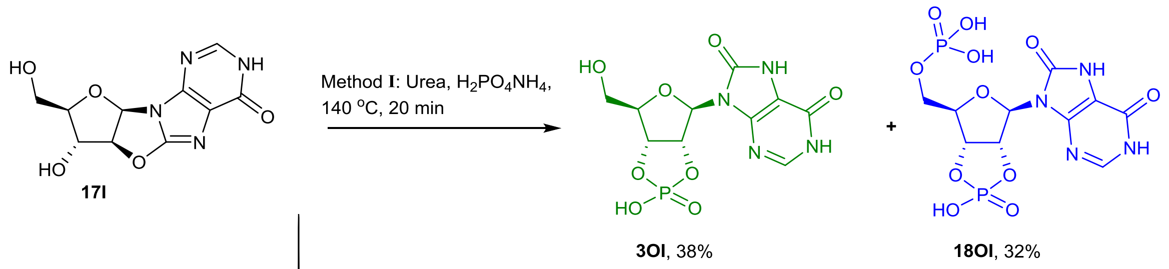

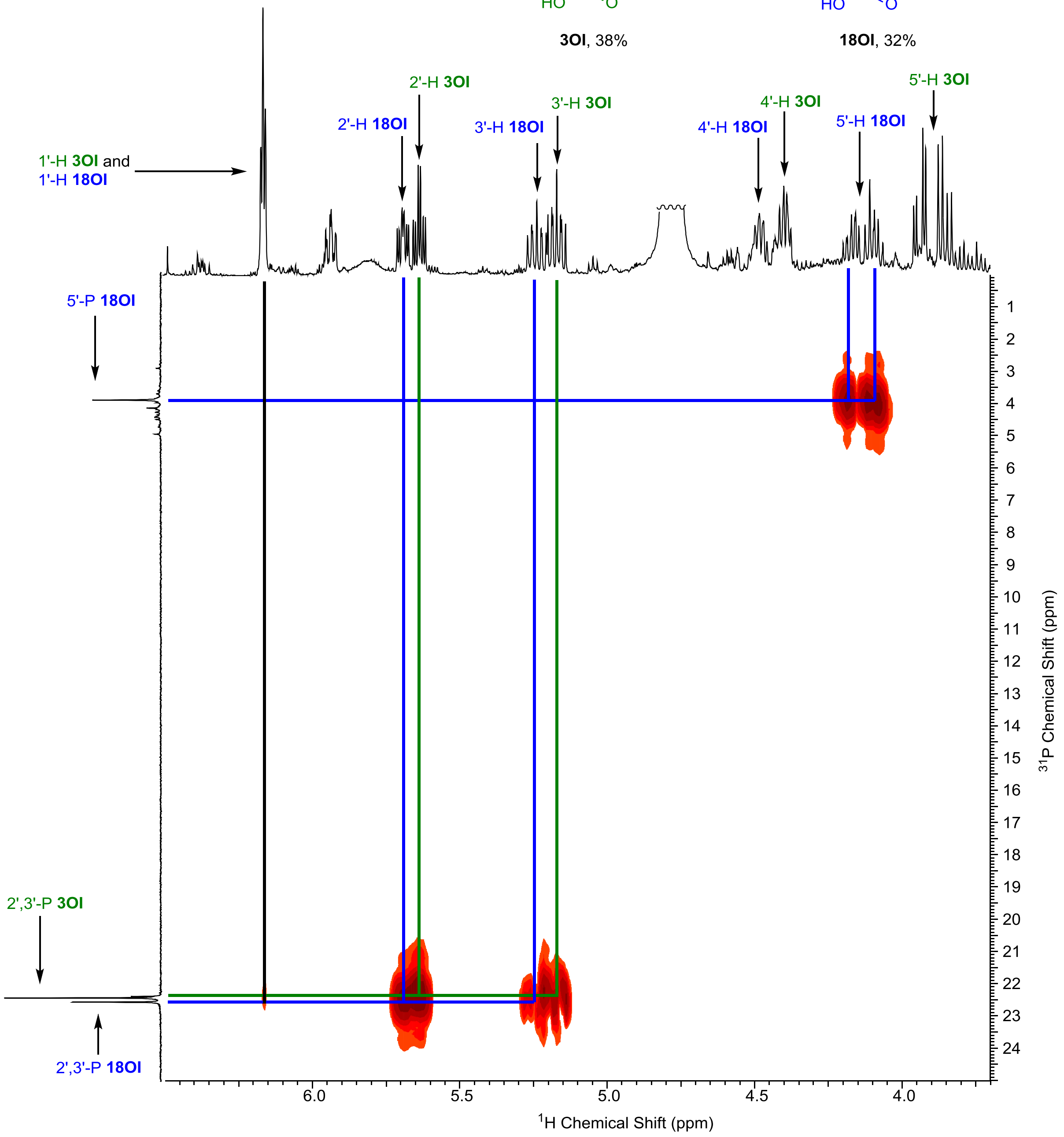

Supplementary Fig. 45. *- ^{1}H - ^{31}P HMBC NMR spectrum (400/162 MHz, D_2O, 3.7 – 6.5; 0.0 – 25.0 ppm) to show phosphorylation of 8,2'-O-anhydro-9-β-arabinofuranosyl-cycloinosine (**17l**) by method **I**.*

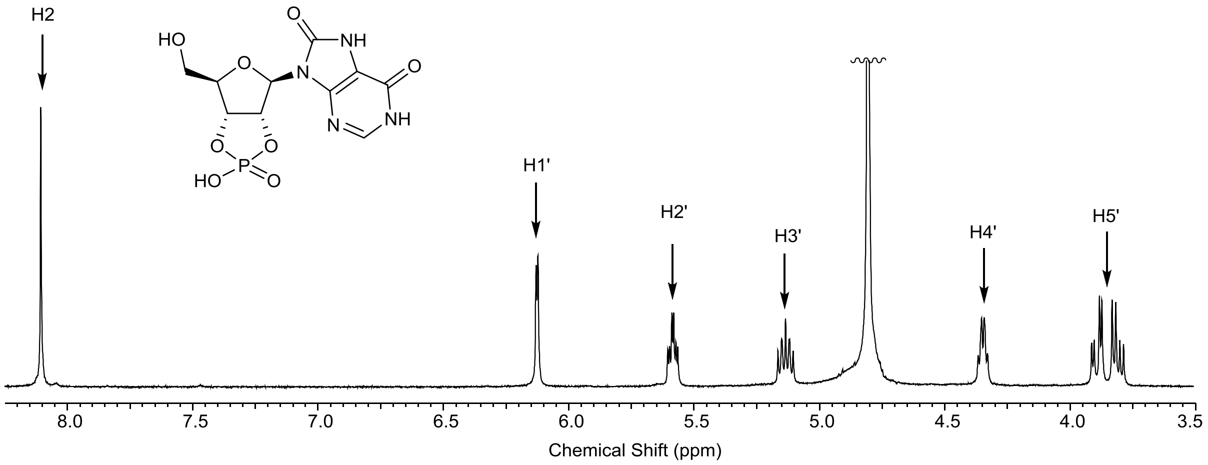

Supplementary Fig. 46. *– ^{1}H NMR spectrum (400 MHz, D_2O, 3.5 – 8.25 ppm) of HPLC purified (7.61 min) 8-oxo-inosine-2',3'-cyclic phosphate (**3OI**).*

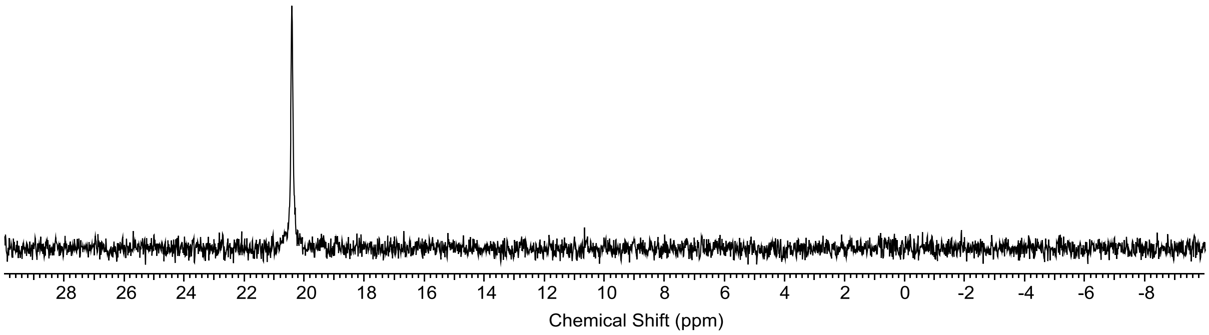

Supplementary Fig. 47. *– ^{31}P NMR spectrum (162 MHz, D_2O, -10 – 30 ppm) of HPLC purified (7.61 min) 8-oxo-inosine-2',3'-cyclic phosphate (**3OI**).*

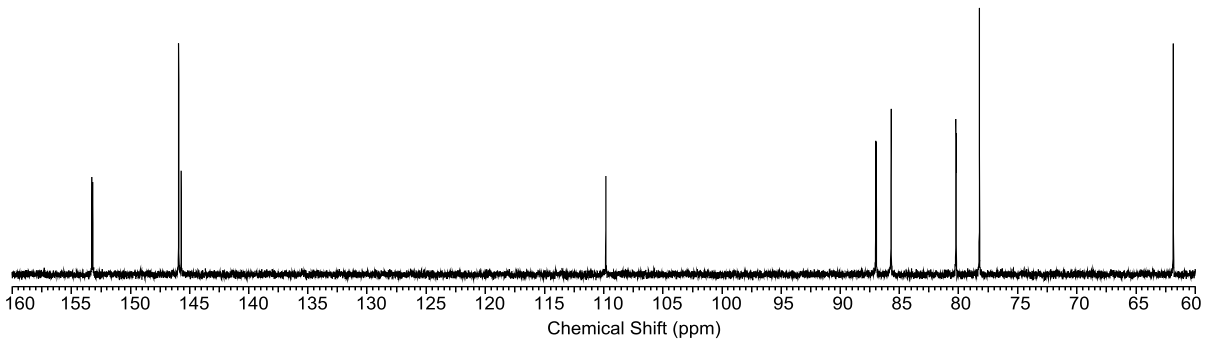

Supplementary Fig. 48. *– ^{13}C NMR spectrum (151 MHz, D_2O, 60 – 160 ppm) of HPLC purified (7.61 min) 8-oxo-inosine-2',3'-cyclic phosphate (**3OI**).*

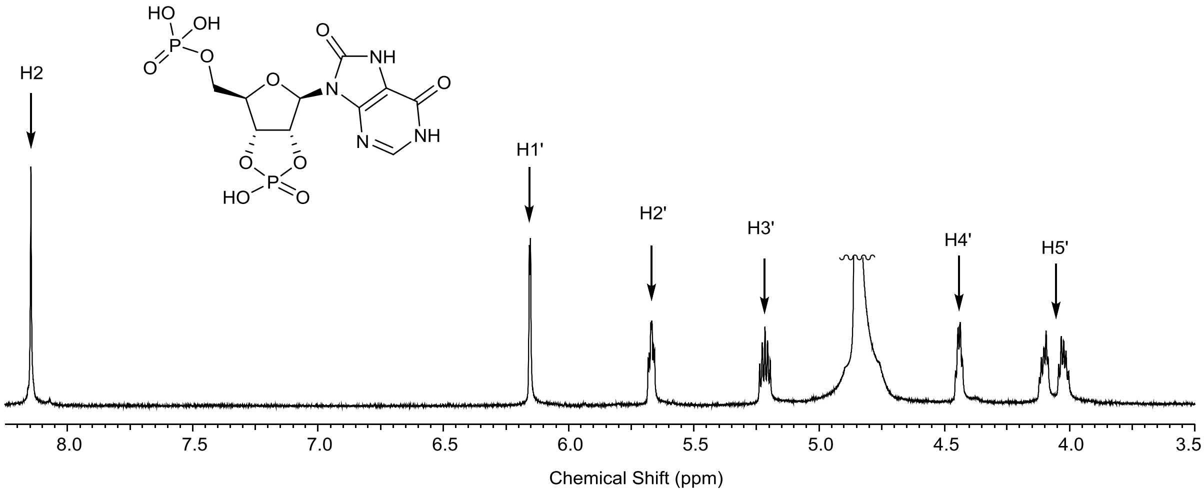

Supplementary Fig. 49. *– ^{1}H NMR spectrum (600 MHz, D_2O, 3.5 – 8.25 ppm) of HPLC purified (2.16 min) 8-oxo-inosine-2',3'-cyclic-5' bisphosphate (**18OI**).*

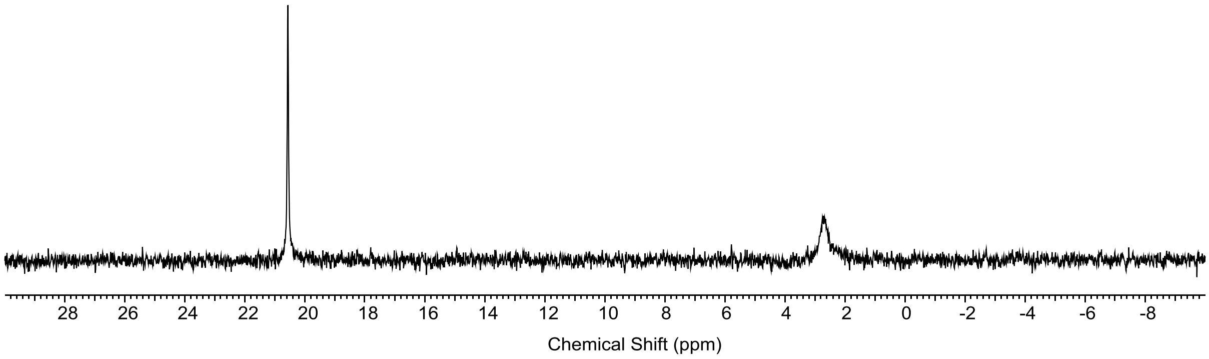

Supplementary Fig. 50. *– ^{31}P NMR spectrum (162 MHz, D_2O, -10 – 30 ppm) of HPLC purified (2.16 min) 8-oxo-inosine-2',3'-cyclic-5' bisphosphate **18OI**.*

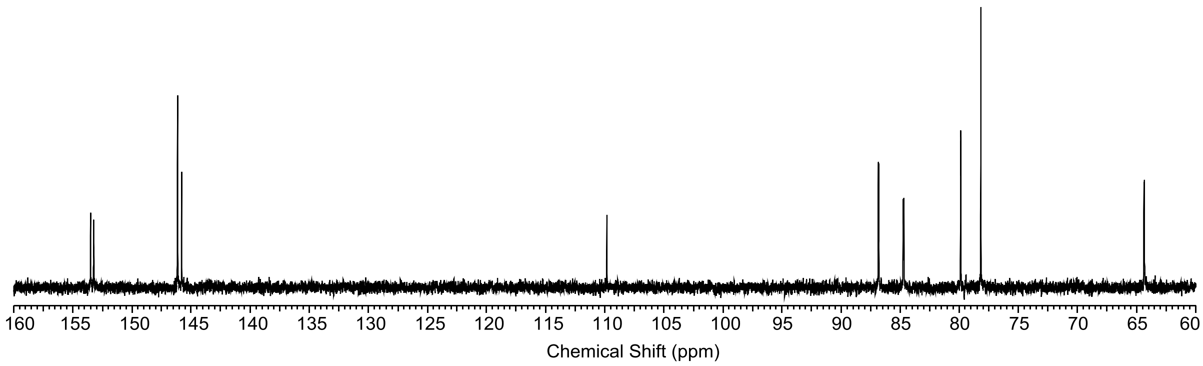

Supplementary Fig. 51. *– ^{13}C NMR spectrum (151 MHz, D_2O, 60 – 160 ppm) of HPLC purified (2.16 min) 8-oxo-inosine-2',3'-cyclic-5' bisphosphate (**18OI**).*

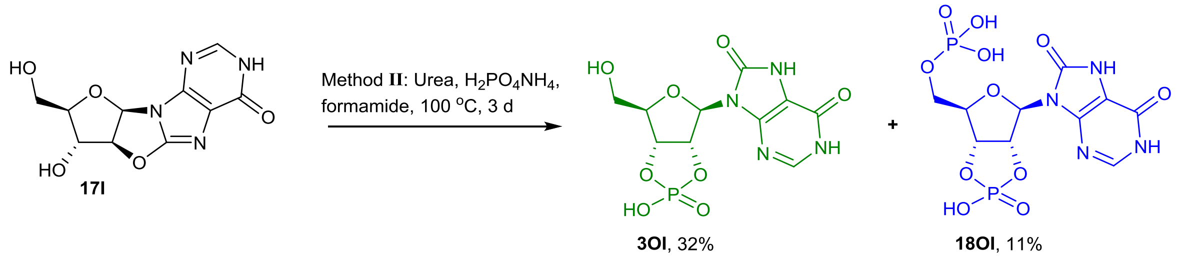

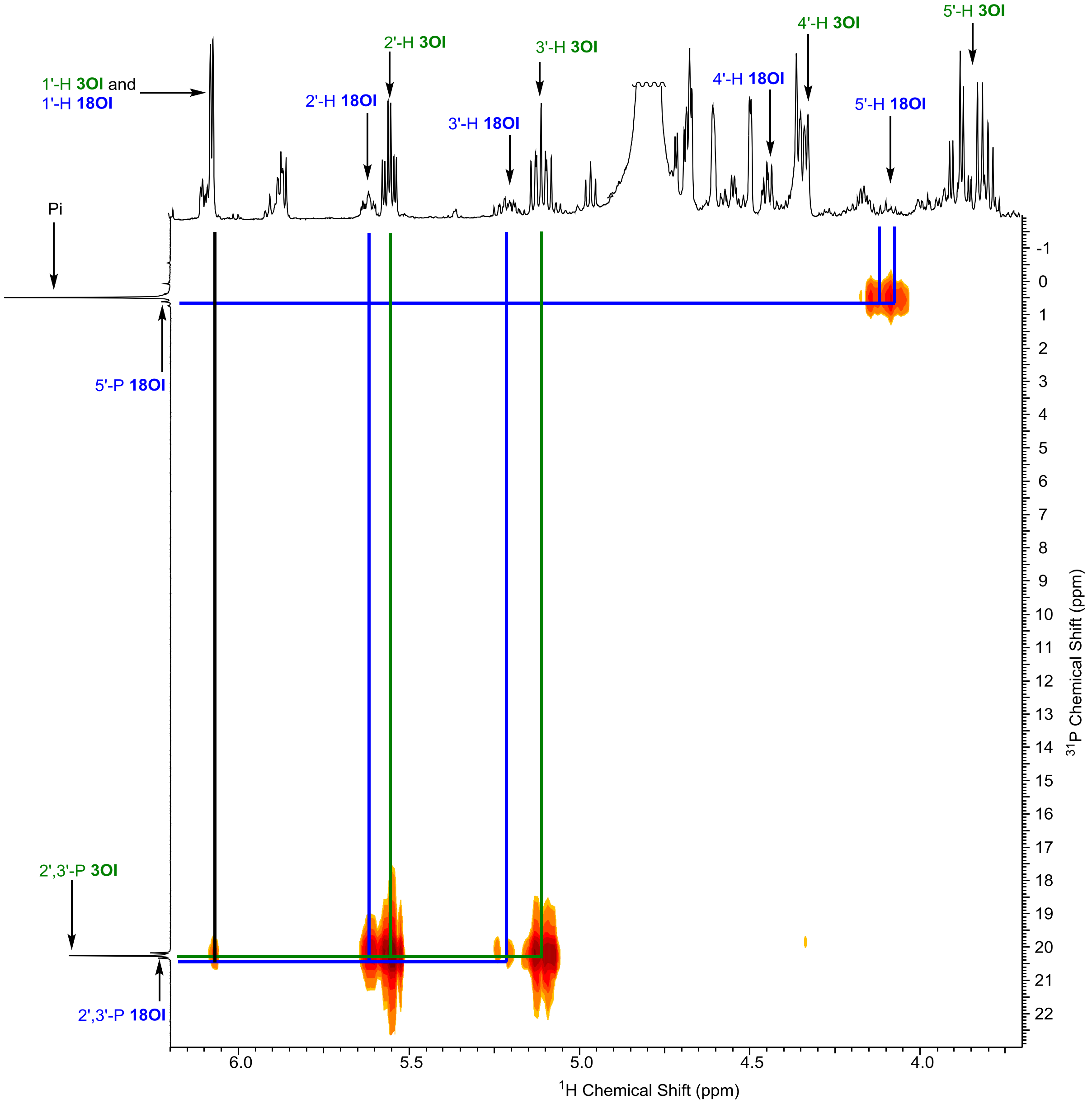

Supplementary Fig. 52. *- ^{1}H - ^{31}P HMBC NMR spectrum (400/162 MHz, D_2O, 3.7 – 6.2; 0.0 – 25.0 ppm) to show phosphorylation of 8,2'-O-anhydro-9-β-arabinofuranosyl-cycloinosine (**17I**) by method **II**.*

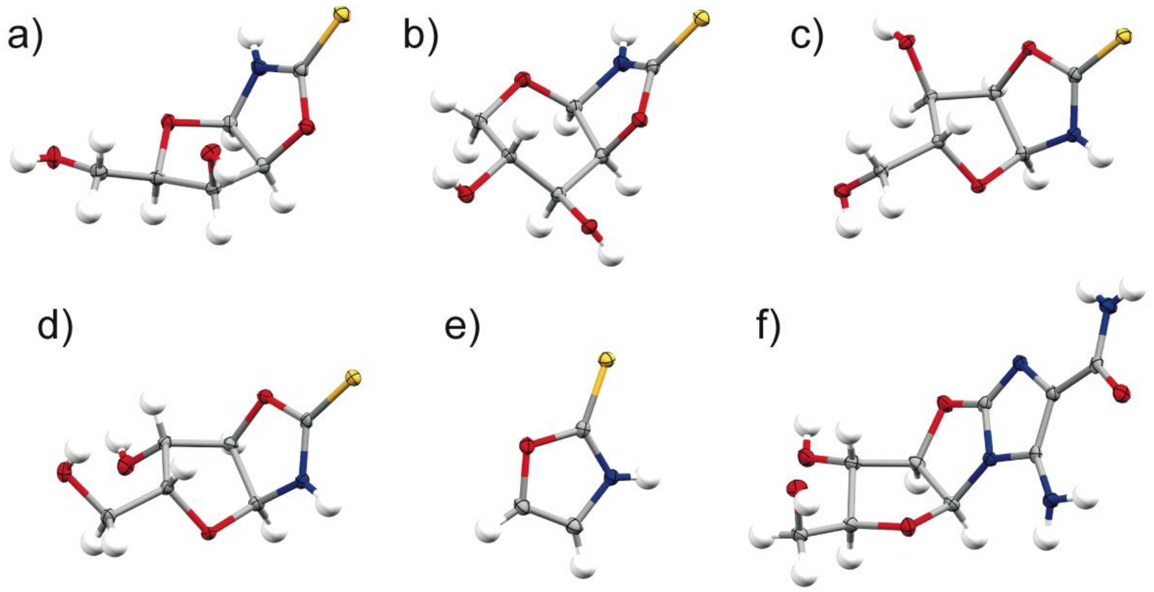

Supplementary Figure 53. *ORTEP plots of the molecular structures of a) lyxo-furano-**1a**, b) lyxo-pyrano-**1a**, c) ribo-**1a**, d) xylo-**1a**, e) **4b** and f) arabino-**16c** (L-enantiomer).*

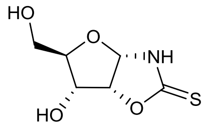

Ribofuranosyl oxazolidinone thione

ribo-**1a**

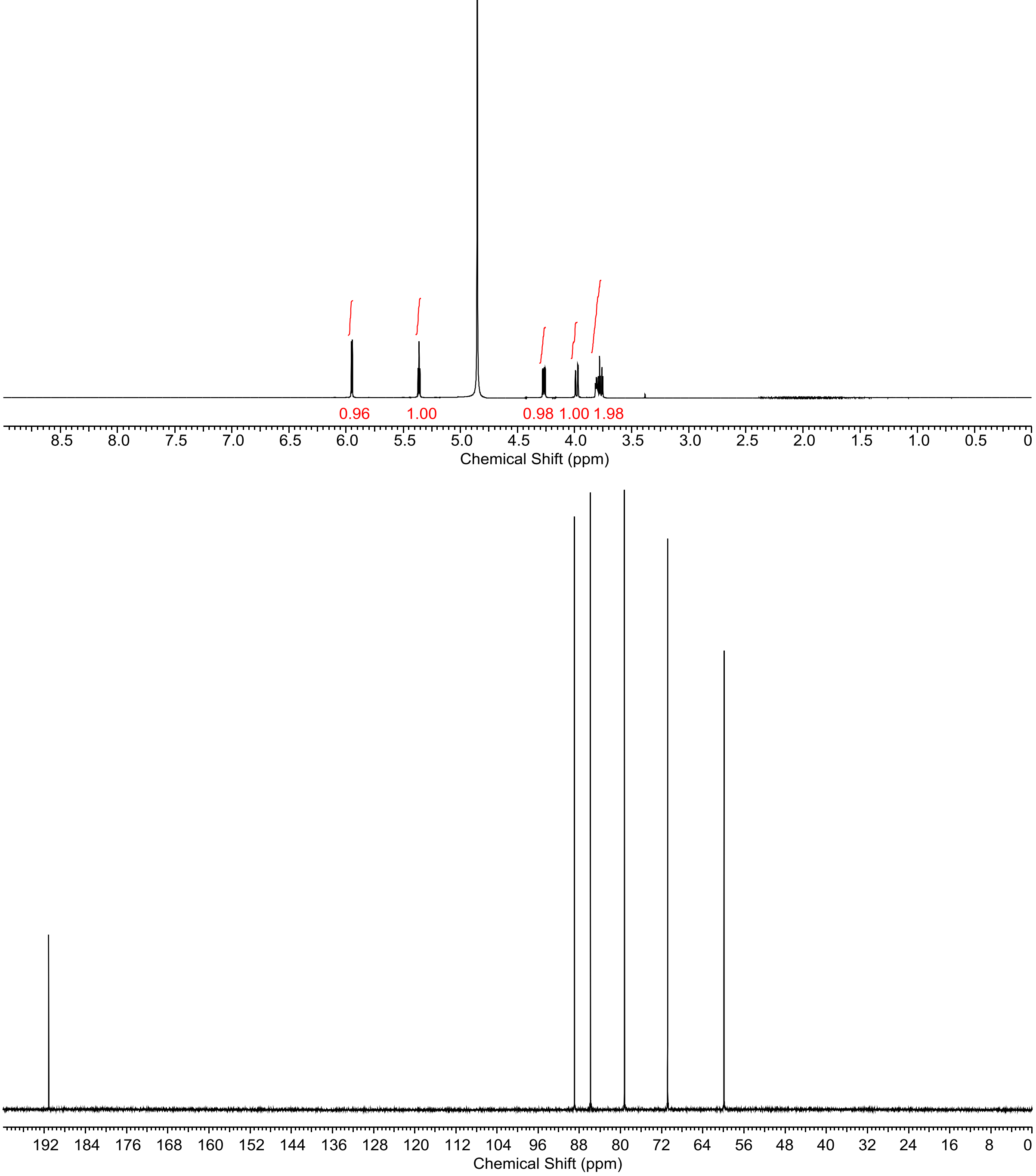

***Supplementary Fig. 54.** – ^{1}H NMR (600 MHz, D_2O, 0-9 ppm, Top) and ^{13}C NMR (151 MHz, D_2O, 0-200 ppm, Bottom) spectra of ribofuranosyl oxazolidinone thione (ribo-**1a**).*

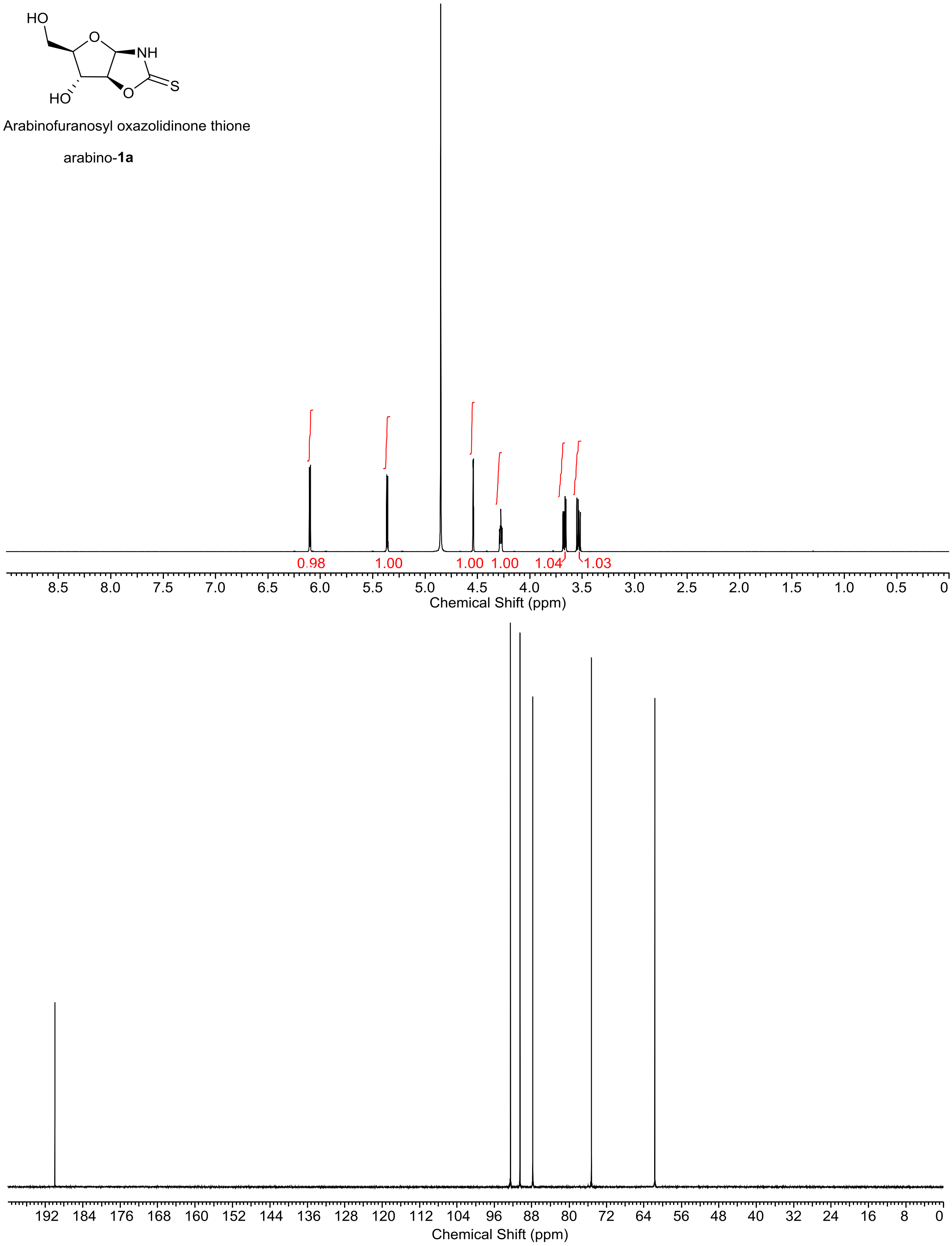

Supplementary Fig. 55. *– ^{1}H NMR (600 MHz, D_2O, 0-9 ppm, Top) and ^{13}C NMR (151 MHz, D_2O, 0-200 ppm, Bottom) spectra of arabinofuranosyl oxazolidinone thione (arabino-**1a**).*

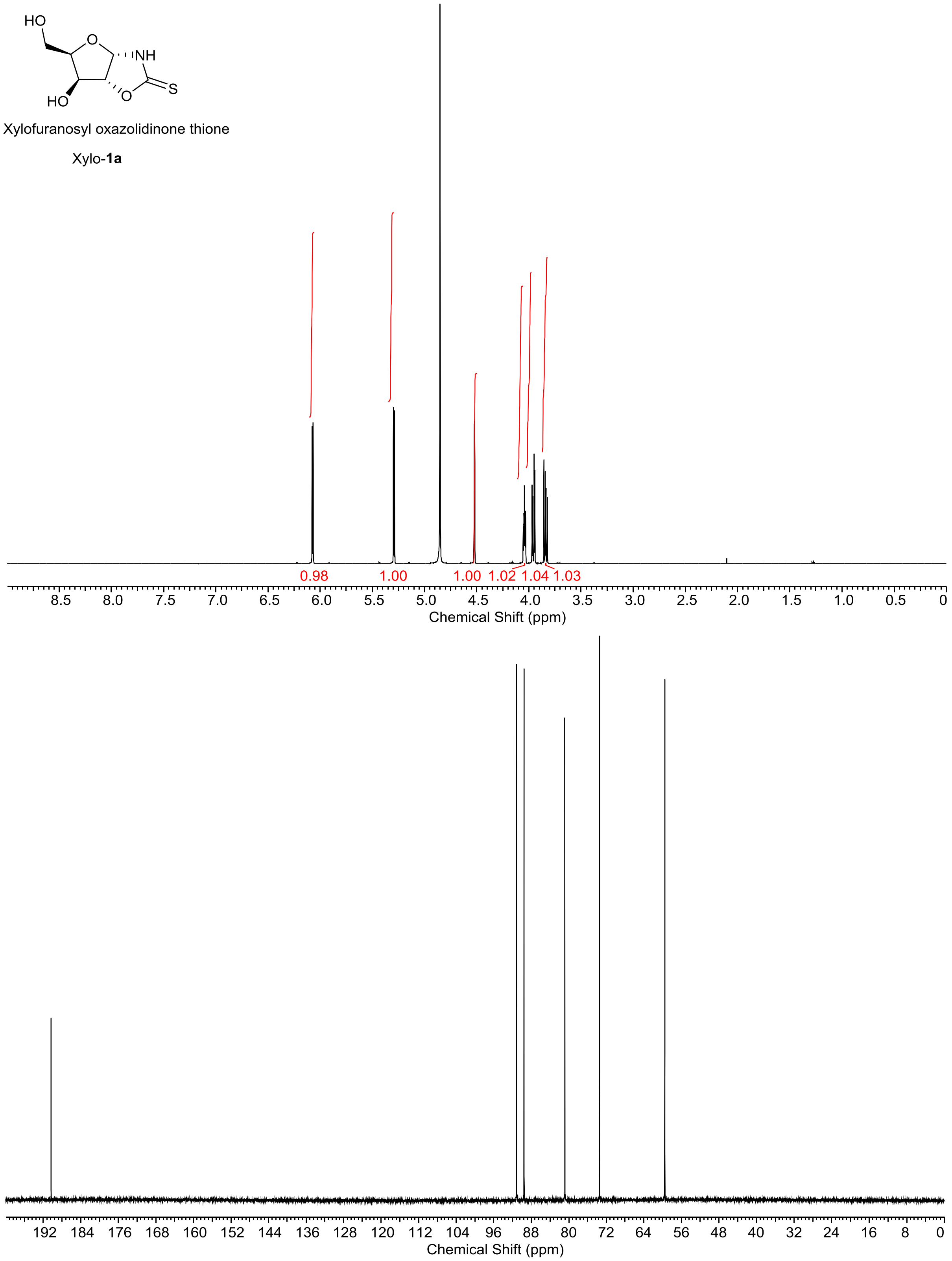

Supplementary Fig. 56. *– ^{1}H NMR (600 MHz, D_2O, 0-9 ppm, Top) and ^{13}C NMR (151 MHz, D_2O, 0-200 ppm, Bottom) spectra of xylofuranosyl oxazolidinone thione (xylo-**1a**).*

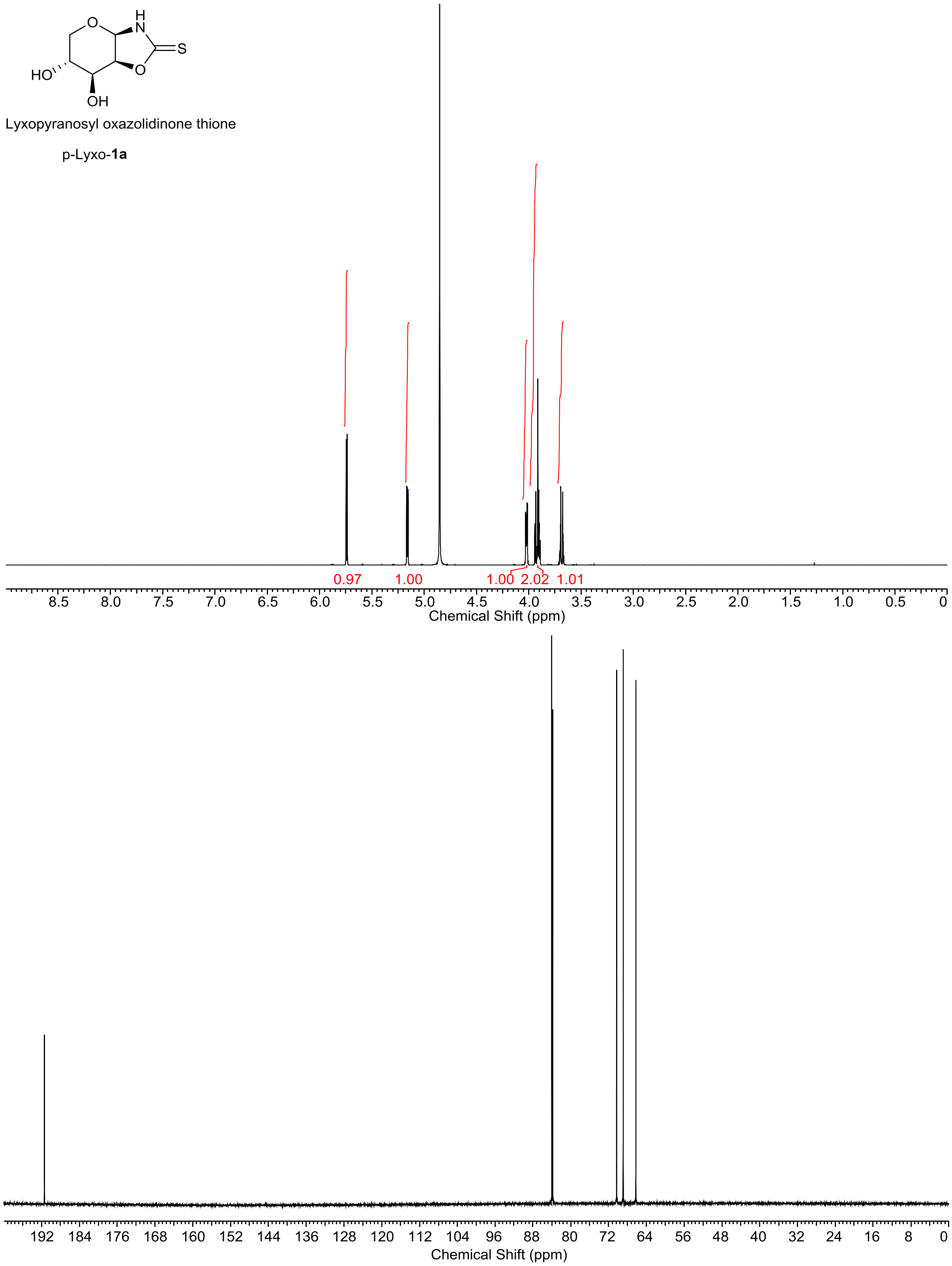

Supplementary Fig. 57. *– ^{1}H NMR (600 MHz, D_2O, 0-9 ppm, Top) and ^{13}C NMR (151 MHz, D_2O, 0-200 ppm, Bottom) spectra of lyxopyranosyl oxazolidinone thione (p-lyxo-**1a**).*

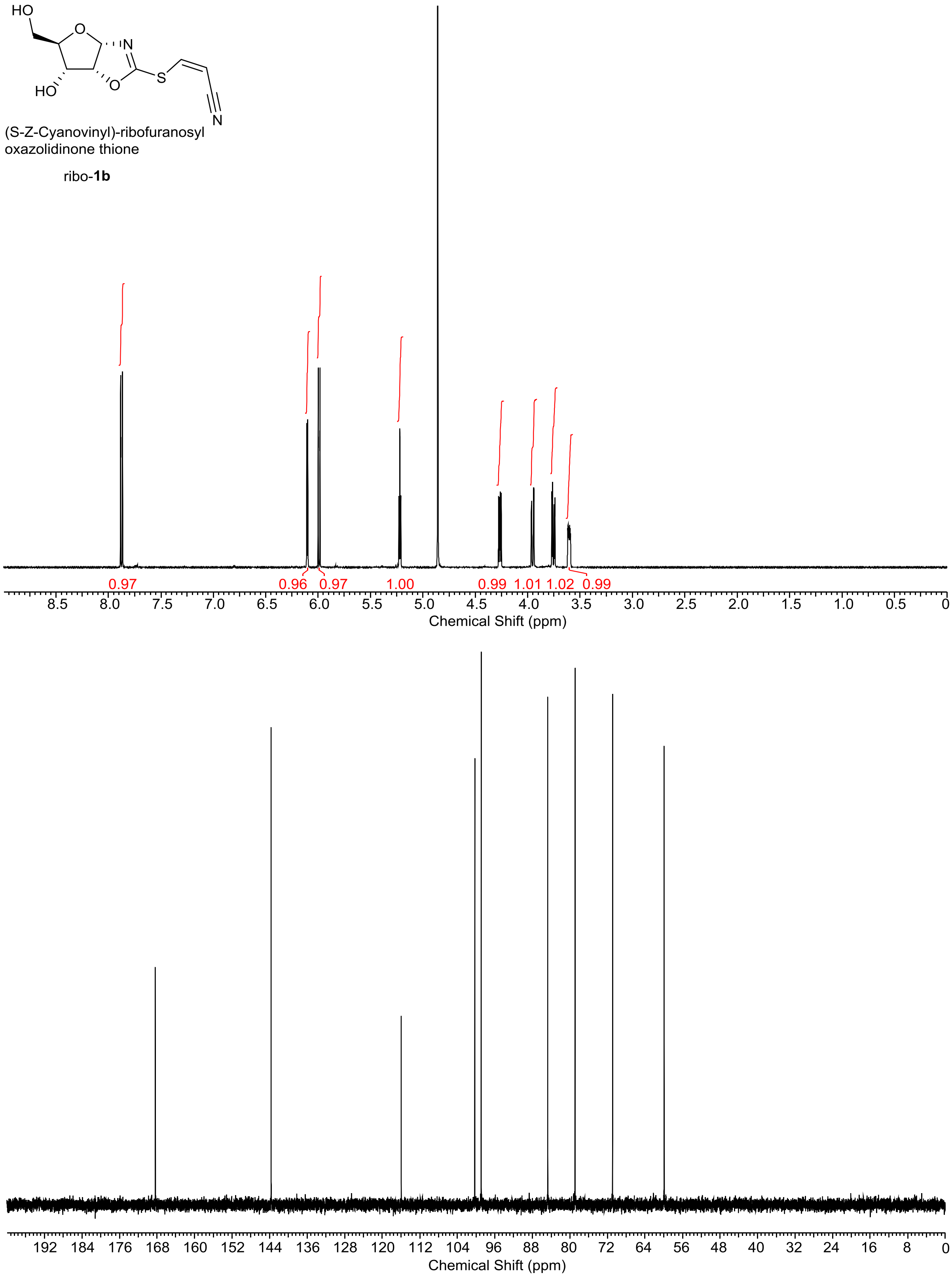

Supplementary Fig. 58. *– ^{1}H NMR (600 MHz, D_2O, 0-9 ppm, Top) and ^{13}C NMR (151 MHz, D_2O, 0-200 ppm, Bottom) spectra of (S-Z-Cyanovinyl)-ribofuranosyl oxazolidinone thione (ribo-**1b**)*

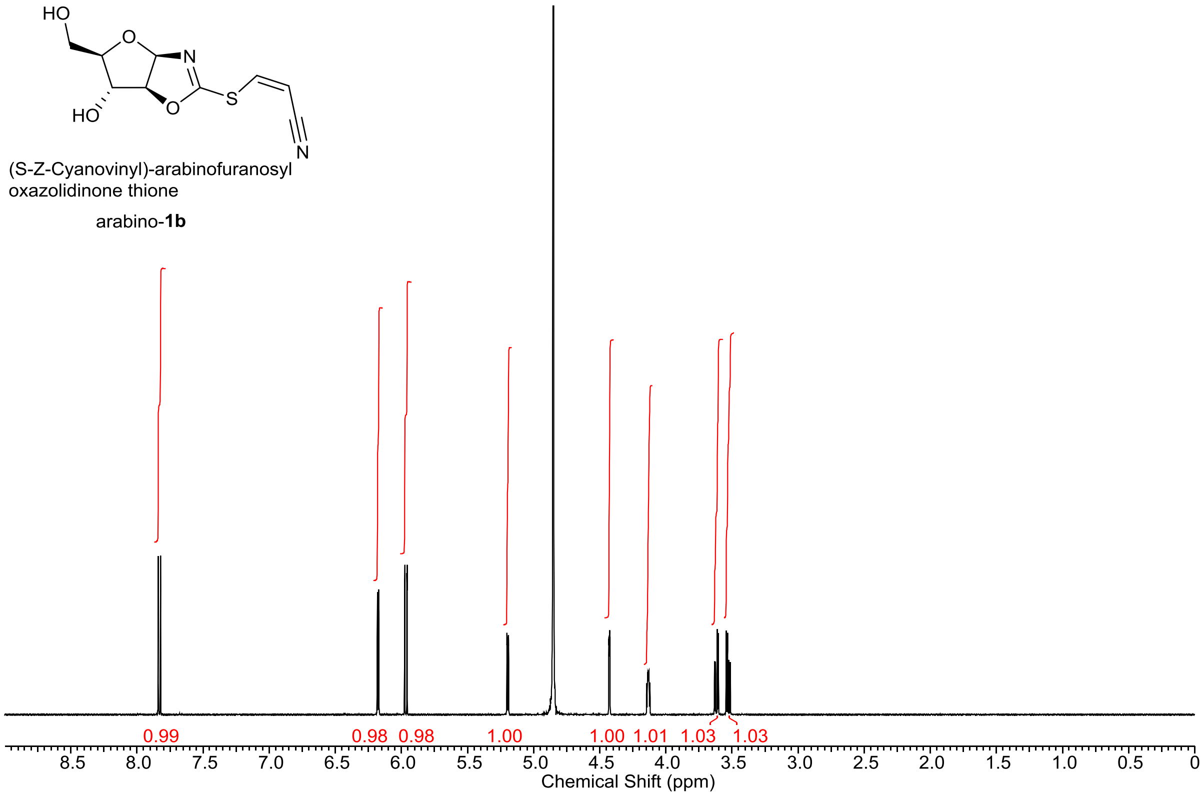

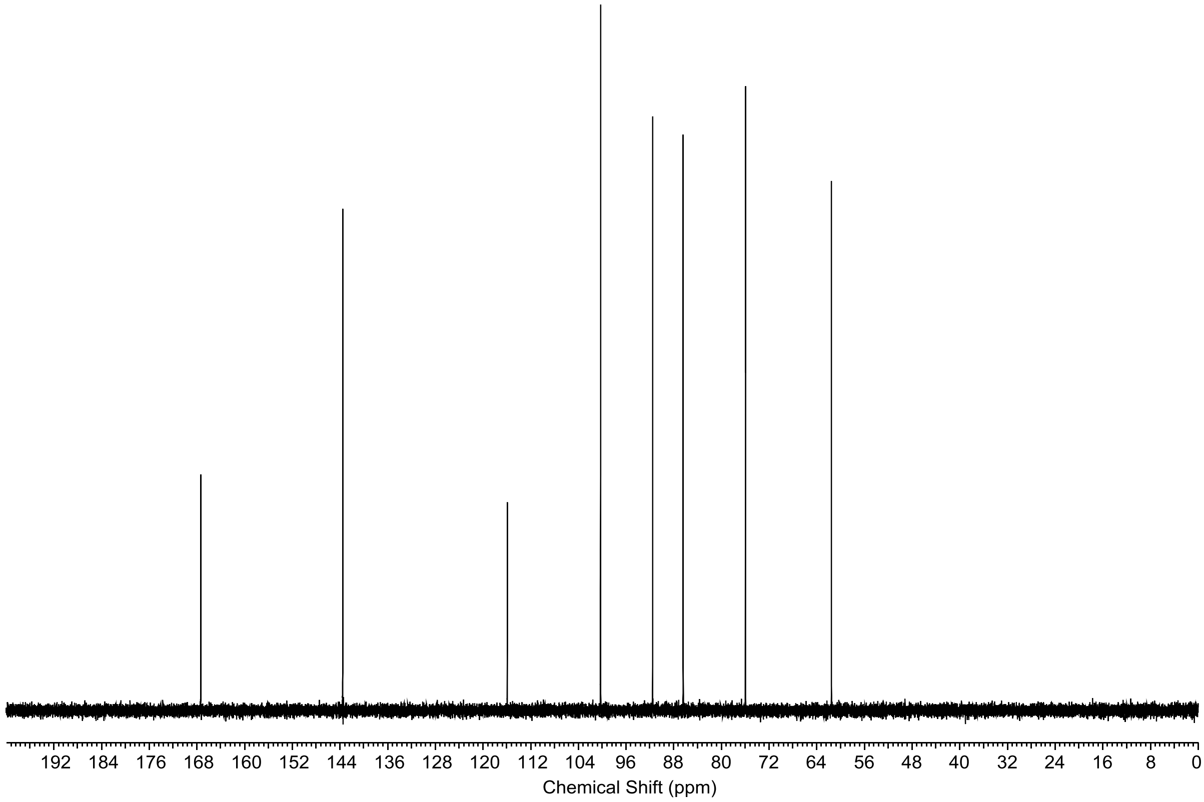

Supplementary Fig. 59. *– ^{1}H NMR (600 MHz, D_2O, 0-9 ppm, Top) and ^{13}C NMR (151 MHz, D_2O, 0-200 ppm, Bottom) spectra of (S-Z-Cyanovinyl)-arabinofuranosyl oxazolidinone thione (arabino-**1b**)*

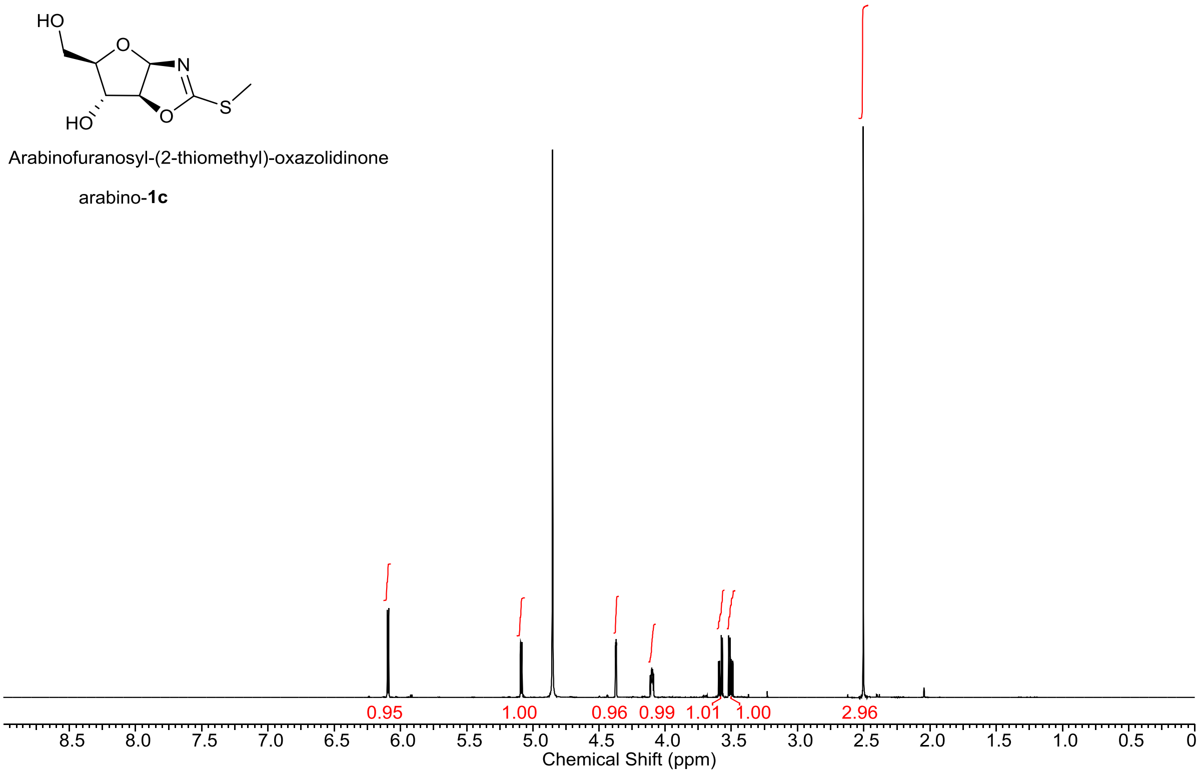

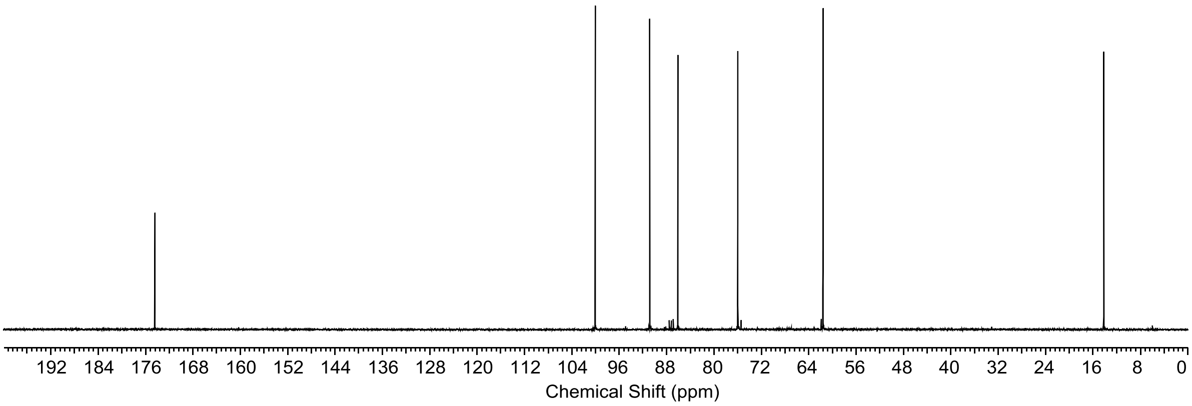

Supplementary Fig. 60. *– ^{1}H NMR (600 MHz, D_2O, 0-9 ppm, Top) and ^{13}C NMR (151 MHz, D_2O, 0-200 ppm, Bottom) spectra of arabinofuranosyl-(2-thiomethyl)-oxazolidinone (arabino-**1c**).*

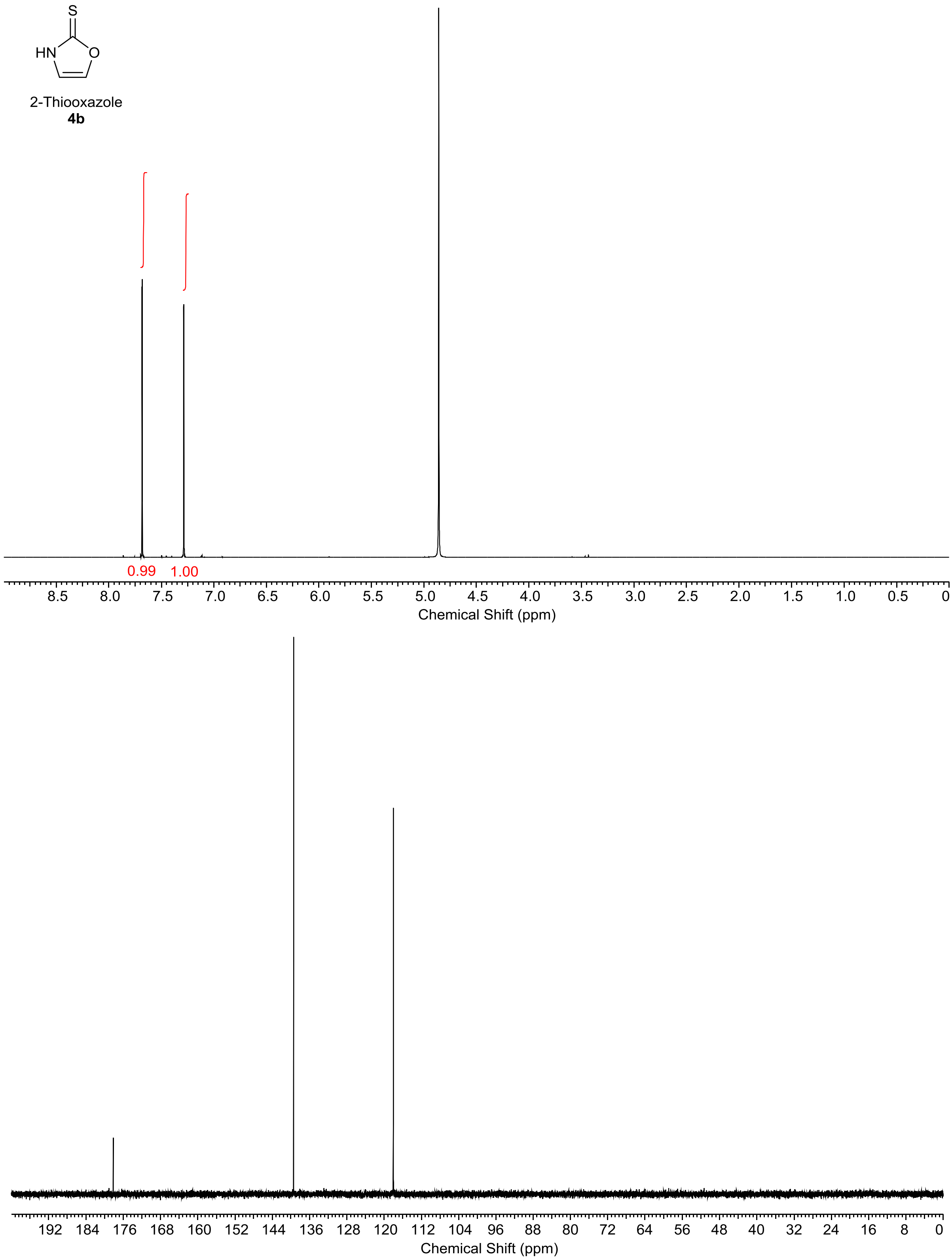

***Supplementary Fig. 61.** – ^{1}H NMR (600 MHz, D_2O, 0-9 ppm, Top) and ^{13}C NMR (151 MHz, D_2O, 0-200 ppm, Bottom) spectra of 2-thiooxazole (**4b**).*

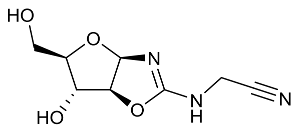

Arabinofuranosyl-N-acetonitrile-aminooxazoline

arabino-**10d**

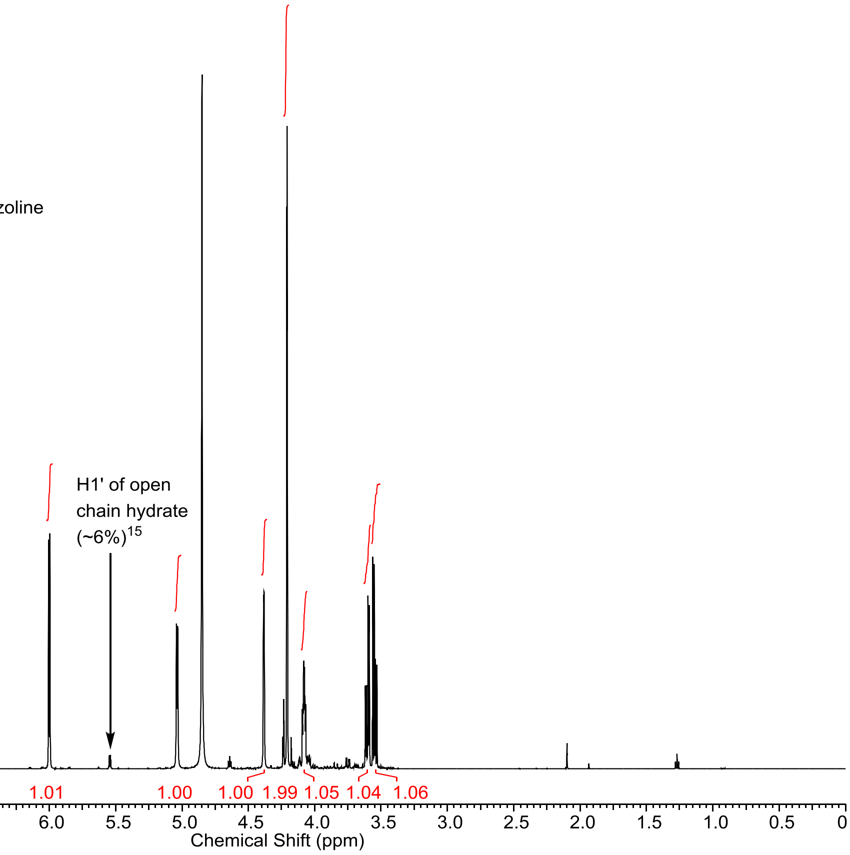

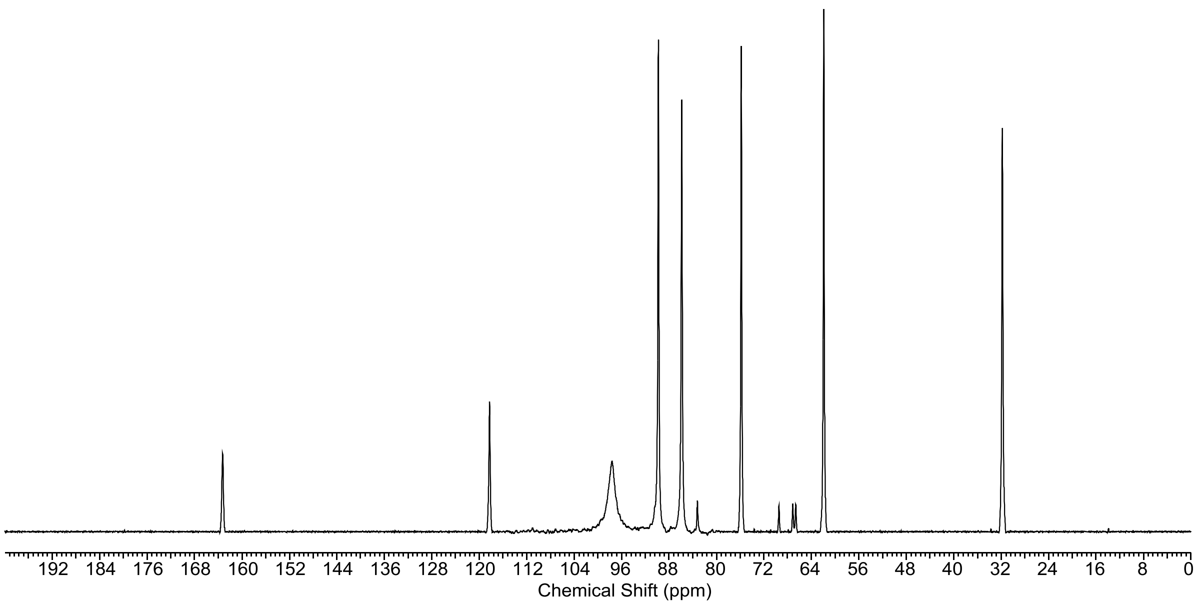

Supplementary Fig. 62. *– ^{1}H NMR (600 MHz, D_2O, 0-9 ppm, Top) and ^{13}C NMR (151 MHz, D_2O, 0-200 ppm, Bottom) spectra of arabinofuranosyl-N-acetonitrile-aminooxazoline (arabino-**10d**). Aminooxazolines (10) exist in solution in equilibrium with open-chain hydrates[15]; 6% of the open-chain hydrate of arabino-**10d** was observed in aqueous solution.*

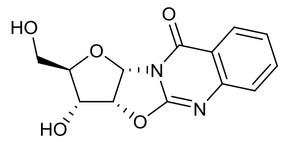

N3-Ribofuranosyl-2,2'-anhydroquinazolinedione

ribo-**12a**

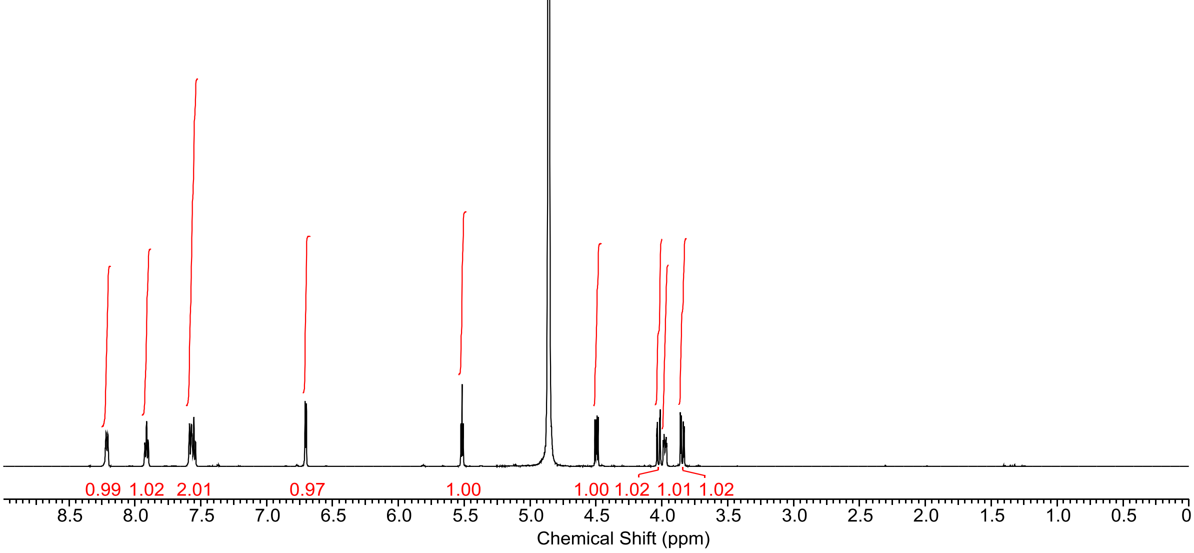

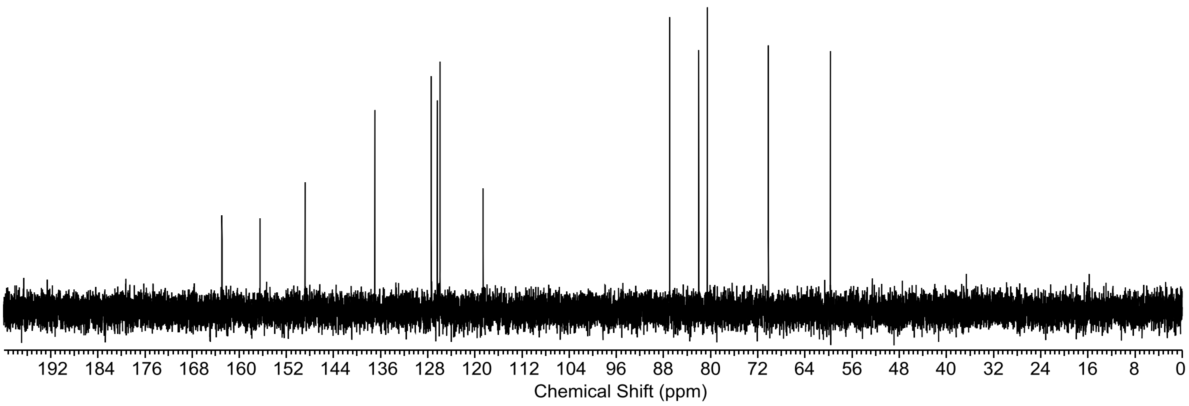

Supplementary Fig. 63. *– ^{1}H NMR (600 MHz, D_2O, 0-9 ppm, Top) and ^{13}C NMR (151 MHz, D_2O, 0-200 ppm, Bottom) spectra of N3-ribofuranosyl-2,2'-anhydroquinazolinedione (ribo-**12a**).*

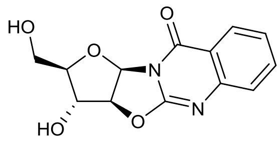

N3-Arabinofuranosyl-2,2'-anhydroquinazolinedione

arabino-**12a**

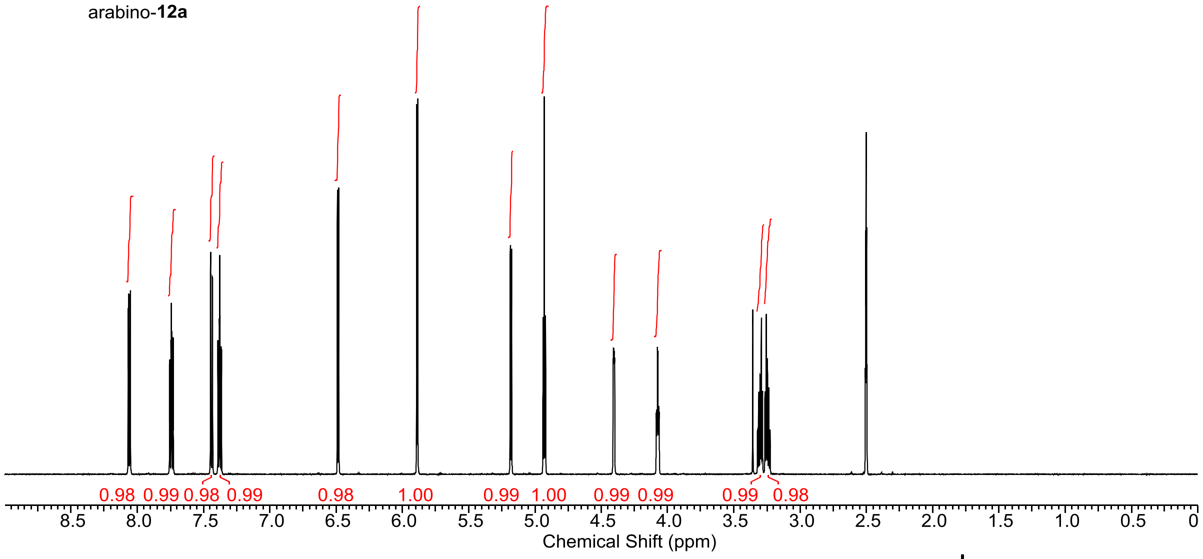

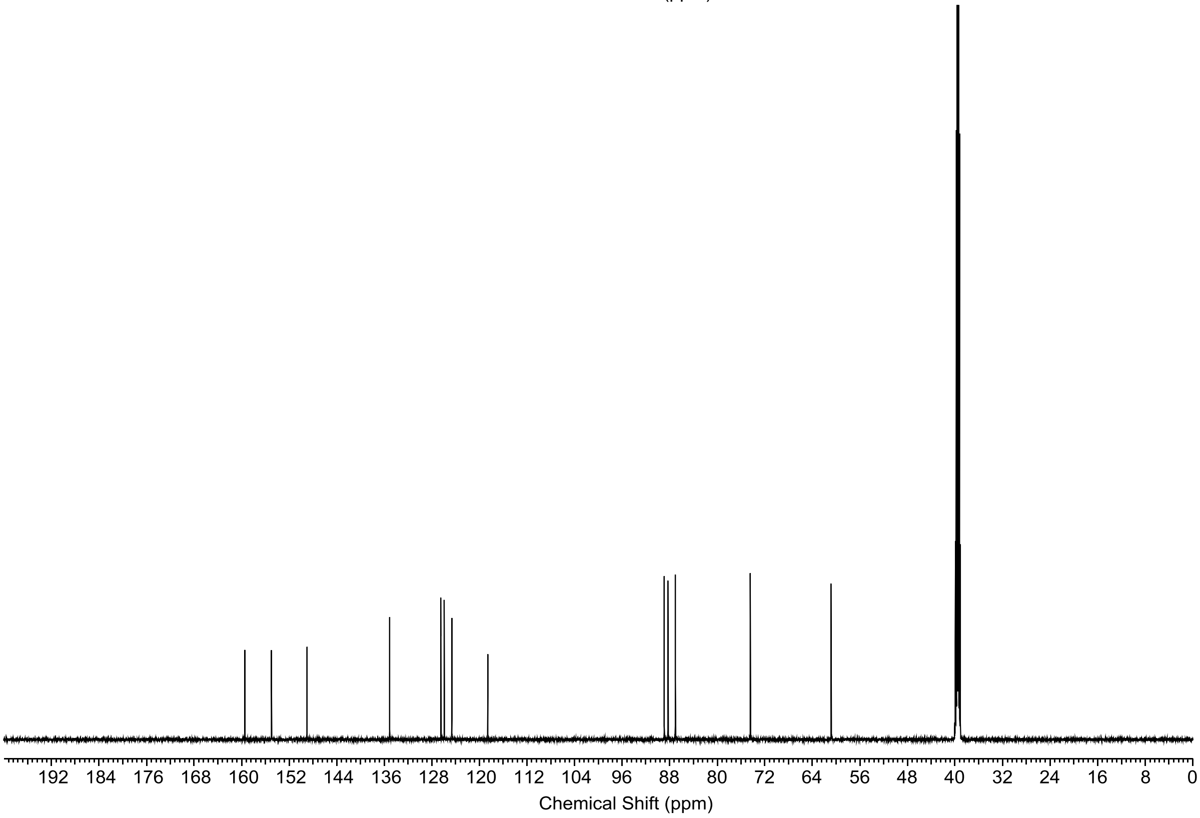

Supplementary Fig. 64. *– ^{1}H NMR (600 MHz, D_6-DMSO, 0-9 ppm, Top) and ^{13}C NMR (151 MHz, D_6-DMSO, 0-200 ppm, Bottom) spectra of N3-arabinofuranosyl-2,2'-anhydroquinazolinedione (arabino-**12a**).*

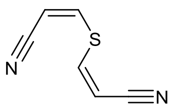

S-β-Dicyanovinyl sulfide
15

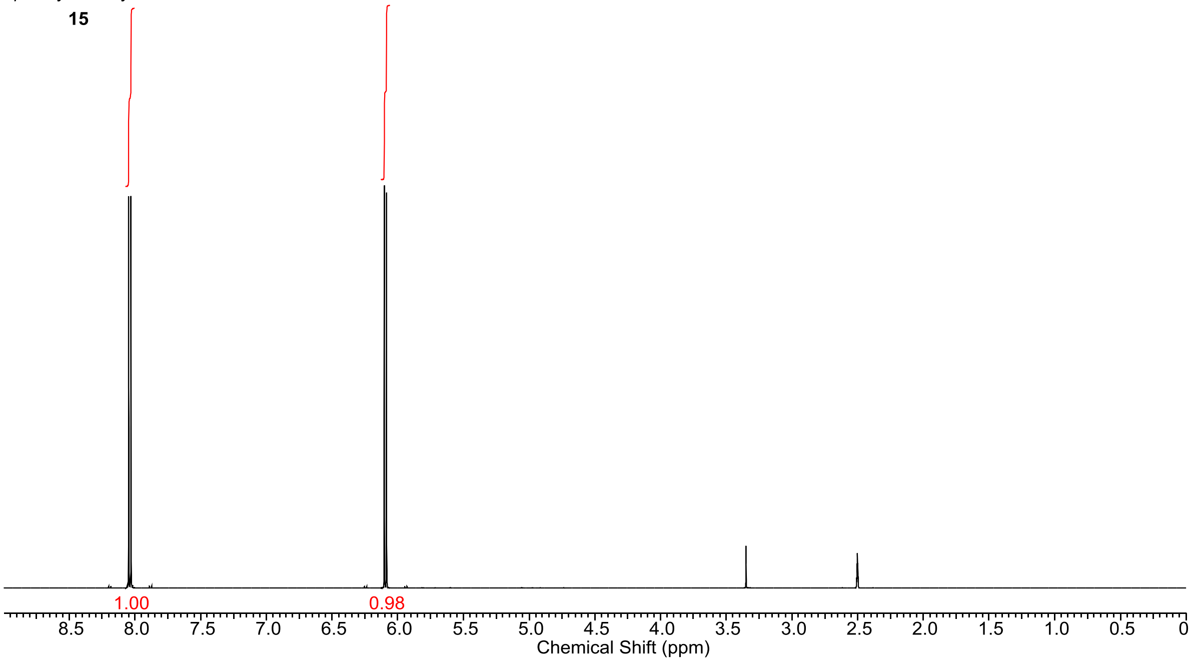

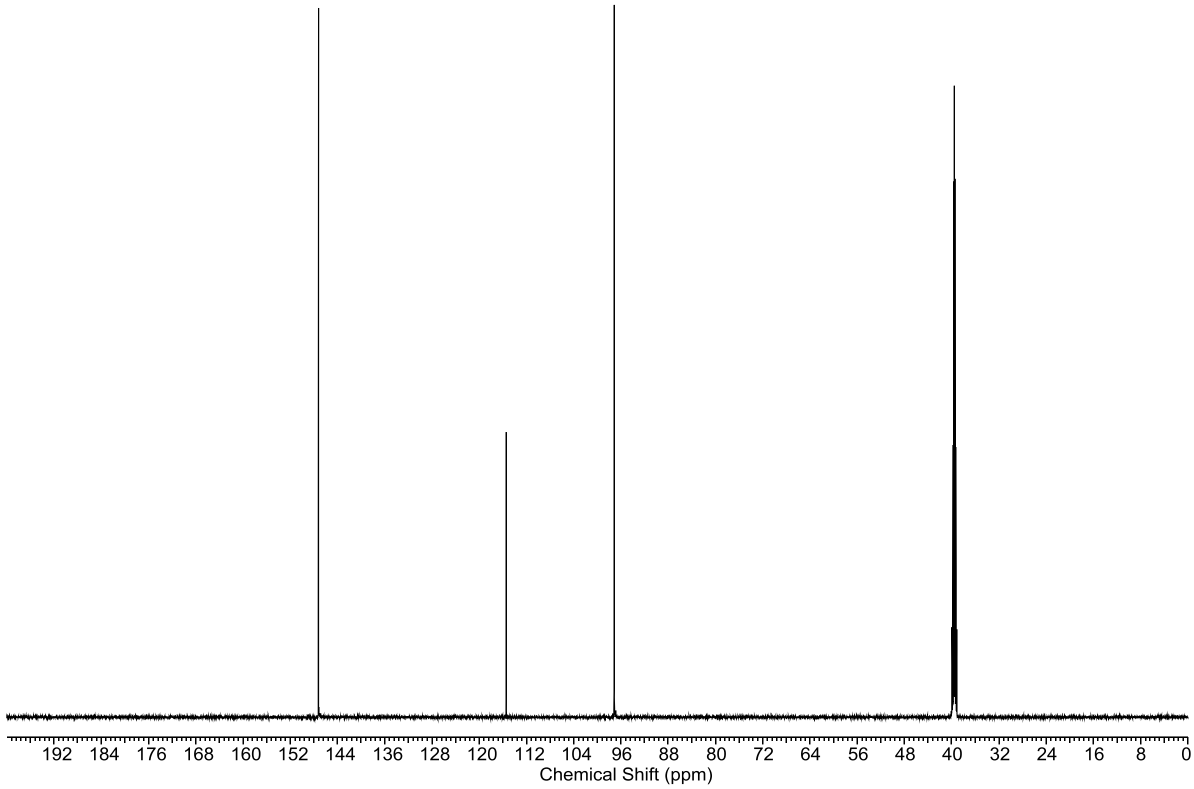

Supplementary Fig. 65. *– ^{1}H NMR (600 MHz, D_6-DMSO, 0-9 ppm, Top) and ^{13}C NMR (151 MHz, D_6-DMSO, 0-200 ppm, Bottom) spectra of S-β-dicyanovinyl sulfide (**15**).*

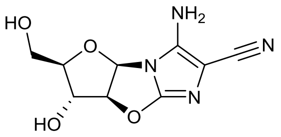

2,2'-Anhydro-5-aminoimidazole-4-carbonitrile-β-furanosylarabinoside

16b

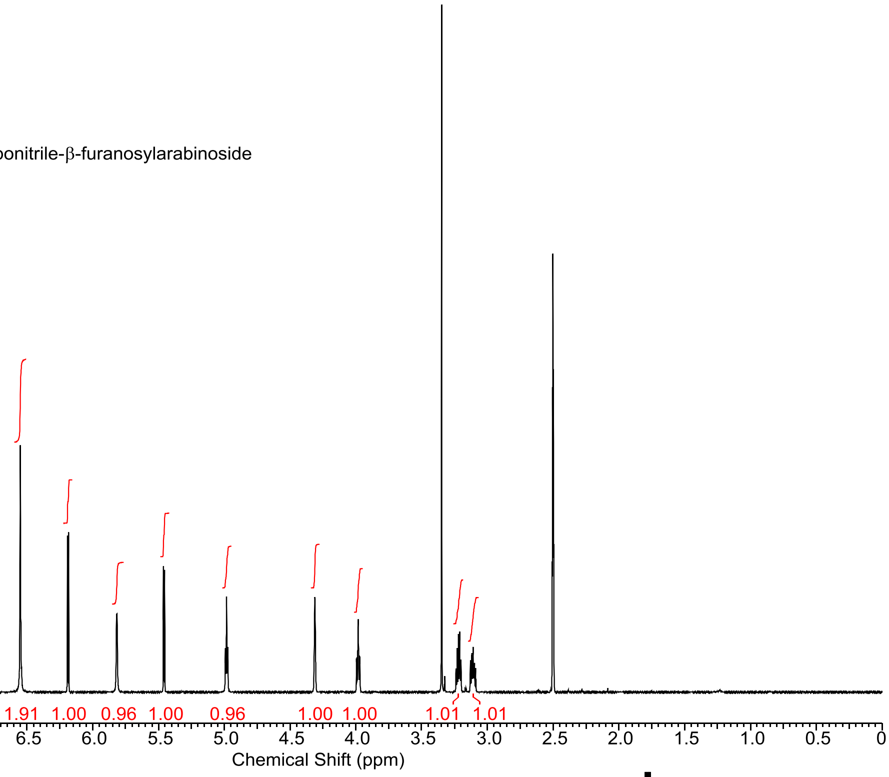

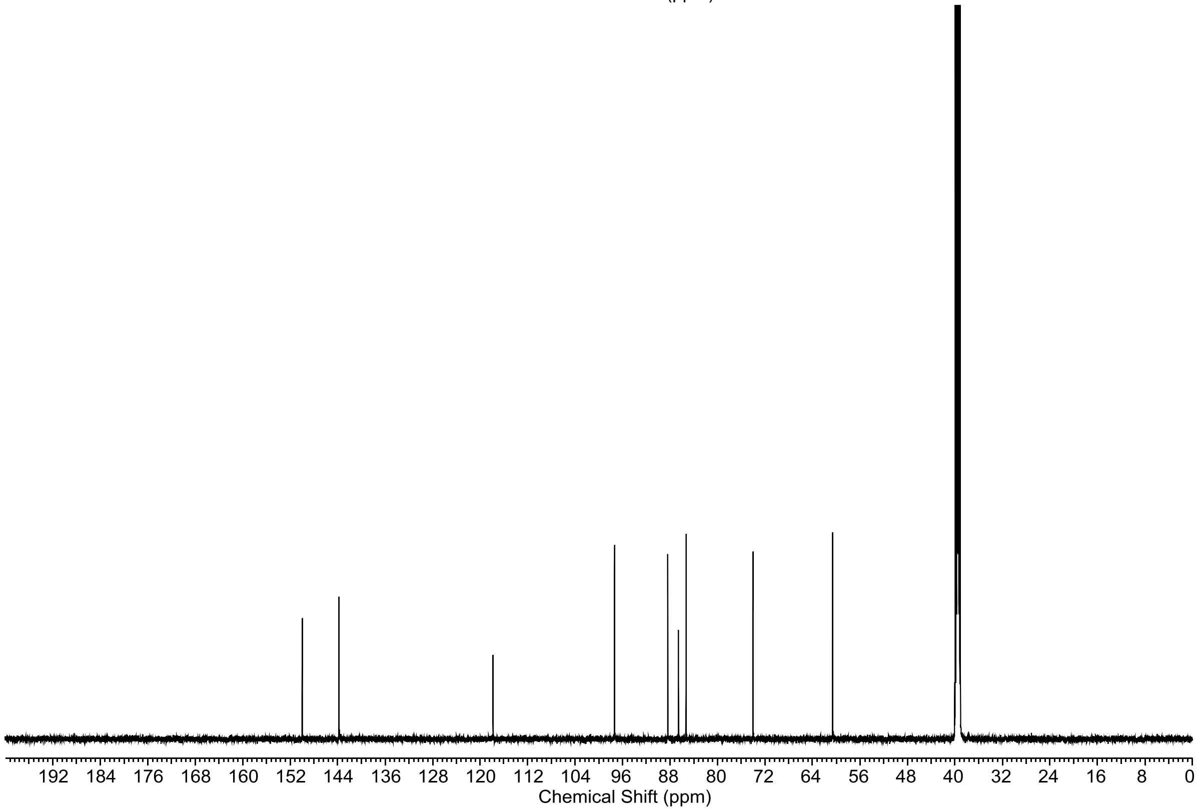

Supplementary Fig. 66. *– ^{1}H NMR (600 MHz, D_6-DMSO, 0-9 ppm, Top) and ^{13}C NMR (151 MHz, D_6-DMSO, 0-200 ppm, Bottom) spectra of 2,2'-anhydro-5-aminoimidazole-4-carbonitrile-β-furanosylarabinoside (**16b**).*

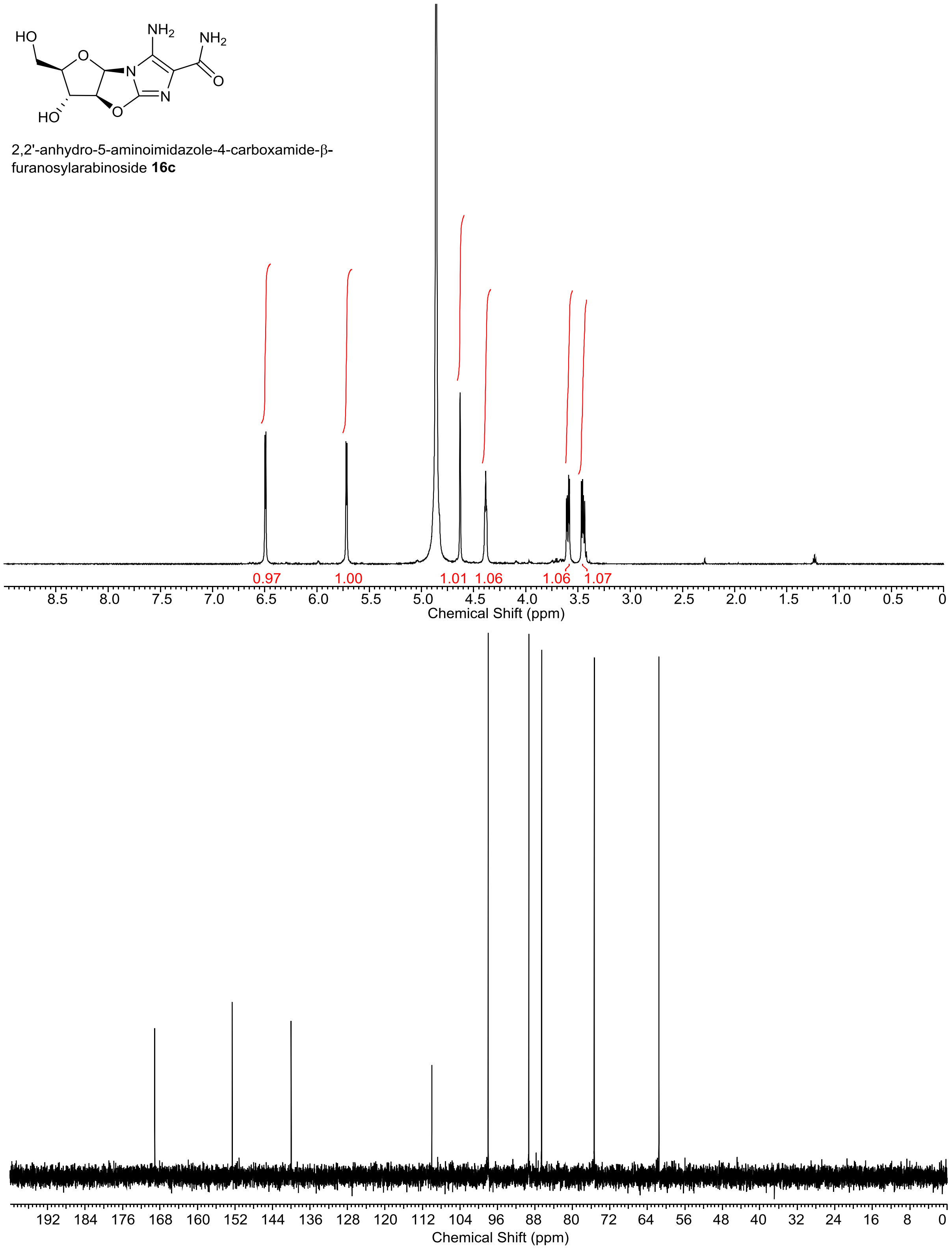

Supplementary Fig. 67. *– ^{1}H NMR (600 MHz, D_2O, 0-9 ppm, Top) and ^{13}C NMR (151 MHz, D_2O, 0-200 ppm, Bottom) spectra of 2,2'-anhydro-5-aminoimidazole-4-carboxamide-β-furanosylarabinoside (**16c**).*

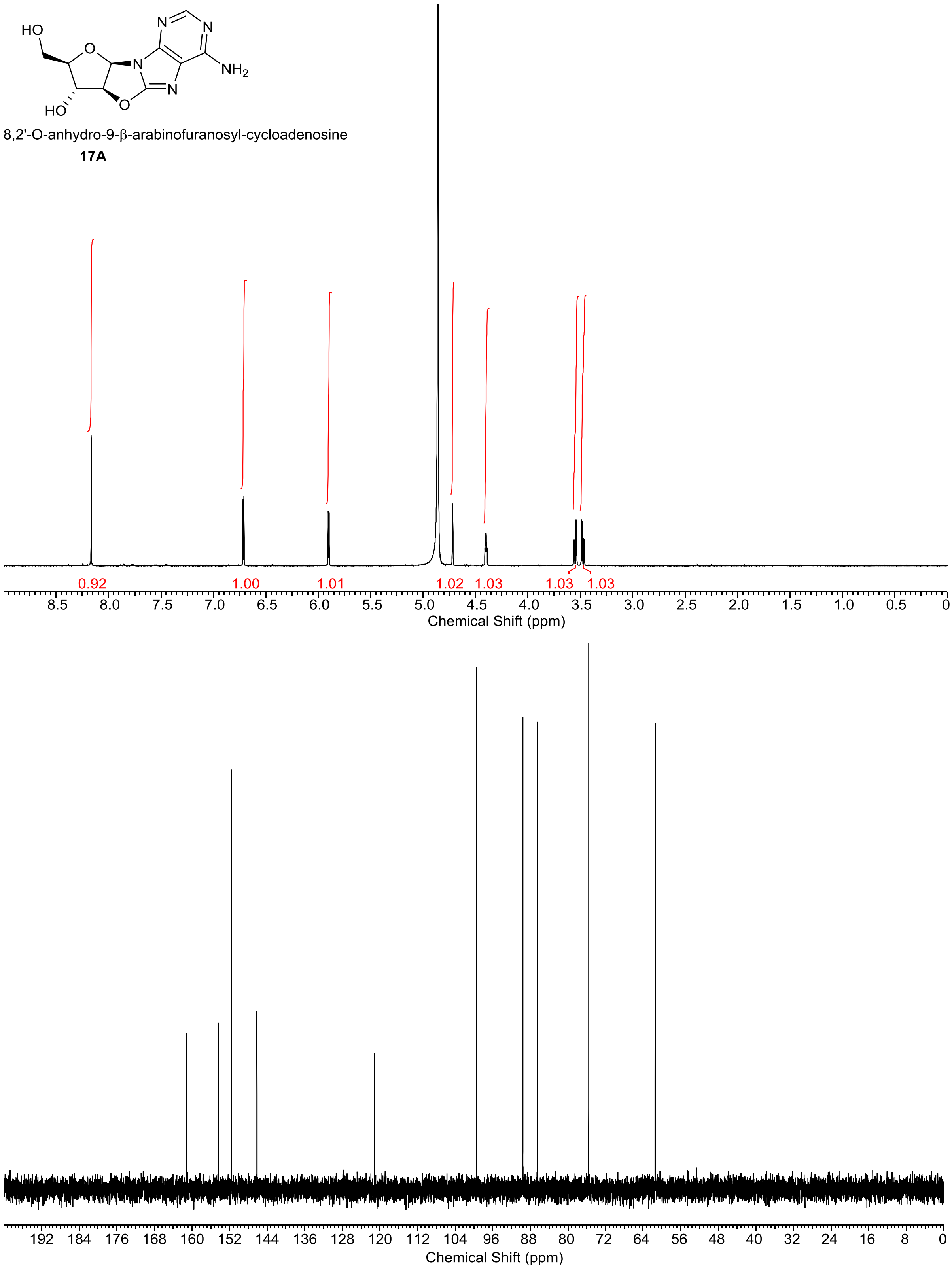

Supplementary Fig. 68. *– ^{1}H NMR (600 MHz, D_2O, 0-9 ppm, Top) and ^{13}C NMR (151 MHz, D_2O, 0-200 ppm, Bottom) spectra of 8,2'-O-anhydro-9-β-arabinofuranosyl-cycloadenosine (**17A**).*

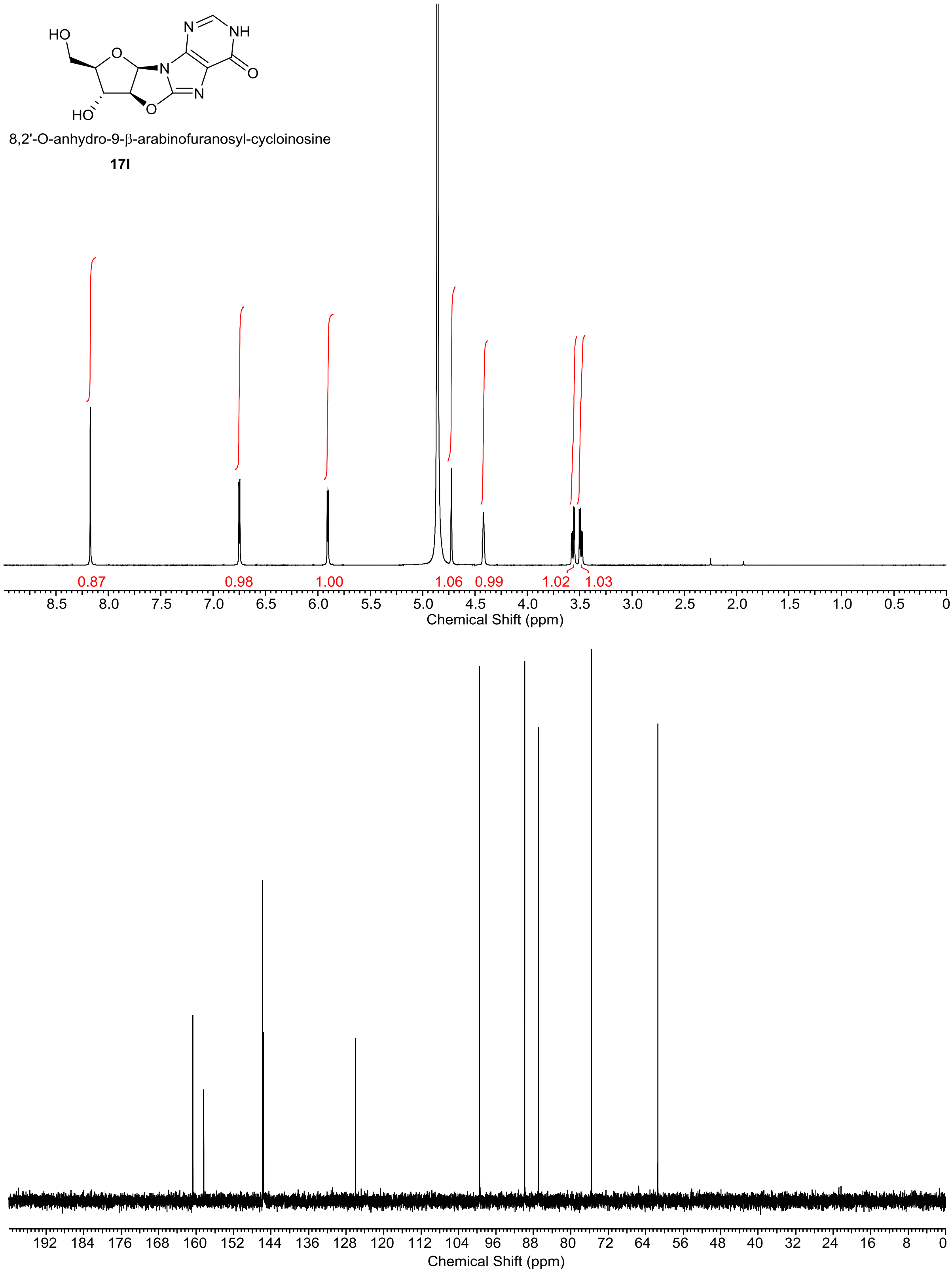

Supplementary Fig. 69. *– ^{1}H NMR (600 MHz, D_2O, 0-9 ppm, Top) and ^{13}C NMR (151 MHz, D_2O, 0-200 ppm, Bottom) spectra of* 8,2'-*O-anhydro-9-β-arabinofuranosyl-cycloinosine (**17l**).*

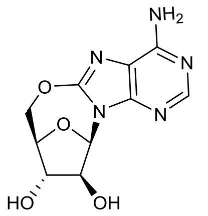

8,5'-O-anhydro-9-β-arabinofuranosyl-cycloadenosine

19

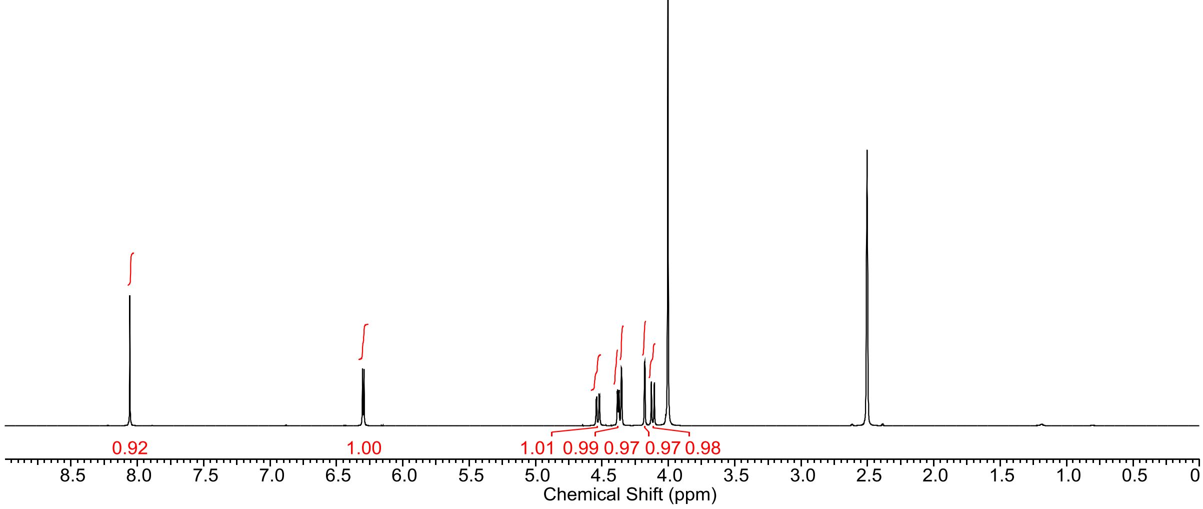

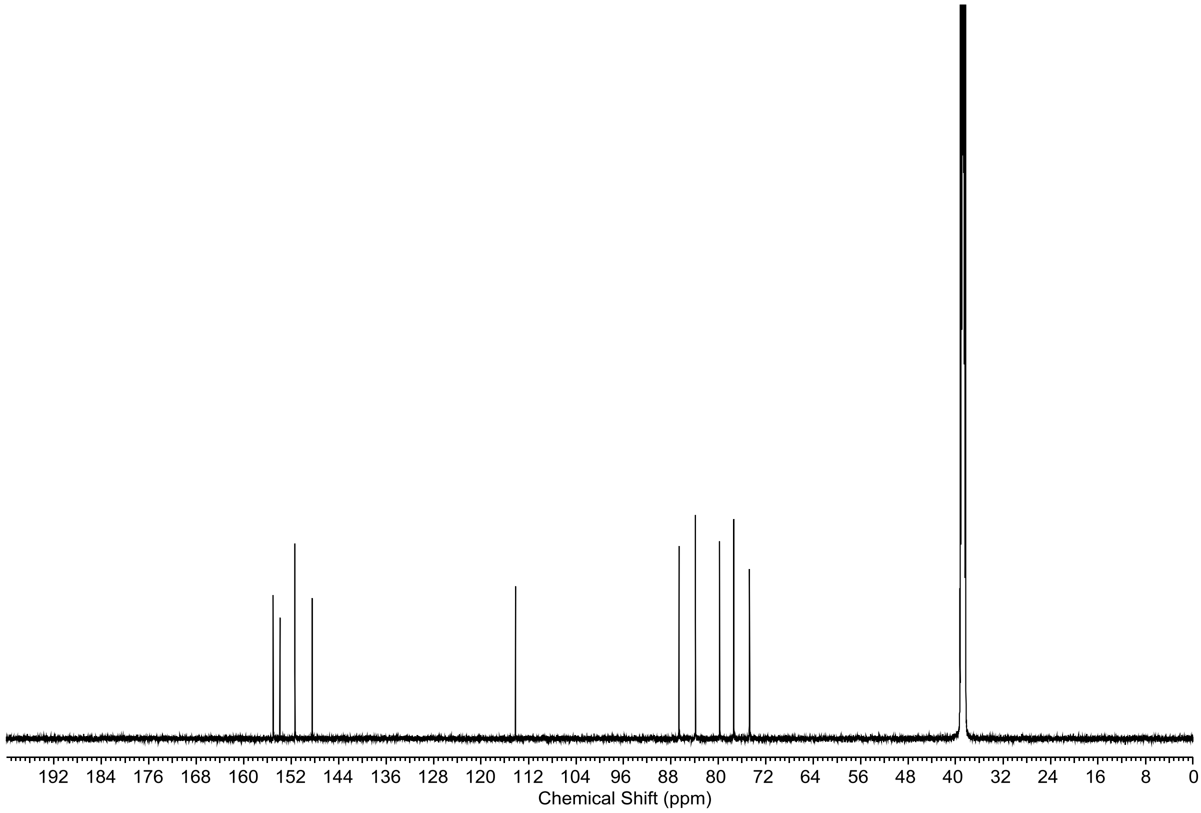

Supplementary Fig. 70. *– ^{1}H NMR (600 MHz, D_6-DMSO, 0-9 ppm, Top) and ^{13}C NMR (151 MHz, D_6-DMSO, 0-200 ppm, Bottom) spectra of 8,5'-O-anhydro-9-β-arabinofuranosyl-cycloadenosine (**19**).*

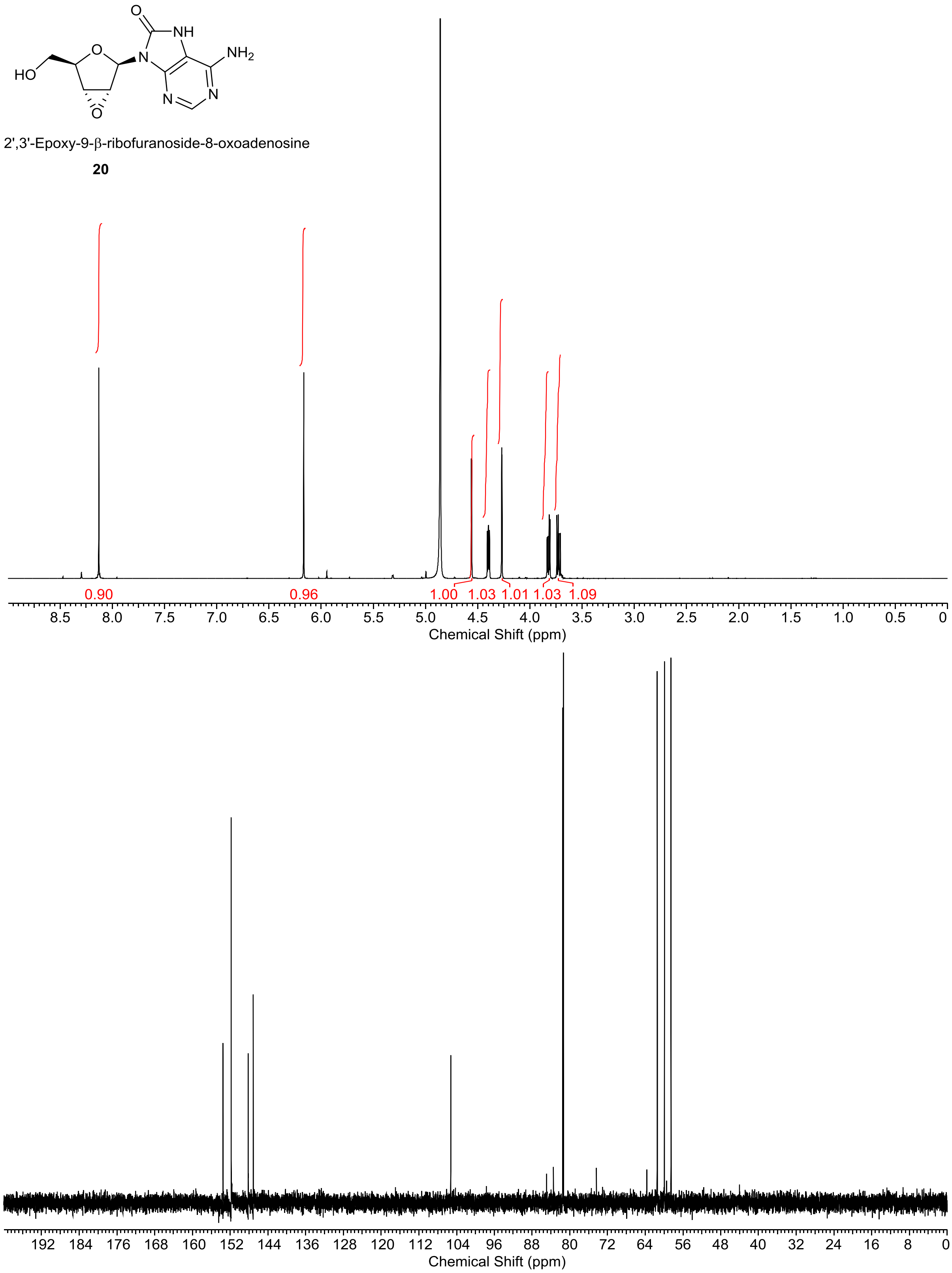

***Supplementary Fig. 71.** – ^{1}H NMR (600 MHz, D_2O, 0-9 ppm, Top) and ^{13}C NMR (151 MHz, D_2O, 0-200 ppm, Bottom) spectra of 2',3'-epoxy-9-β-ribofuranoside-8-oxoadenosine (**20**).*

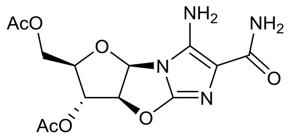

3',5'-Bisacetoxy-2,2'-anhydro-5-aminoimidazole-4-carboxamide-β-furanosylarabinoside

25

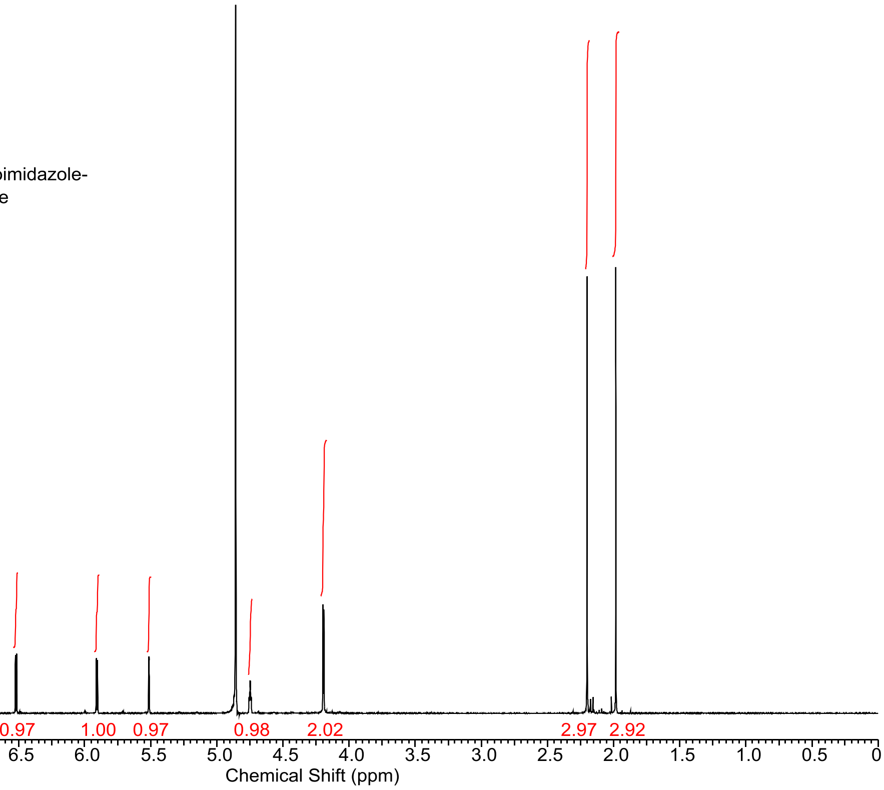

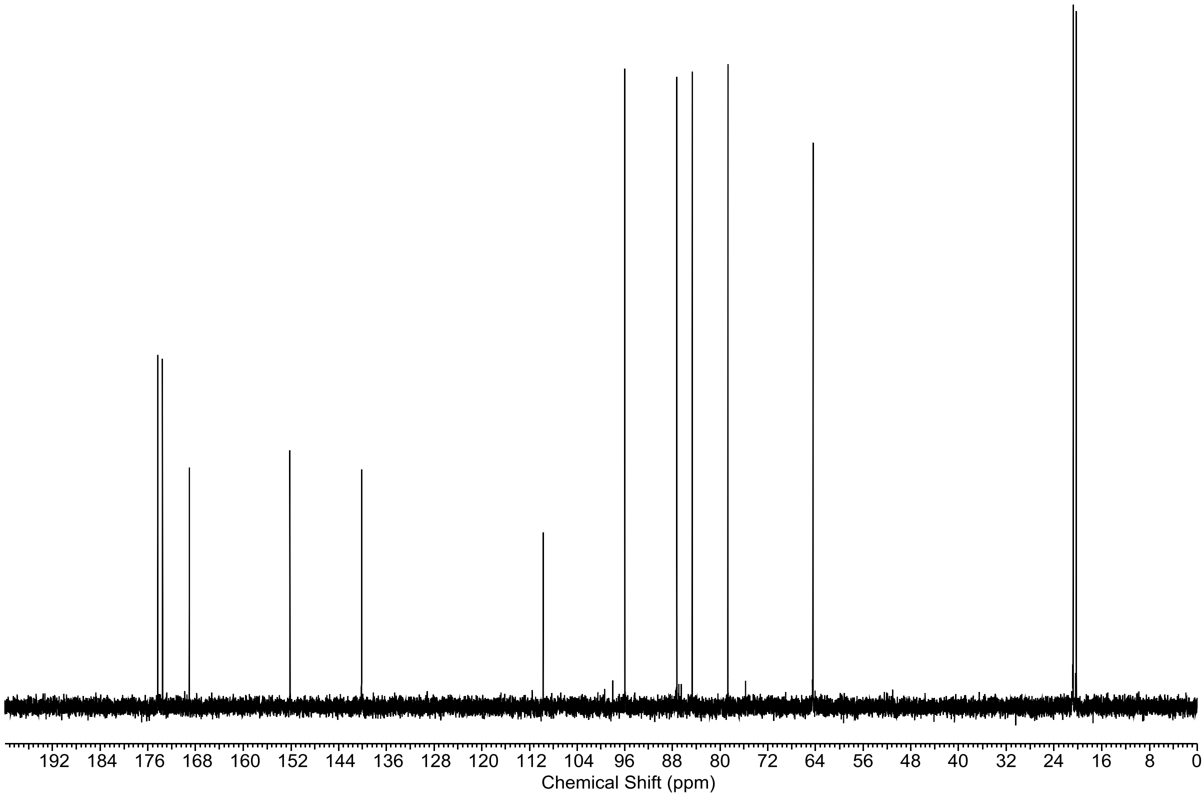

Supplementary Fig. 72. *– ^{1}H NMR (600 MHz, D_2O, 0-9 ppm, Top) and ^{13}C NMR (151 MHz, D_2O, 0-200 ppm, Bottom) spectra of 3',5'-bisacetoxy-2,2'-anhydro-5-aminoimidazole-4-carboxamide-β-furanosylarabinoside (**25**).*

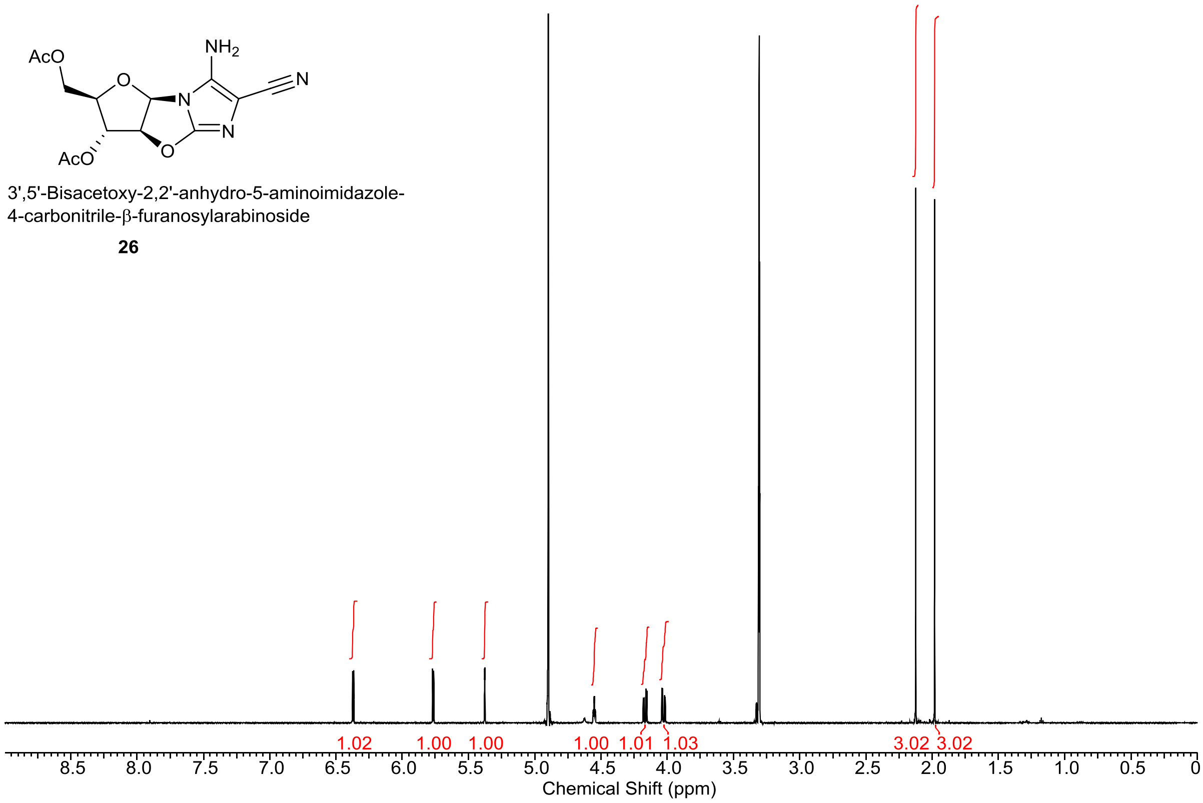

3',5'-Bisacetoxy-2,2'-anhydro-5-aminoimidazole-4-carbonitrile-β-furanosylarabinoside

26

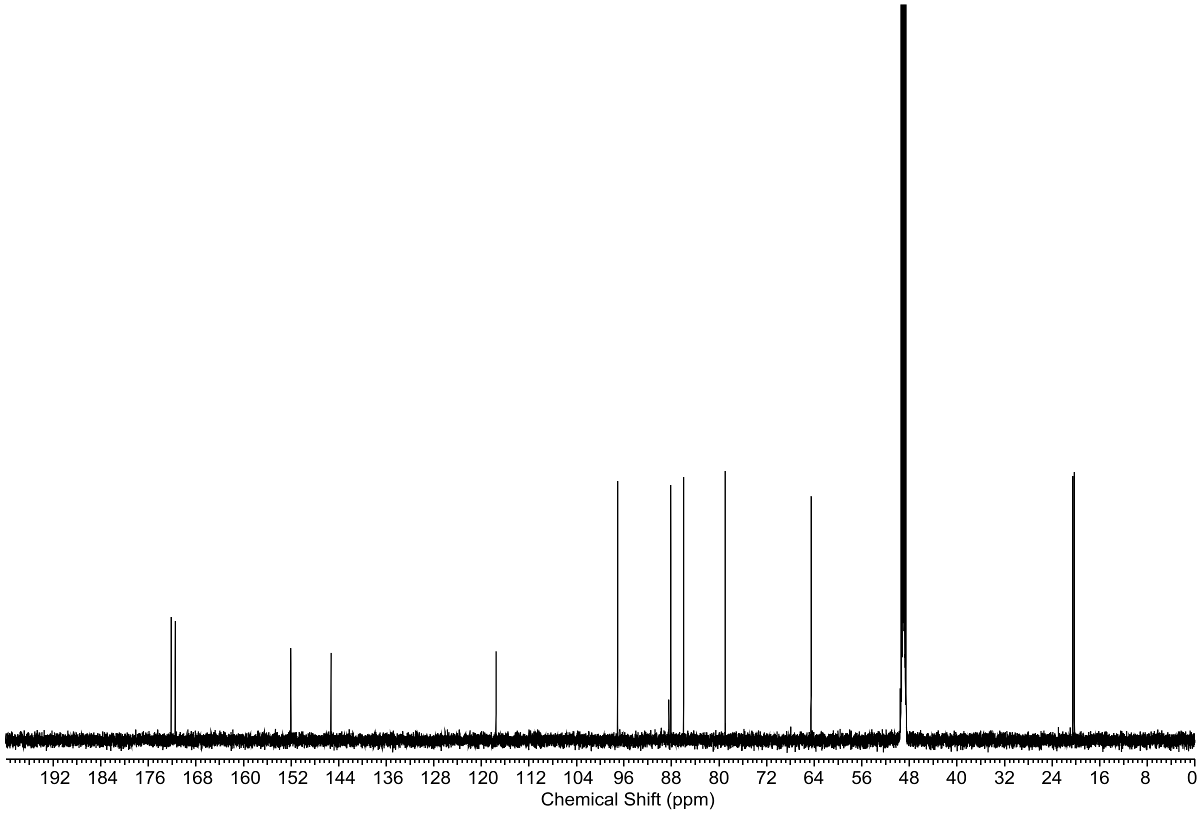

Supplementary Fig. 73. *– ^{1}H NMR (600 MHz, D_4-MeOD, 0-9 ppm, Top) and ^{13}C NMR (151 MHz, D_4-MeOD, 0-200 ppm, Bottom) spectra of 3',5'-bisacetoxy-2,2'-anhydro-5-aminoimidazole-4-carbonitrile-β-furanosylarabinoside (**26**).*

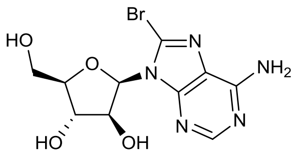

8-Bromoadenine-9-β-arabinofuranoside

27

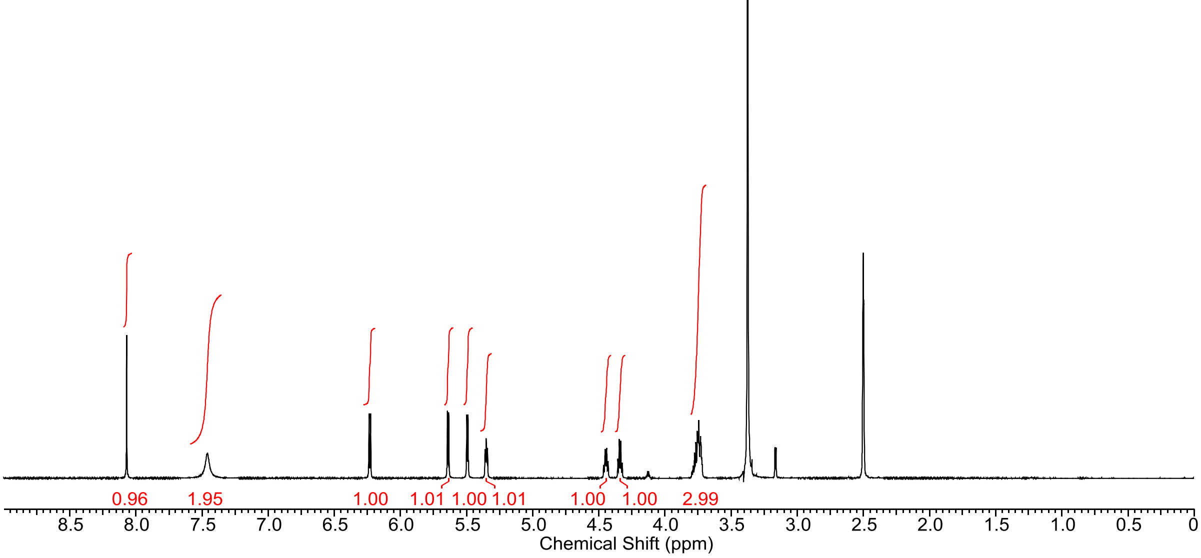

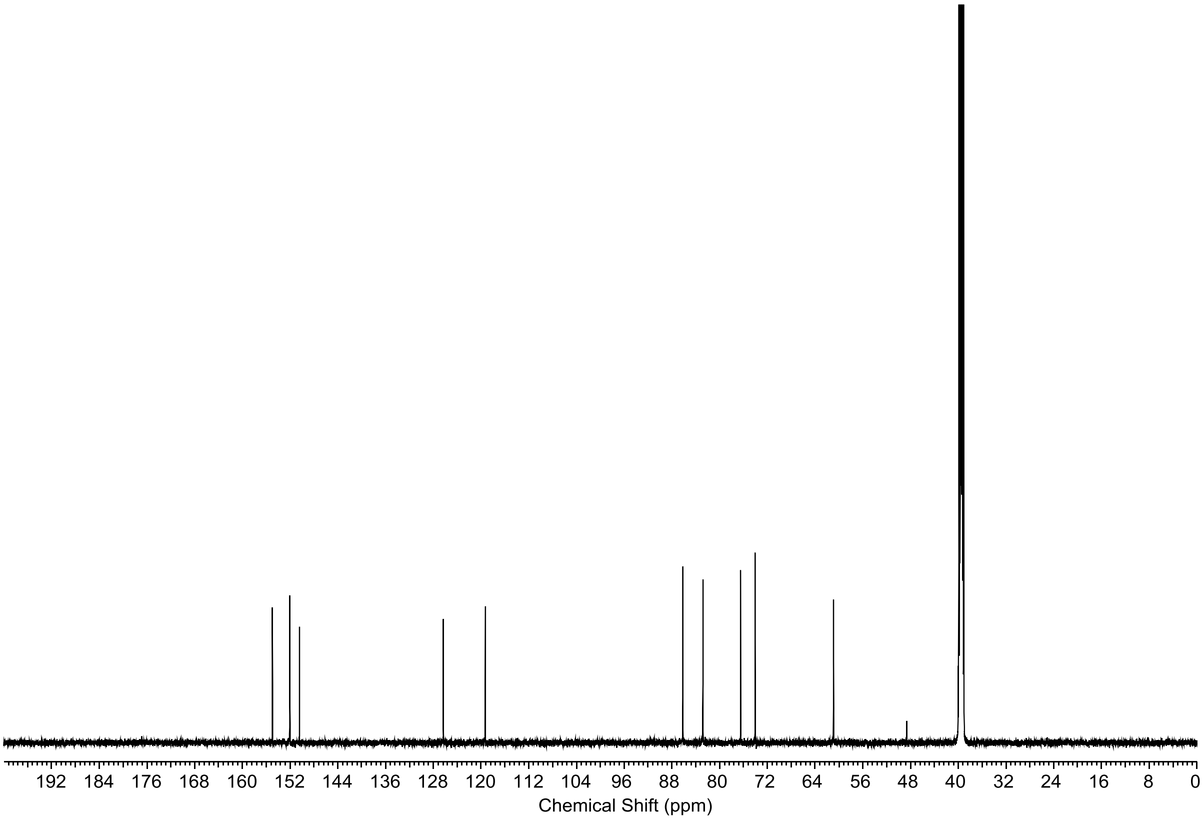

Supplementary Fig. 74. *– ^{1}H NMR (600 MHz, D_6-DMSO, 0-9 ppm, Top) and ^{13}C NMR (151 MHz, D_6-DMSO, 0-200 ppm, Bottom) spectra of 8-bromoadenine-9-β-arabinofuranoside (**27**).*

Supplementary References

[1] Covington, A. K., Paabo, M., Robinson, R. A., Bates, R. G. Use of the glass electrode in deuterium oxide and the relation between the standardized pD (paD) scale and the operational pH in heavy water. *Anal. Chem.* **40**, 700–706 (1968).

[2] Girniene, O., Gueyrard, D., Tatibouët, A., Sackus, A. & Rollin, P. Base-modified nucleosides from carbohydrate derived oxazolidinethiones: a five-step process. *Tetrahedron Lett.* **42**, 2977–2980 (2001).

[3] Sanchez, R. A. & Orgel L. E. Studies in prebiotic synthesis: V. Synthesis and photoanomerization of pyrimidine nucleosides. *J. Mol. Biol.* **47**, 531–543 (1970).

[4] Kovács, J., Pintér, I., Lendering, U., Köll, P. Transformation of aldoses into glycosylamine 1,2-(cyclic carbamates) (glyco-oxazolidin-2-ones) by reaction with potassium cyanate. *Carb. Res*. **210**, 155–166 (1991).

[5] *EU Pat*., EP1007509, 2000.

[6] Davidson, R. M., Byrd, G. D., White, E., Samm, V., Margolis, A. & Coxon, B. ^{1}H, ^{13}C and ^{15}N NMR studies of ^{13}C and ^{15}N labeled 2-methylthiooxazoline derivatives of pentoses and hexoses. Stereoelectronic effects on chemical shifts and mass fragmentation pathways. *Magn. Reson. Chem*. **24**, 929–937 (1986).

[7] Lin, T. S., Cheng, J. C., Ishiguro, K., Sartorelli A. C. Purine and 8-substituted purine arabinofuranosyl and ribofuranosyl nucleoside derivatives as potential inducers of the differentiation of the Friend erythroleukemia. *J. Med. Chem.* **28**, 1481–1485 (1985).

[8] Neidle, S., Taylor, G. L. & Cowling, P. C. The crystal and molecular structure of 8,2'-cycloadenosine trihydrate. *Acta. Cryst.* **B35**, 708–712 (1979).

[9] Bernasconi, C. F., Pérez-Lorenzo, M., & Brown, S. D. Kinetics of the Deprotonation of Methylnitroacetate by Amines: Unusually High Intrinsic Rate Constants for a Nitroalkane. *J. Org. Chem.* **72**, 4416-4423 (2007).

[10] Raulin, F., Fonsalas, F. & Wolny, M. Aminomalononitrile: Some new data of prebiotic interest*. Origins Life Evol. Biosphere.* **14***,* 151 (1984).

[13] Chattopadhyaya, J. B. & Reese, C. B. Interconversion of 8,2'-*O*-cycloadenosine and 2',3'-anhydro-8-oxyadenosine**.** *J. Chem. Soc., Chem. Commun*. 860–862 (1976).

[14] Ikehara, M. & Ogiso, Y. Studies of nucleosides and nucleotides—LIV: Purine cyclonucleosides—19. Further investigations on the cleavage of the 8,2'-O-anhydro linkage. A new synthesis of 9-β-D-arabinofuranosyladenine. *Tetrahedron* **28**, 3695 (1972).

[15] Powner, M. W. & Sutherland, J. D. Phosphate-mediated interconversion of *ribo*- and *arabino*-configured prebiotic nucleotide intermediates. *Angew. Chem. Int. Ed.* **49**, 4641–4643 (2010).
